# Structurally Diverse Polyketides From the Mangrove-Derived Fungus *Diaporthe* sp. SCSIO 41011 With Their Anti-influenza A Virus Activities

**DOI:** 10.3389/fchem.2018.00282

**Published:** 2018-07-12

**Authors:** Xiaowei Luo, Jie Yang, Feimin Chen, Xiuping Lin, Chunmei Chen, Xuefeng Zhou, Shuwen Liu, Yonghong Liu

**Affiliations:** ^1^CAS Key Laboratory of Tropical Marine Bio-Resources and Ecology, Guangdong Key Laboratory of Marine Materia Medica, South China Sea Institute of Oceanology, Chinese Academy of Sciences, Guangzhou, China; ^2^University of Chinese Academy of Sciences, Beijing, China; ^3^Guangdong Provincial Key Laboratory of New Drug Screening, Guangzhou Key Laboratory of Drug Research for Emerging Virus Prevention and Treatment, School of Pharmaceutical Sciences, Southern Medical University, Guangzhou, China; ^4^State Key Laboratory of Organ Failure Research, Southern Medical University, Guangzhou, China

**Keywords:** *Diaporthe* sp., polyketides, cytosporones, phthalides, anti-influenza A virus

## Abstract

Influenza A virus (IAV) is a severe worldwide threat to public health and economic development due to its high morbidity and mortality. Marine-derived fungi have been evidenced as a prolific source for the discovery of pharmacologically-active lead compounds. During the course of our search for novel bioactive substances from marine microorganisms, six new polyketides, including two octaketides (**1–2**), one chromone derivative (**13**), two highly substituted phthalides (**17–18**), and one α-pyrone derivative (**21**) along with 22 known congeners were isolated from a mangrove-associated fungus *Diaporthe* sp. SCSIO 41011. Their structures were determined by spectroscopic analysis and by comparison with literature data. And the absolute configurations were established according to the specific rotation or electron circular dichroism method. Antiviral evaluation results revealed that compounds **14**, **15**, **26**, and 5-chloroisorotiorin displayed significant anti-IAV activities against three influenza A virus subtypes, including A/Puerto Rico/8/34 H274Y (H1N1), A/FM-1/1/47 (H1N1), and A/Aichi/2/68 (H3N2), with IC_50_ values in the range of 2.52–39.97 μM. The preliminary structure-activity relationships (SARs) are also discussed. These findings expand the chemical and bioactive diversity of polyketides derived from the genus *Diaporthe*, and also provide a basis for further development and utilization of chromone, xanthone, and chloroazaphilone derivatives as source of potential anti-viral chemotherapy agents.

## Introduction

Polyketides represent an important category of secondary metabolites with great structural diversity from simple aromatics to highly modified complex architectures, such as macrolides, polyphenols, polyethers, polyenes, and enediynes (Fujii, 2010; Zheng et al., 2015). Distributing broadly in microbial origins, they are constructed by combination of iterative polyketide synthases (PKSs) and multifunctional and iterative oxygenases (Hang et al., 2016). In addition, polyketides play a vital role in modern medicine due to their diverse pharmacological features, such as lovastatin, a well-known fungal polyketide statins functioned as a cholesterol-lowering agent (Crawford and Townsend, 2010). Belonging to the family of octaketides, biosynthetically related cytosporones, dothiorelones and phomopsins are characterized with a di-/tri-hydroxybenzene lactone or a resorcinol scaffold harboring an *n*-heptane substituent, which were mainly encountered in endophytic fungi of several genera, such as *Phomopsis* (Kornsakulkarn et al., 2015; Kongprapan et al., 2017; Tan et al., 2017), *Diaporthe* (Brady et al., 2000; Liu et al., 2018), *Cytospora* (Brady et al., 2000; Abreu et al., 2010), *Pestalotiopsis* (Xu et al., 2009b). Of special note, cytosporone B (**7**) was reported as a nuclear orphan receptor Nur77 agonist as a promising therapeutic drug for cancers and hypoglycemia (Zhan et al., 2008), as well as the transcription factor NR4A1 agonist to control IAV infection and improve pulmonary function in infected mice (Xia et al., 2013; Egarnes et al., 2017), which had aroused a great interest for chemical synthesis study (Von Delius et al., 2017).

Influenza A virus (IAV), a negative sense RNA virus, is one of the main causes of human acute respiratory diseases characterized with high morbidity and mortality, posing a serious threat to public health and economic development (Liu et al., 2017). IAVs repeatedly circulate in many animal hosts, such as humans, birds, horses, dogs, and pigs, which can be subtyped to two envelope proteins: haemagglutinin (HA) and neuraminidase (NA) glycoproteins according to the antigenic properties (Medina and Garcia-Sastre, 2011). In 2009, the pandemic influenza H1N1 virus rapidly spread to 214 countries around the world, causing human infection and acute respiratory illness of more than 17,700 deaths (Bautista et al., 2010). As of 25th April 2018, there have been reported 1625 confirmed cases of human H7N9 infection and 623 deaths since 2013 according to the World Health Organization (http://www.fao.org/ag/againfo/programmes/en/empres/H7N9/situation_up-date.html). However, two families of antiviral drugs are hitherto currently used to treat human IAV infections, which are NA inhibitors, like zanamivir and oseltamivir, and inhibitors of the viral M2 protein exemplified by amantadine and rimantadine (Medina and Garcia-Sastre, 2011; Song et al., 2015). Due to the emergence of drug-resistant viral strains, there is an urgent development for novel classes of anti-IAV agents with new mode of action.

**Figure 1:**
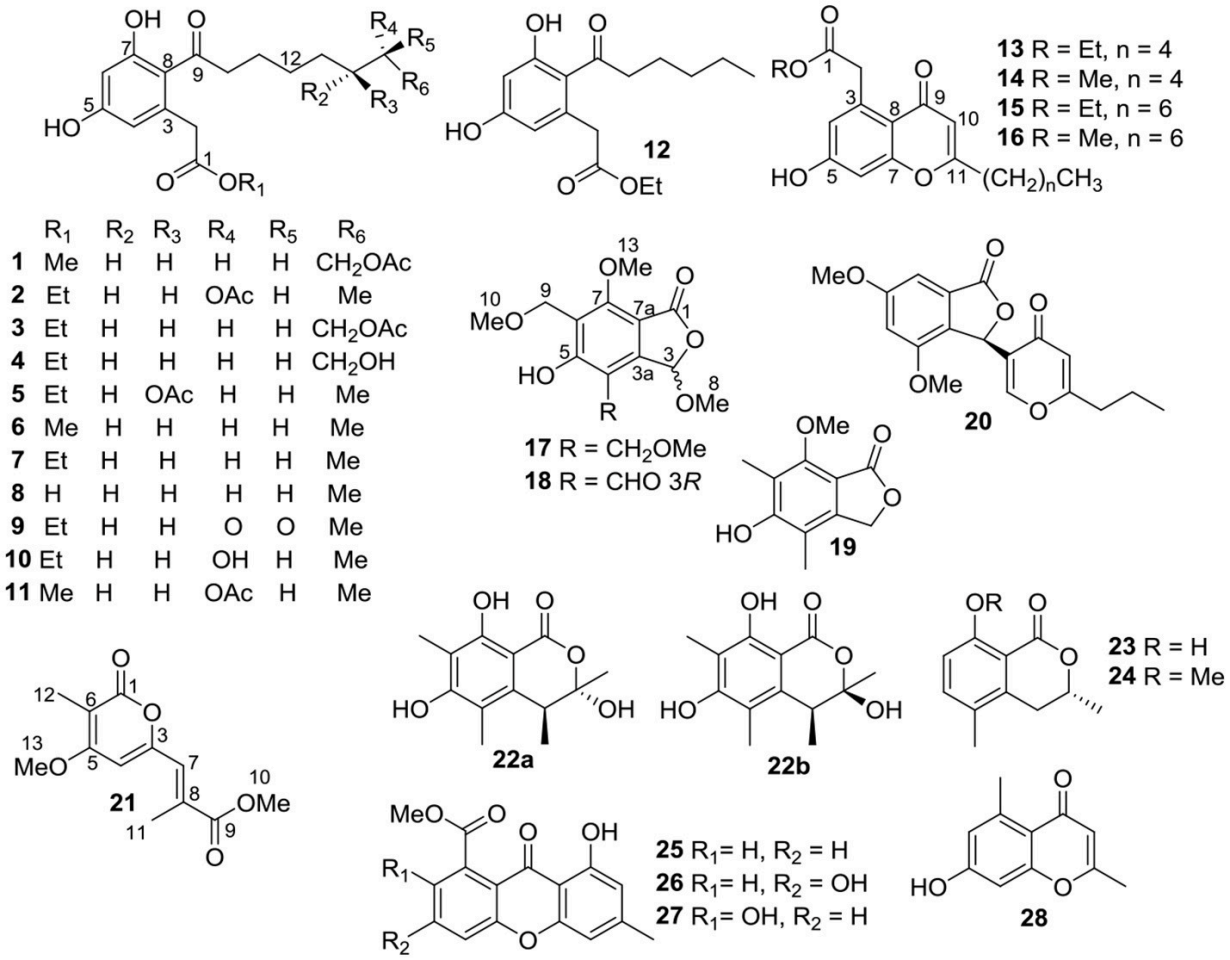
Structures of compounds **1**–**28**.

Marine-derived fungi are reported as a prodigious source of development for new antivirals against different important viruses (Moghadamtousi et al., 2015). In our continuing endeavor to discover biologically active compounds from marine microbes, a series of structurally interesting and biologically active natural products have been described (Luo et al., 2017; Tan et al., 2018). Recently, six new cytotoxic chloroazaphilone derivatives, isochromophilones A–F, have been isolated from the fungus *Diaporthe* sp. SCSIO 41011, an endophytic fungus obtained from the fresh tissue of the marine mangrove plant *Rhizophora stylosa* (Luo et al., 2018). Subsequent chemical investigations on the remaining fractions of the fungus led to the isolation of structurally diverse aromatic polyketides, including octaketides (dothiorelones or cytosporones), phthalides, chromones, xanthones, etc. (Figure 1). The structures of these compounds were determined by physicochemical properties and spectral data analysis as well as comparison with those reported in the literature. All the compounds were examined for anti-IAV activities against three influenza A virus subtypes, including A/Puerto Rico/8/34 H274Y (H1N1), A/FM-1/1/47 (H1N1), and A/Aichi/2/68 (H3N2). Details of the isolation, structure elucidation, and biological activity of these compounds, as well as preliminary SARs, are reported herein.

## Materials and methods

### General experimental procedures

Semi-preparative HPLC was performed on a Hitachi Primaide apparatus using an ODS (octadecylsilanized silica) column (YMC-pack ODS-A, YMC Co. Ltd., 10 × 250 mm, 5 μm, 2.5 mL/min). Chiral HPLC separation was performed using CHIRALPAK IC column (250 × 4.6 mm, 5 μm). TLC and column chromatography (CC) were performed on plates precoated with silica gel GF254 (10–40 μm) and over silica gel (200–300 mesh) (Qingdao Marine Chemical Factory), and Sephadex LH-20 (Amersham Biosciences), respectively. Spots were detected on TLC (Qingdao Marine Chemical Factory) under 254 nm UV light. All solvents employed were of analytical grade (Tianjin Fuyu Chemical and Industry Factory). The NMR spectra were obtained on a Bruker Avance spectrometer (Bruker) operating at 500 and 700 MHz for ^1^HNMR, 125 MHz, and 175 MHz for ^13^CNMR, using TMS as an internal standard. Optical rotations were acquired using a Perkin Elmer MPC 500 (Waltham) polarimeter. HRESIMS and ESIMS spectra data were recorded on a MaXis quadrupole-time-of-flight mass spectrometer and an amaZon SL ion trap mass spectrometer (Bruker), respectively. UV spectra were recorded on a Shimadzu UV-2600 PC spectrometer (Shimadzu). ECD spectra were performed on a Chirascan circular dichroism spectrometer (Applied Photophysics). IR spectra were measured on an IR Affinity-1 spectrometer (Shimadzu). The artificial sea salt was a commercial product (Guangzhou Haili Aquarium Technology Company).

### Fungal material

The fungus *Diaporthe* sp. SCSIO 41011 had the same origination as that in our recent published paper (Luo et al., 2018). A voucher specimen was deposited in the CAS Key Laboratory of Tropical Marine Bio-resources and Ecology, South China Sea Institute of Oceanology, Chinese Academy of Sciences, Guangzhou, China.

### Extraction and isolation

The fermentation of the strain and the extraction and isolation of its extract was identical to that of our recent published paper (Luo et al., 2018). Briefly, nine fractions was isolated from the EtOAc extract (150 g) by silica gel vacuum liquid chromatography (VLC) using step gradient elution with petroleum ether/CH_2_Cl_2_ (0–100%). Compounds **23** (40 mg, *t*_R_ 30 min) and **25** (4.5 mg, *t*_R_ 18 min) were purified from Fr.2 (13 g) by an ODS column (MeOH/H_2_O: 10–100%) and followed by semiprep-HPLC with 72% (2.5 mL/min) and 50% MeCN (2.3 mL/min) elutions, respectively. Fr.3 (10 g) was purified on Sephadex LH-20 (MeOH), an ODS column (MeOH/H_2_O: 10–100%) and finally semiprep-HPLC (70% MeCN, 2.3 mL/min) to afford compounds **17** (4.2 mg, *t*_R_ 7 min) and **24** (3.5 mg, *t*_R_ 9 min). Fraction 4 (12 g) was subjected to Sephadex LH-20 (MeOH) to provide eight subfractions (Frs.4-1~4-8). Then ten subfractions (Frs.4-3-1~4-3-10) were obtained from Fr. 4-3 by an ODS column (MeOH/H_2_O: 10–100%). Compound **18** (150 mg) was isolated from Fr. 4-3-4 by recrystallization. Meanwhile, compounds **20** (7.1 mg, *t*_R_ 24 min), **14** (4.3 mg, *t*_R_ 15 min) and **6** (141 mg, *t*_R_ 25 min) were isolated from Frs. 4-3-6, 4-3-7, 4-3-8 by semiprep-HPLC with 55% MeOH (2.5 mL/min), 50% MeCN (2.3 mL/min), and 68% MeCN (1.4 mL/min) elutions, respectively. Besides, compounds **27** (2 mg, *t*_R_ 10 min) and **26** (2.2 mg, *t*_R_ 12 min) was purified from Fr.4-4 and Fr. 4-6 by semiprep-HPLC with 80% MeOH (2.5 mL/min) and 60% MeCN (2.3 mL/min) elutions, respectively. Fraction 5 (4 g) was subjected to Sephadex LH-20 (MeOH) to provide five subfractions (Frs.5-1~5-5). Fr. 5-4 was further purified on semiprep-HPLC by 66% MeCN (2.5 mL/min) to give compounds **8** (77 mg, *t*_R_ 9 min), **12** (4 mg, *t*_R_ 11.5 min), and **7** (142 mg, *t*_R_ 20.6 min). Seven subfractions (Frs.7-1~7-7) were obtained from Fr. 7 (4 g) by Sephadex LH-20 (MeOH). Then Fr. 7-4 was subjected to an ODS column (MeOH/H_2_O: 10–100%) to provide thirteen subfractions (Frs.7-4-1~7-4-13). Fr. 7-4-8 was further purified on semiprep-HPLC by 47% MeCN (2.5 mL/min) to give compounds **4** (2.3 mg, *t*_R_ 12 min), **10** (2 mg, *t*_R_ 16 min), and **2** (3.8 mg, *t*_R_ 39 min), along with one subfraction (Fr. 7-4-8-6). Compounds **11** (4.7 mg, *t*_R_ 42 min) and **1** (24 mg, *t*_R_ 45 min) were purified from Fr. 7-4-8-6 by semiprep-HPLC by 56% MeOH (2.3 mL/min). Fr. 7-4-12 was subjected to semiprep-HPLC (74%MeOH, 1.8 mL/min) to give compounds **16** (2.6 mg, *t*_R_ 14 min) and **15** (3.8 mg, *t*_R_ 45 min). Similarly, Fr. 7-5 was subjected to an ODS column (MeOH/H_2_O: 10–100%) to provide eight subfractions (Frs.7-5-1~7-5-8). Compounds **19** (5.6 mg, *t*_R_ 10.4 min) and **28** (5 mg, *t*_R_ 8 min) were purified from Fr.7-5-5 and Fr.7-5-6 by semiprep-HPLC with 50% MeCN (2.5 mL/min) and 47% MeCN (2.5 mL/min) elutions, respectively. Fr. 7-5-7 was subjected to semiprep-HPLC (50%MeCN, 2.5 mL/min) to afford compounds **22** (5 mg, *t*_R_ 11.5 min) and **21** (1.5 mg, *t*_R_ 15 min). Fr.9 (7 g) was subjected to Sephadex LH-20 (MeOH) and followed by an ODS column (MeOH/H_2_O: 10–100%) to provide six subfractions (Frs.9-1-1~9-1-6). Fr. 9-1-4 was purified by semiprep-HPLC (55%MeCN, 2.0 mL/min) to provide compounds **9** (3 mg, *t*_R_ 12 min), **5** (3 mg, *t*_R_ 25 min), **3** (2 mg, *t*_R_ 28 min). Compund **13** (0.8 mg, *t*_R_ 10 min) was isolated from Fr.9-1-5 by semiprep-HPLC (78% MeOH, 2.0 mL/min).

### Antiviral activity

All the isolated compounds (**1**–**28**), along with recently reported co-isolated 5-chloroisorotiorin (Luo et al., 2018), were screened for their anti-IAV activities according to the previously reported 3-(4,5-dimethylthiazol-2yl)-2,5-diphenyltetrazolium bromide (MTT) colorimetric assay, using ribavirin as a positive control (Li et al., 2017; Yang et al., 2018). In brief, Madin Derby canine kidney (MDCK) cells were cultured in Dulbecco's modified Eagle's medium (DMEM) supplemented with 10% fetal bovine serum and 1% penicillin/streptomycin. Meanwhile, different influenza A virus subtypes, including A/Puerto Rico/8/34 H274Y (H1N1), A/FM-1/1/47 (H1N1), and A/Aichi/2/68 (H3N2), were multiplied in 10-day-old chick embryo at 37°C. The cytotoxicity of the compounds was also evaluated by the MTT assay. Briefly, approximately 90% confluent cells in 96-well plates were exposed to the compounds at 2-fold serial dilutions. After 48 h of incubation, 100 μL of MTT solution, which was diluted by the medium to 0.5 mg/mL, was added and retained at 37°C for 4 h. Then the supernatant was removed, followed by the addition of 150 μL of dimethyl sulfoxide (DMSO) to dissolve the formazan product. The optical density for each well was measured on the Tecan Genios Pro microplate reader (Bedford, MA, USA) at 570 nm. To determine the antiviral activities of the compounds, confluent MDCK cells were infected with the virus at multiplicity of infection (MOI) of 0.01 at 37°C for 1 h. The compounds of non-cytotoxic concentrations were then added to the cells after washing away the unabsorbed virus with phosphate-buffered saline (PBS), and the cells were cultured for another 48 h. At the end of the culture, the MTT-based assay as previously described was assessed for the antiviral activity of the isolated compounds.

### Statistical analysis

All statistical analysis of the data were processed by GraphPad Prism. The results are presented as the mean ± standard deviation (SD) from experiments in triplicate. Student's *t*-test was used to analyze the statistical significance between two groups, more groups by one-way ANOVA with or without Tukey–Kramer multiple comparison. A *p* < 0.05 was regarded as statistically significant.

## Results and discussion

### Identification of compounds

The endophytic fungus *Diaporthe* sp. SCSIO 41011 was cultured on solid rice medium for 60 days. The EtOAc extract (150 g) of the fermentation was separated by continuously silica gel chromatography and semi-preparative HPLC chromatography to yield 28 aromatic polyketides (**1**–**28**). Their chemical structures were determined by comprehensive spectroscopic analyses or comparison with those reported data.

**Figure 2:**
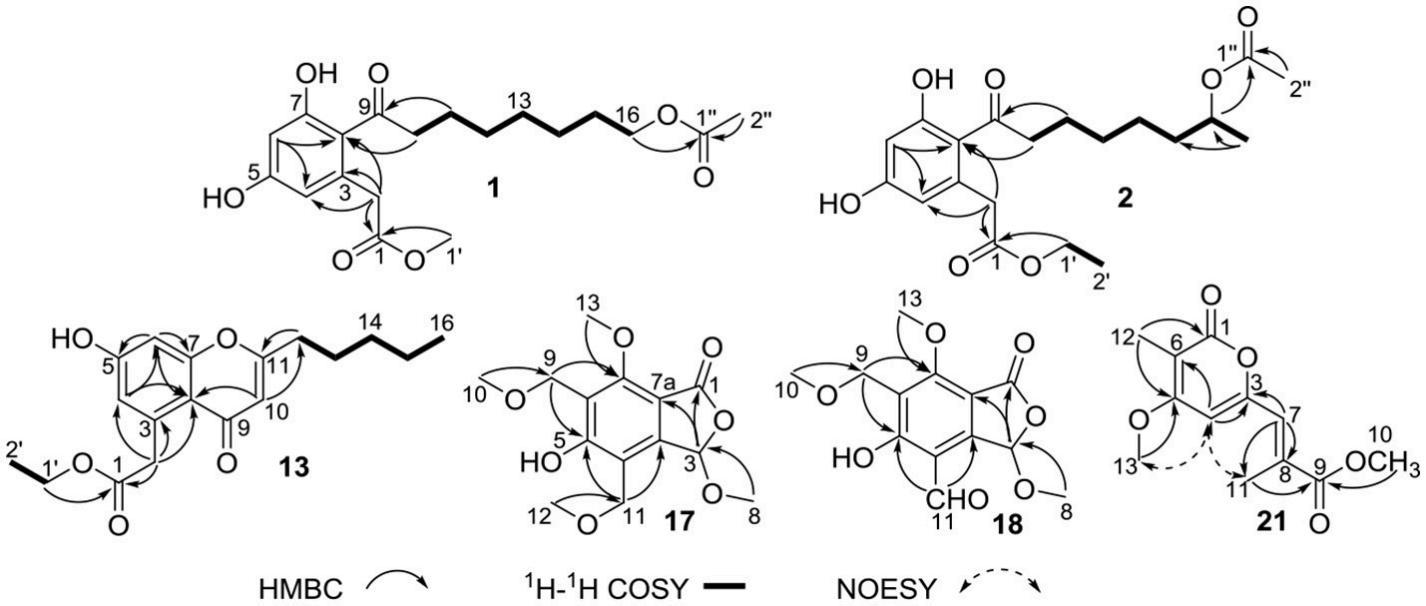
Key HMBC, ^1^H-^1^H COSY, and NOESY correlations of compounds **1**–**2**, **13**, **17**–**18**, and **21**.

Compound **1** was obtained as colorless oil and had the molecular formula C_19_H_26_O_7_ as determined by a deprotonated ion peak at *m/z* 365.1607 (calcd for C_19_H_25_O_7_, 365.1600) in HRESIMS data. The ^1^H NMR data (Table 1) along with HSQC experiment of **1** displayed two singlet methyls at δ_H_ 2.01 (3H, s) and 3.65 (3H, s), two aromatic protons at δ_H_ 6.26 (1H, d, *J* = 2.2 Hz) and 6.19 (1H, d, *J* = 2.2 Hz), along with an array of methylene signals. The ^13^C NMR spectrum (Table 1) showed 19 resonances that were sorted by a distortionless enhancement by polarization transfer (DEPT) experiment, assigned to three carbonyls (δ_C_ 209.0, 174.0, and 173.1), six aromatic carbons (δ_C_ 161.8, 160.1, 137.0, 120.9, 112.0, and 102.9), eight methylenes (δ_C_ 65.7, 45.0, 40.4, 30.3, 30.1, 29.6, 26.8, and 25.4) and two methyls (δ_C_ 52.3 and 20.8). The aforementioned NMR data showed **1** was closely related structurally to the co-isolated 16-acetoxydothiorelone C (**3**) (Liu et al., 2018). The only difference was the appearance of a methyl group (δ_H/C_ 3.65/52.3) at C-1′ in **1** instead of an ethyl group in **3**, which was also verified by the HMBC correlation from H_3_-1′ to C-1 (Figure 2). Thus, the structure of **1** was determined as shown in Figure 1 and assigned the trivial name dothiorelone O.

**Table 1:** ^1^H and ^13^C NMR spectral data of compounds **1**, **2**, and **13** in CD_3_OD.

	**1**		**2**		**13**	
**No**.	**δ_H_*^*a*^***	**δ_C_, type*^*b*^***	**δ_H_*^*c*^***	**δ_C_, type*^*d*^***	**δ_H_*^*c*^***	**δ_C_, type*^*d*^***
1		174.0, C		173.6, C		173.6, C
2	3.58, s	40.4, CH_2_	3.60, s	40.6, CH_2_	4.06, s	42.1, CH_2_
3		137.0, C		137.1, C		138.5, C
4	6.19, d (2.2)	112.0, CH	6.21, d (2.2)	111.9, CH	6.65, d (2.2)	120.3, CH
5		161.8, C		161.6, C		164.8, C
6	6.26, d (2.2)	102.9, CH	6.28, d (2.2)	102.8, CH	6.73, d (2.2)	103.1, CH
7		160.1, C		160.0, C		161.5, C
8		120.9, C		121.1, C		115.1, C
9		209.0, C		208.9, C		181.4, C
10	2.85, t (7.6)	45.0, CH_2_	2.83, t (7.6)	43.3, CH_2_	5.98, s	110.3, CH
11	1.61, m	25.4, CH_2_	1.62, o	25.3, CH_2_		170.3, C
12	1.34, o	30.3, CH_2_	1.35, o	30.2, CH_2_	2.61, t (7.1)	34.5, CH_2_
13	1.34, o	30.1, CH_2_	1.35, o	26.3, CH_2_	1.73, m	27.6, CH_2_
14	1.34, o	26.8, CH_2_	1.52, m	36.8, CH_2_	1.39, o	32.3, CH_2_
15	1.61, m	29.6, CH_2_	4.86, o	72.4, CH	1.39, o	23.4, CH_2_
16	4.04, t (7.1)	65.7, CH_2_	1.22, d (7.1)	20.2, CH_3_	0.93, t (7.1)	14.2, CH_3_
1′	3.65, s	52.3, CH_2_	4.14, q (7.1)	61.8, CH_2_	4.13, q (7.1)	61.7, CH_2_
2′			1.26, t (7.1)	14.5, CH_3_	1.24, t (7.1)	14.5, CH_3_
1^′′^		173.1, C		172.8, C		
2^′′^	2.01, s	20.8, CH_3_	2.02, s	21.2, CH_3_		

a *In 500 MHz*.

b *In 125 MHz*.

c *In 700 MHz*.

d *In 175 MHz, m, multiplet; o, overlapped*.

Compound **2** was also acquired as colorless oil and was determined to have the molecular formula C_20_H_28_O_7_ from the HRESIMS data. Inspection of the comprehensive spectral data of **2**, including MS, and 1D, 2D-NMR data, indicated that **2** shared the same planer structure with (15S)-acetoxydothiorelone A (Liu et al., 2018). Comparison of the specific rotations between **2** ([α]${}_{D}^{25}$ = −5.8 (c 0.10, MeOH)) and (15S)-acetoxydothiorelone A ([α]${}_{D}^{20}$ = +4.9 (c 0.25, MeOH)) suggested **2** had the 15R configuration (Izuchi et al., 2011; Beekman and Barrow, 2015; Liu et al., 2018), given that they possessed only one chiral carbon at C-15. Therefore, the structure of **2** was resolved and accordingly named (15R)-acetoxydothiorelone A.

Compound **13** was also isolated as colorless oil and gave a molecular formula of C_18_H_22_O_5_ relied on a deprotonated ion peak at *m/z* 317.1403 (calcd for C_18_H_21_O_5_, 317.1389) in the HRESIMS spectrum. The ^1^H NMR data (Table 1) together with HSQC and HMBC spectra of **13** displayed signals indicative of two doublet methyls at δ_H_ 0.93 (3H, d, *J* = 7.1 Hz) and 1.24 (3H, d, *J* = 7.1 Hz), three aromatic protons at δ_H_ 6.65 (1H, d, *J* = 2.2 Hz), 6.73 (1H, d, *J* = 2.2 Hz) and 5.98 (1H, s), along with several methylenes. The ^13^C NMR spectrum (Table 1) showed 18 resonances attributable to two carbonyls (δ_C_ 173.6 and 181.4), six aromatic carbons (three oxygenated ones), six methylenes (δ_C_ 61.7, 42.1, 34.5, 32.3, 27.6, and 23.4) and two methyls (δ_C_ 14.2 and 14.5). The above mentioned spectral characteristics were closely consistent to those of the co-isolated pestalotiopsone F (**14**) (Xu et al., 2009a), but suggested the appearance of an ethyl group (δ_H/C_ 4.13/61.7 at C-1′ and 1.24/14.5 at C-2′) in **13** rather than a methyl group (δ_H/C_ 3.66/52.2) at C-1′ in **14**. These changes were also ascertained by the HMBC correlations from H_3_-1′ to C-1 and from H_3_-2′ to C-1′, as well as the ^1^H-^1^H COSY correlation of H_3_-2′ and H_2_-1′ (Figure 2). Based on the above discussion, the structure of **13** was elucidated and the trivial name pestalotiopsone H was assigned.

Compound **17** was obtained as a white solid and its molecular formula was found to be C_14_H_18_O_7_ on the basis of HRESIMS and NMR data. The ^1^H NMR data (Table 2) of **17** revealed four *O*-methyls at δ_H_ 3.38, 3.38, 3.54, and 3.99, two singlet methylenes at δ_H_ 4.60 and 4.65, and a hemiketal methine at δ_H_ 6.29. In addition to the above seven corresponding hydrogen-bearing carbons, seven carbons remained in the ^13^C NMR spectrum, including one carbonyl (δ_C_ 169.6), six aromatic carbons [(δ_C_ 111.4, 119.0, 121.4, 148.9), and two oxygenated ones at δ_C_ 160.7 and 174.3]. The foregoing spectroscopic data showed great similarity to that of microsphaerophthalide F (Sommart et al., 2012) except that a methyl group (δ_H/C_ 2.16/8.5) located at C-6 in microsphaerophthalide F was replaced by an ethoxyl group (δ_H/C_ 4.60/65.3 at C-9 and 3.38/58.1 at C-10) anchored at C-6 in **17**. This deduction was also supported by the HMBC correlations from H_3_-10 to C-9 and from H_2_-9 to C-5 and C-7. The barely measurable optical rotation value and quite weak Cotton effects in the ECD spectrum suggested **17** was racemic, which was also confirmed by the chiral HPLC analysis with two peaks (peak area ratio: 1:1) in the HPLC profile (Supplementary Materials). However, the quantity of **17** was too little to perform further resolution. Hence, the structure of compound **17** was elucidated and the trivial name (±)-microsphaerophthalide H was assigned.

**Table 2:** ^1^H and ^13^C NMR spectral data of compounds **17**, **18** and **21** (^1^H for 700 MHz, ^13^C for 175 MHz).

	**17**^***a***^		**18**^***b***^		**21**^***a***^	
**No**.	**δ_H_**	**δ_C_, type**	**δ_H_**	**δ_C_, type**	**δ_H_**	**δ_C_, type**
1		169.6, C		164.7, C		166.3, C
2						
3	6.29, s	103.1, CH	6.75, s	100.7, CH		158.8, C
3a		148.9, C		151.7, C		
4		119.0, C		112.4, C	6.87, d (1.4)	98.8, CH
5		174.3, C		166.3, C		167.8, C
6		121.4, C		120.9, C		105.6, C
7		160.7, C		163.1, C	6.67, s	120.3, CH
7a		111.4, C		109.6, C		
8	3.54, s	56.1, CH_3_	3.60, s	56.9, CH_3_		144.5, C
9	4.60, s	65.3, CH_2_	4.44, s	61.3, CH_2_		168.0, C
10	3.38, s	58.1, CH_3_	3.27, s	57.7, CH_3_	3.76, s	52.0, CH_3_
11	4.65, s	67.2, CH_2_	10.14, s	192.5, CH	2.44, d (1.4)	13.6, CH_3_
12	3.38, s	58.4, CH_3_			1.92, s	8.8, CH_3_
13	3.99, s	63.0, CH_3_	4.14, s	63.4, CH_3_	4.02, s	57.5, CH_3_

a *In CD_3_OD*.

b *In DMSO-d_6_*.

**Figure 3:**
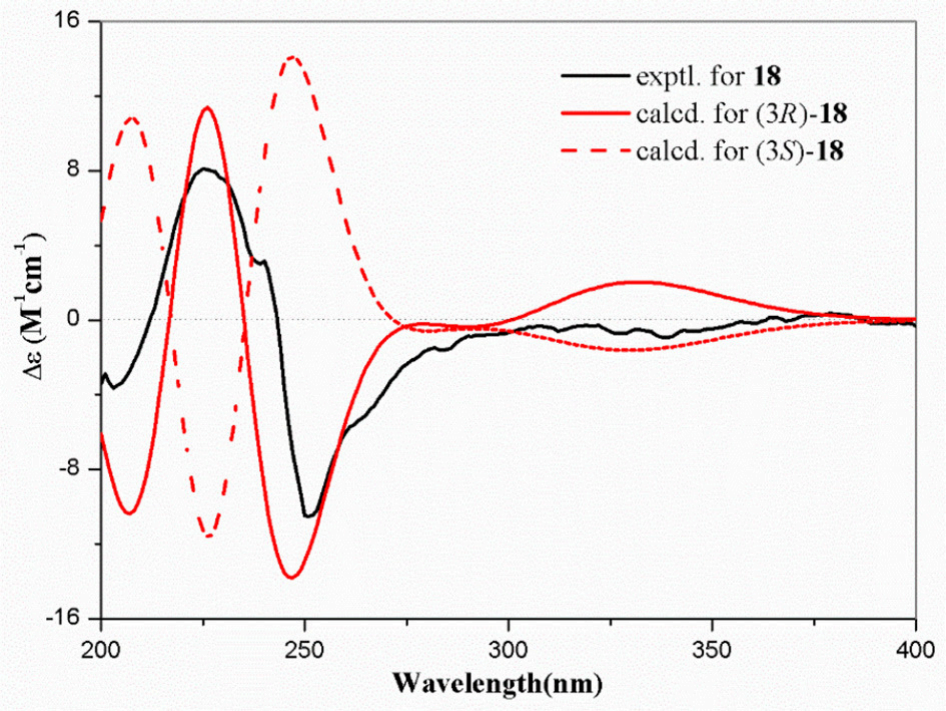
Experimental and calculated ECD spectra of **18**.

Compound **18** was obtained as colorless needle crystals and had the molecular formula C_13_H_14_O_7_, as evidenced by HRESIMS ([M+H]^+^, 283.0809; calcd for C_13_H_15_O_7_, 283.0818) and the NMR data. The highly similar NMR spectroscopic data of **18** to those of **17** indicated that their structures were closely related, except for the presence of an aldehyde group (δ_H/C_ 10.14/192.5) joined at C-4 in **18**, rather than an ethoxyl group (δ_H/C_ 4.65/67.2 and 3.38/58.4) anchored at C-4 in **17**. The absolute configuration of C-3 of **18** was mainly determined by comparison of the specific rotations with those reported data, as well as comparison between the computed and experimental ECD spectra. Among these reported 3-oxygenated phthalides, the 3*S*- and 3*R*- ones generally showed negative and positive specific rotations, respectively (Sommart et al., 2012). Thus, the positive sign of specific rotation [[α]${}_{D}^{25}$ = +25 (*c* 0.10, MeOH)] of **18** led to the deduction of 3*R* configuration in **18**, which was also confirmed from experimental and calculated ECD spectra of **18** as shown in Figure 3. Further chiral HPLC analysis confirmed that **18** was single enantiomer. Consequently, the structure of **18** was determined as shown in Figure 1 and termed microsphaerophthalide I. Notably, the 3-oxygenated phthalides are uncommon in natural sources.

Compound **21** was obtained as a white solid and had the molecular formula C_12_H_14_O_5_, as determined from HRESIMS ([M+H]^+^, 239.0917; calcd for C_12_H_15_O_5_, 239.0919) and the NMR data. Detailed analyses of its NMR spectroscopic features implied that it was closely related structurally to convolvulopyrone (Tsantrizos et al., 1992), but for the presence of an additional *O*-methyl group (δ_H/C_ 3.76/52.0) at C-10 in **21**, indicating that **21** was a methyl derivative of convolvulopyrone. Besides, compound **21** and convolvulopyrone shared the same relative configuration of 7*E* due to the absence of a NOESY correlation between H-7 and H_3_-11. As a result, the structure of **21** was determined and given the trivial name methyl convolvulopyrone. However, compound **21** was likely obtained as an artifact formed in the process of extraction and purification using MeOH as a solvent.

Besides, these co-isolated known congeners were elucidated by comparing their physicochemical properties and spectroscopic data with those reported literature values (Supplementary Materials). They were determined as 16-acetoxydothiorelone C (**3**) (Liu et al., 2018), dothiorelone C (**4**) (Liu et al., 2018), (14*R*)-acetoxydothiorelone B (**5**) (Liu et al., 2018), cytosporone N (**6**) (Beekman and Barrow, 2015), cytosporone B (**7**) (Brady et al., 2000), cytosporone A (**8**) (Brady et al., 2000), dothiorelone I (**9**) (Liu et al., 2018), (15*R*)-dothiorelone A (**10**) (Liu et al., 2018), methyl (*R*)-2-(2-(7-acetoxyoctanoyl)-3,5-dihydroxyphenyl)acetate (**11**) (Beekman and Barrow, 2015), secocurvularin (**12**) (Bracher and Krauss, 1998), pestalotiopsone F (**14**) (Xu et al., 2009a), pestalotiopsone B (**15**) (Xu et al., 2009a), pestalotiopsone A (**16**) (Xu et al., 2009a), 5-hydroxy-7-methoxy-4,6-dimethylphthalide (**19**) (Sommart et al., 2012), dihydrovermistatin (**20**) (Komai et al., 2005), sclerotinin A (**22**) (Lai et al., 2013), 3,5-dimethyl-8-hydroxy-3,4-dihydroisocoumarin (**23**) (Kokubun et al., 2003), 3,5-dimethyl-8-methoxy-3,4-dihydroisocoumarin (**24**) (Kokubun et al., 2003), methyl 8-hydroxy-6-methyl-9-oxo-9*H*-xanthene-1-carboxylate (**25**) (Lai et al., 2013), 3,8-dihydroxy-6-methyl-9-oxo-9*H*-xanthene-1-carboxylate (**26**) (Nguyen et al., 2017), pinselin (**27**) (Cui et al., 2016), 7-hydroxy-2,5-dimethylchromone (**28**) (Koenigs et al., 2010). Amongst, methyl (*R*)-2-[2-(7-acetoxyoctanoyl)-3,5-dihydroxyphenyl]acetate (**11**) was isolated as a naturally occurring compound for the first time. While sclerotinin A (**22**) was isolated as a diastereoisomeric mixture which could not be separated by RP-HPLC method (Lai et al., 2013). This research further enriched secondary metabolites in the genus *Diaporthe* and also expanded the chemical diversity of polyketides, such as dothiorelones, cytosporones, phthalides, chromones, etc.

### Antiviral activity

**Figure 4:**
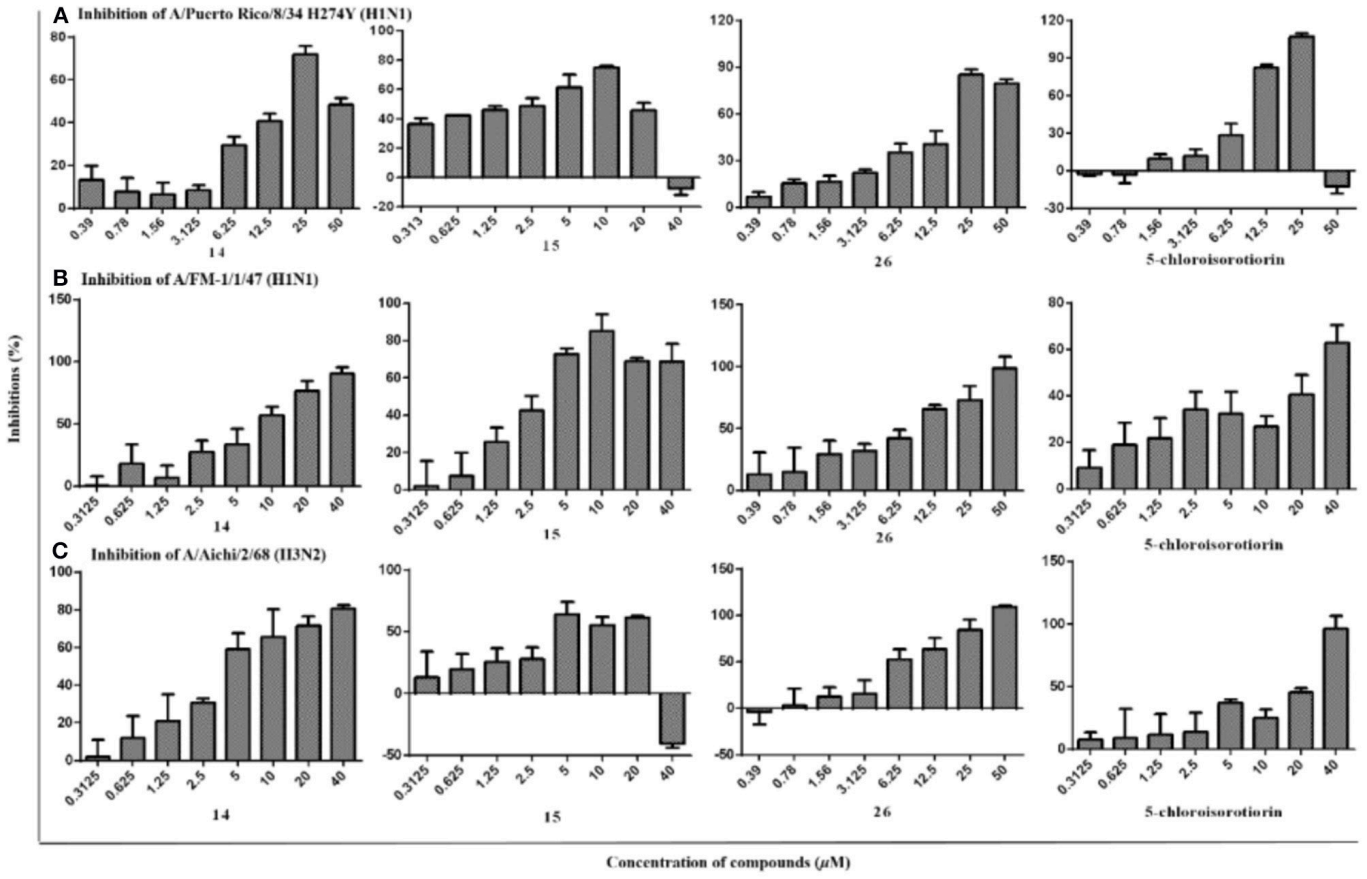
The inhibitory activities of compounds **14**, **15**, **26**, and 5-chloroisorotiorin against influenza A viruses, including A/Puerto Rico/8/34 H274Y (H1N1) (**A**), A/FM-1/1/47 (H1N1) (**B**), and A/Aichi/2/68 (H3N2) (**C**).

Antiviral effect of the isolated compounds **1**–**28** and 5-chloroisorotiorin against different IAV subtypes, including A/Puerto Rico/8/34 H274Y (H1N1), A/FM-1/1/47 (H1N1), and A/Aichi/2/68 (H3N2), were then evaluated. Those compounds showed nearly no cytotoxicity against MDCK cells (IC_50_ > 200 μM). Compounds **14**, **15**, **26**, and 5-chloroisorotiorin displayed significant anti-IAV activities against the above mentioned subtypes with IC_50_ values in the range of 2.52–39.97 μM (Table 3). However, the remaining compounds (**1**–**13**, **16**–**25**, **27**–**28**) were inactive toward the three aforementioned IAV subtypes. Furthermore, pestalotiopsone F (**14**) and 3,8-dihydroxy-6-methyl-9-oxo-9*H*-xanthene-1-carboxylate (**26**) exhibited obvious inhibition effect on A/FM-1/1/47 (H1N1), as well as A/Aichi/2/68 (H3N2), in a dose-dependent manner (Figure 4).

**Table 3:** Inhibition activity of compounds **1**–**28** and 5-chloroisorotiorin against Influenza A Virus strains.

**Compounds**	**IC**_**50**_ **(*****μ*****M)**^**a**^		
	**A/Puerto Rico/8/34 H274Y (H1N1)**	**A/FM-1/1/47 (H1N1)**	**A/Aichi/2/68 (H3N2)**
**14**	21.80 ± 7.96	6.74 ± 1.26	6.17 ± 1.46
**15**	2.56 ± 0.32	4.82 ± 1.90	6.76 ± 2.72
**26**	9.40 ± 1.96	4.80 ± 1.28	5.12 ± 1.49
5-chloroisorotiorin	2.52 ± 0.21	37.97 ± 15.11	10.10 ± 1.84
Remainings	IN^b^	IN	IN

a The samples were tested in triplicate, and the data are presented as the mean ± SD

b *Inactive*.

Additionally, based on comparison of the structural characteristics among these analogs, a preliminary structure-activity relationship is discussed. Pestalotiopsone F (**14**) exhibited selective inhibitions against the two IAV subtypes of A/FM-1/1/47 (H1N1) and A/Aichi/2/68 (H3N2) with the IC_50_ values of 6.74 ± 1.26 and 6.17 ± 1.46 μM, respectively. Comparing with the antiviral pestalotiopsones F (**14**) and B (**15**), the co-isolated siblings pestalotiopsones H (**13**) and A (**16**) were inactive toward the three IAV subtypes, which revealed methylation of the carboxyl group at C-1 for the pestalotiopsones with a C_7_ aliphatic branch located at C-11, and while ethylation of the carboxyl group at C-1 for the pestalotiopsones with a C_5_ aliphatic branch located at C-11, were essential for anti-IAV activities. Notably, among the three xanthones (**25**–**27**), only 3,8-dihydroxy-6-methyl-9-oxo-9*H*-xanthene-1-carboxylate (**26**) demonstrated remarkable inhibitory effects against A/FM-1/1/47 (H1N1), A/Puerto Rico/8/34 H274Y (H1N1), and A/Aichi/2/68 (H3N2) with IC_50_ values of 4.80 ± 1.28, 9.40 ± 1.96, and 5.12 ± 1.49 μM, respectively, which indicated the hydroxyl group at C-3 probably promote the anti-IAV activities toward the three subtypes. By the way, 5-chloroisorotiorin (Luo et al., 2018), a recently reported co-isolated chloroazaphilone derivative obtained with major amount, was also screened for anti-IAV activity, which presented selective inhibition activities against the two IAV subtypes of A/Puerto Rico/8/34 H274Y (H1N1) and A/Aichi/2/68 (H3N2) with the IC_50_ values of 2.52 ± 0.21 and 10.10 ± 1.84 μM, respectively. The remaining ones (**1**–**13**, **16**–**26**, **27**–**28**) showed no obvious inhibition against A/FM-1/1/47 (H1N1), A/Puerto Rico/8/34 H274Y (H1N1), and A/Aichi/2/68 (H3N2).

### Characterization of compounds

Dothiorelone O (**1**): colorless oil; UV (MeOH) λ_max_ (log ε) 302 (3.75), 269 (3.89), 220 (4.16), 204 (4.27) nm; IR (film) ν_max_ 3,356, 2,933, 2,833, 1,732, 1,714, 1,609, 1,591, 1,462, 1,261, 1,163, and 1,028 cm^−1^; ^1^H and ^13^C NMR data, Table 1; HRESIMS *m/z* 365.1607 [M–H]^−^ (calcd for C_19_H_25_O_7_, 365.1600), 401.1368 [M+Cl]^−^ (calcd for C_19_H_26_ClO_7_, 401.1367).

(15*R*)-acetoxydothiorelone A (**2**): colorless oil; [α]${}_{D}^{25}$ – 5.8 (*c* 0.10, MeOH); UV (MeOH) λ_max_ (log ε) 303 (3.69), 269 (3.78), 220 (4.14), 204 (4.17) nm; IR (film) ν_max_ 3,358, 2,933, 2,858, 1,732, 1,714, 1,609, 1,558, 1,456, 1,265, 1,249, 1,159, and 1,024 cm^−1^; ^1^H and ^13^C NMR data, Table 1; HRESIMS *m/z* 403.1741 [M+Na]^+^ (calcd for C_20_H_28_NaO_7_, 403.1733), 419.1470 [M+K]^+^ (calcd for C_20_H_28_KO_7_, 419.1472), 783.3563 [2M+Na]^+^ (calcd for C_40_H_56_NaO_14_, 783.3568).

Pestalotiopsone H (**13**): colorless oil; UV (MeOH) λ_max_ (log ε) 291 (4.14), 250 (4.31), 242 (4.28), 217 (4.38) nm; IR (film) ν_max_ 3,419, 2,927, 2,856, 1,732, 1,716, 1,645, 1,558, 1,456, 1,375, 1,274, 1,180, 1,161, and 1,028 cm^−1^; ^1^H and ^13^C NMR data, Table 1; HRESIMS *m/z* 317.1403 [M–H]^−^ (calcd for C_18_H_21_O_5_, 317.1389), 353.1167 [M+Cl]^−^ (calcd for C_18_H_22_ClO_5_, 353.1156), 635.2860 [2M–H]^−^ (calcd for C_36_H_43_O_10_, 635.2856), 671.2626 [2M+Cl]^−^ (calcd for C_36_H_44_ClO_10_, 671.2623).

(±)-microsphaerophthalide H (**17**): a white solid; [α]${}_{D}^{25}$ – 5 (*c* 0.06, MeOH); UV (MeOH) λ_max_ (log ε) 307 (4.06), 249 (4.05), 220 (4.48) nm; IR (film) ν_max_ 3,419, 2,935, 2,846, 1,747, 1,734, 1,604, 1,558, 1,448, 1,429, 1,375, 1,276, 1,201, 1,159, 1,093, and 1,074 cm^−1^; ^1^H and ^13^C NMR data, Table 2; HRESIMS *m/z* 299.1123 [M+H]^+^ (calcd for C_14_H_19_O_7_, 299.1131), 321.0954 [M+Na]^+^ (calcd for C_14_H_18_NaO_7_, 321.0950), 619.2010 [2M+Na]^+^ (calcd for C_28_H_36_NaO_14_, 619.2003).

Microsphaerophthalide I (**18**): colorless needle crystals; [α]${}_{D}^{25}$ = +25 (*c* 0.10, MeOH); UV (MeOH) λ_max_ (log ε) 367 (3.60), 295 (4.07), 245 (4.46), 201 (4.10) nm; ECD (0.15 mg/mL, MeOH) λ_max_ (Δε) 203 (−2.07), 225 (+4.62), 251 (−6.00) nm; IR (film) ν_max_ 3,419, 2,945, 2,885, 1,770, 1,749, 1,668, 1,653, 1,558, 1,489, 1,375, 1,338, 1,286, 1,205, 1,097, and 1,058 cm^−1^; ^1^H and ^13^C NMR data, Table 2; HRESIMS *m/z* 283.0809 [M+H]^+^ (calcd for C_13_H_15_O_7_, 283.0818), 305.0632 [M+Na]^+^ (calcd for C_13_H_14_NaO_7_, 305.0637).

Methyl convolvulopyrone (**21**): a white solid; UV (MeOH) λ_max_ (log ε) 343 (4.05), 279 (3.73), 239 (4.51), 205 (4.12) nm; IR (film) ν_max_ 3,446, 2,954, 2,927, 1,681, 1,635, 1,446, 1,355, 1,195, 1,182, 1,139, and 1,039 cm^−1^; ^1^H and ^13^C NMR data, Table 2; HRESIMS *m/z* 239.0917 [M+H]^+^ (calcd for C_12_H_15_O_5_, 239.0919), 261.0749 [M+Na]^+^ (calcd for C_12_H_14_NaO_5_, 261.0739), 499.1590 [2M+Na]^+^ (calcd for C_24_H_28_NaO_10_, 499.1580).

## Conclusions

Twenty-eight aromatic polyketides, including two new octaketides (**1–2**), one new chromone derivative (**13**), two new highly substituted phthalides (**17–18**), and one new α-pyrone derivative (**21**) along with 22 known congeners were isolated from a mangrove-associated fungus *Diaporthe* sp. SCSIO 41011, while methyl (*R*)-2-[2-(7-acetoxyoctanoyl)-3,5-dihydroxyphenyl]acetate (**11**) was isolated as a new natural compound and (±)-microsphaerophthalide H (**17**) was obtained as a racemic mixture. Amongst, pestalotiopsone F (**14**), pestalotiopsone B (**15**), 3,8-dihydroxy-6-methyl-9-oxo-9*H*-xanthene-1-carboxylate (**26**), and 5-chloroisorotiorin displayed pronounced anti-IAV activities against three IAV virus subtypes, including A/Puerto Rico/8/34 H274Y (H1N1), A/FM-1/1/47 (H1N1), and A/Aichi/2/68 (H3N2) with IC_50_ values in the range of 2.52–39.97 μM. This work further enriched secondary metabolites in the genus *Diaporthe* and also expanded the chemical and bioactive diversity of polyketides, such as dothiorelones, cytosporones, phthalides, chromones, etc. Furthermore, our findings provide a basis for further development and utilization of pestalotiopsone, xanthone, and chloroazaphilone derivatives as source of potential anti-IAV chemotherapy agents.

## Author contributions

XiaL: designed the experiments and performed the isolation and characterization of all the compounds and wrote the manuscript; JY and FC: performed the antiviral experiment; XiuL: performed the isolation and purification of the fungal strain; CC: contributed to isolation of the compounds; XZ: designed the research work and wrote the manuscript; SL and YL: contributed in project design and manuscript preparation. All authors reviewed the manuscript and approved for submission.

### Conflict of interest statement

The authors declare that the research was conducted in the absence of any commercial or financial relationships that could be construed as a potential conflict of interest.
