# Supplementary material for: Structurally Diverse Polyketides From the Mangrove-Derived Fungus Diaporthe sp. SCSIO 41011 With Their Anti-influenza A Virus Activities
Source: Front Chem. 2018 Jul 12;6:282. doi: 10.3389/fchem.2018.00282 (PMC6052247; doi:10.3389/fchem.2018.00282)
Supplement: Supplementary file 1 [file Presentation_1.PDF]

# Supporting Information

## Structurally diverse polyketides from the mangrove-derived fungus *Diaporthe* sp. SCSIO 41011 with their anti-influenza A virus activities

***Xiaowei Luo[1,3,#], Jie Yang[2,#], Feimin Chen[2], Xiuping Lin[1], Chunmei Chen[1,3], Xuefeng Zhou[1,3]*, Shuwen Liu[2,4,]*, Yonghong Liu[1,3,]****

[1]*CAS Key Laboratory of Tropical Marine Bio-resources and Ecology, Guangdong Key Laboratory of Marine Materia Medica, South China Sea Institute of Oceanology, Chinese Academy of Sciences, Guangzhou 510301, China,* [2] *Guangdong Provincial Key Laboratory of New Drug Screening, Guangzhou Key Laboratory of Drug Research for Emerging Virus Prevention and Treatment, School of Pharmaceutical Sciences, Southern Medical University, Guangzhou 510515, China,* [3]*University of Chinese Academy of Sciences, Beijing 100049, China,* [4]*State Key Laboratory of Organ Failure Research, Southern Medical University, Guangzhou 510515, China*

[#] These authors contributed equally.

* Corresponding Authors: Xuefeng Zhou (xfzhou@scsio.ac.cn), Shuwen Liu (liusw@smu.edu.cn) or Yonghong Liu (yonghongliu@scsio.ac.cn).

# Contents of Supporting Information


| No. | Contents | Page |
|---|---|---|
| 1 | **Figure S1**. $^{1}$H NMR spectrum of dothiorelone O (**1**) ($CD_3OD$, 500 MHz) | **4** |
| 2 | **Figure S2.** $^{13}$C NMR and DEPT spectra of dothiorelone O (**1**) ($CD_3OD$, 125 MHz) | **4-5** |
| 3 | **Figure S3.** HSQC spectrum of dothiorelone O (**1**) ($CD_3OD$) | **5** |
| 4 | **Figure S4.** HMBC spectrum of dothiorelone O (**1**) ($CD_3OD$) | **6** |
| 5 | **Figure S5.** $^{1}$H-$^{1}$H COSY spectrum of dothiorelone O (**1**) ($CD_3OD$) | **6** |
| 6 | **Figure S6**. $^{1}$H NMR spectrum of dothiorelone O (**1**) ($CDCl_3$, 500 MHz) | **7** |
| 7 | **Figure S7.** $^{13}$C NMR and DEPT spectra of dothiorelone O (**1**) ($CDCl_3$, 125 MHz) | **7-8** |
| 8 | **Figure S8.** HSQC spectrum of dothiorelone O (**1**) ($CDCl_3$) | **8** |
| 9 | **Figure S9.** HMBC spectrum of dothiorelone O (**1**) ($CDCl_3$) | **9** |
| 10 | **Figure S10.** $^{1}$H-$^{1}$H COSY spectrum of dothiorelone O (**1**) ($CDCl_3$) | **9** |
| 11 | **Figure S11.** Negative HR-ESI-MS spectrum of dothiorelone O (**1**) | **10** |
| 12 | **Figure S12.** UV spectrum of dothiorelone O (**1**) | **10** |
| 13 | **Figure S13.** IR spectrum of dothiorelone O (**1**) | **11** |
| 14 | **Figure S14**. $^{1}$H NMR spectrum of (15*R*)-acetoxydothiorelone A (**2**) ($CD_3OD$, 700 MHz) | **11** |
| 15 | **Figure S15.** $^{13}$C NMR and DEPT spectra of (15*R*)-acetoxydothiorelone A (**2**) ($CD_3OD$, 175 MHz) | **12** |
| 16 | **Figure S16.** HSQC spectrum of (15*R*)-acetoxydothiorelone A (**2**) ($CD_3OD$) | **13** |
| 17 | **Figure S17.** HMBC spectrum of (15*R*)-acetoxydothiorelone A (**2**) ($CD_3OD$) | **13** |
| 18 | **Figure S18.** $^{1}$H-$^{1}$H COSY spectrum of (15*R*)-acetoxydothiorelone A (**2**) ($CD_3OD$) | **14** |
| 19 | **Figure S19**. $^{1}$H NMR spectrum of (15*R*)-acetoxydothiorelone A (**2**) ($CDCl_3$, 700 MHz) | **14** |
| 20 | **Figure S20.** $^{13}$C NMR and DEPT spectra of (15*R*)-acetoxydothiorelone A (**2**) ($CDCl_3$, 175 MHz) | **15** |
| 21 | **Figure S21.** HSQC spectrum of (15*R*)-acetoxydothiorelone A (**2**) ($CDCl_3$) | **16** |
| 22 | **Figure S22.** HMBC spectrum of (15*R*)-acetoxydothiorelone A (**2**) ($CDCl_3$) | **16** |
| 23 | **Figure S23.** $^{1}$H-$^{1}$H COSY spectrum of (15*R*)-acetoxydothiorelone A (**2**) ($CDCl_3$) | **17** |
| 24 | **Figure S24.** HR-ESI-MS spectrum of (15*R*)-acetoxydothiorelone A (**2**) | **17** |
| 25 | **Figure S25.** UV spectrum of (15*R*)-acetoxydothiorelone A (**2**) | **18** |
| 26 | **Figure S26.** IR spectrum of (15*R*)-acetoxydothiorelone A (**2**) | **18** |
| 27 | **Figure S27**. $^{1}$H NMR spectrum of pestalotiopsone H (**13**) ($CD_3OD$, 700 MHz) | **19** |
| 28 | **Figure S28** $^{13}$C NMR and DEPT spectra of pestalotiopsone H (**13**) ($CD_3OD$, 175 MHz) | **19-20** |
| 29 | **Figure S29.** HSQC spectrum of pestalotiopsone H (**13**) ($CD_3OD$) | **20** |
| 30 | **Figure S30.** HMBC spectrum of pestalotiopsone H (**13**) ($CD_3OD$) | **21** |
| 31 | **Figure S31.** $^{1}$H-$^{1}$H COSY spectrum of pestalotiopsone H (**13**) ($CD_3OD$) | **21** |
| 32 | **Figure S32.** Positive and negative LR-ESI-MS spectra of pestalotiopsone H (**13**) | **22** |
| 33 | **Figure S33.** HR-ESI-MS spectrum of pestalotiopsone H (**13**) | **23** |
| 34 | **Figure S34.** UV spectrum of pestalotiopsone H (**13**) | **23** |

| | | |
|---|---|---|
| 35 | **Figure S35.** IR spectrum of pestalotiopsone H (**13**) | 24 |
| 36 | **Figure S36**. $^1$H NMR spectrum of (±)-microsphaerophthalide H (**17**) ($CD_3OD$, 700 MHz) | 24 |
| 37 | **Figure S37.** $^{13}$C NMR and DEPT spectra of (±)-microsphaerophthalide H (**17**) ($CD_3OD$, 175 MHz) | 25 |
| 38 | **Figure S38.** HSQC spectrum of (±)-microsphaerophthalide H (**17**) ($CD_3OD$) | 26 |
| 39 | **Figure S39.** HMBC spectrum of (±)-microsphaerophthalide H (**17**) ($CD_3OD$) | 26 |
| 40 | **Figure S40.** HR-ESI-MS spectrum of (±)-microsphaerophthalide H (**17**) | 27 |
| 41 | **Figure S41.** UV spectrum of (±)-microsphaerophthalide H (**17**) | 27 |
| 42 | **Figure S42.** IR spectrum of (±)-microsphaerophthalide H (**17**) | 28 |
| 43 | **Figure S43**. $^1$H NMR spectrum of microsphaerophthalide I (**18**) (DMSO-$d_6$, 700 MHz) | 28 |
| 44 | **Figure S44.** $^{13}$C NMR and DEPT spectra of microsphaerophthalide I (**18**) (DMSO-$d_6$, 175 MHz) | 29 |
| 45 | **Figure S45.** HSQC spectrum of microsphaerophthalide I (**18**) (DMSO-$d_6$) | 30 |
| 46 | **Figure S46.** HMBC spectrum of microsphaerophthalide I (**18**) (DMSO-$d_6$) | 30 |
| 47 | **Figure S47.** HR-ESI-MS spectrum of microsphaerophthalide I (**18**) | 31 |
| 48 | **Figure S48.** UV spectrum of microsphaerophthalide I (**18**) | 31 |
| 49 | **Figure S49.** IR spectrum of microsphaerophthalide I (**18**) | 32 |
| 50 | **Figure S50**. $^1$H NMR spectrum of methyl convolvulopyrone (**21**) ($CD_3OD$, 700 MHz) | 32 |
| 51 | **Figure S51.** $^{13}$C NMR and DEPT spectra of methyl convolvulopyrone (**21**) ($CD_3OD$, 175 MHz) | 33 |
| 52 | **Figure S52.** HSQC spectrum of methyl convolvulopyrone (**21**) ($CD_3OD$) | 34 |
| 53 | **Figure S53.** HMBC spectrum of methyl convolvulopyrone (**21**) ($CD_3OD$) | 34 |
| 54 | **Figure S54.** NOESY spectrum of methyl convolvulopyrone (**21**) ($CD_3OD$) | 35 |
| 55 | **Figure S55.** HR-ESI-MS spectrum of methyl convolvulopyrone (**21**) | 35 |
| 56 | **Figure S56.** UV spectrum of methyl convolvulopyrone (**21**) | 36 |
| 57 | **Figure S57.** IR spectrum of methyl convolvulopyrone (**21**) | 37 |
| 58 | **Figure S58.** Chiral HPLC analysis of **17** (A, dissolved in MeOH) and **18** (B, DMSO) using CHIRALPAK IC column (250 × 4.6 mm, 5 $\mu$m). | 37-38 |
| 59 | **Table S1.** Energies of **18** at MMFF94 force field. | 38 |
| 60 | **Table S2.** Energies of **18** at B3LYP/6–31+g(d, p) level in methanol. | 39 |
| 61 | **Figure S59.** The optimized conformers and equilibrium populations of microsphaerophthalide I (**18**) | 39 |
| 62 | The physicochemical data of the known compounds **7**–**12**. | 39-45 |
| 63 | References | 45 |

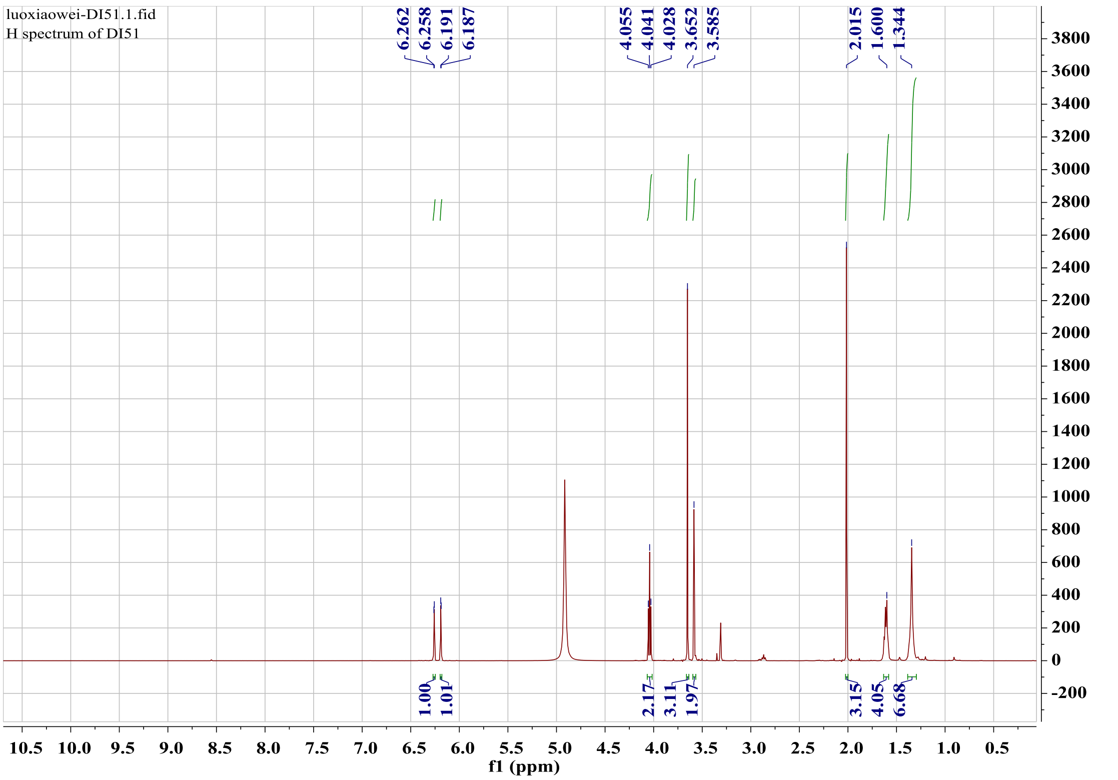


**Figure S1**. ¹H NMR spectrum of dothiorelone O (**1**) ($CD_3OD$, 500 MHz)

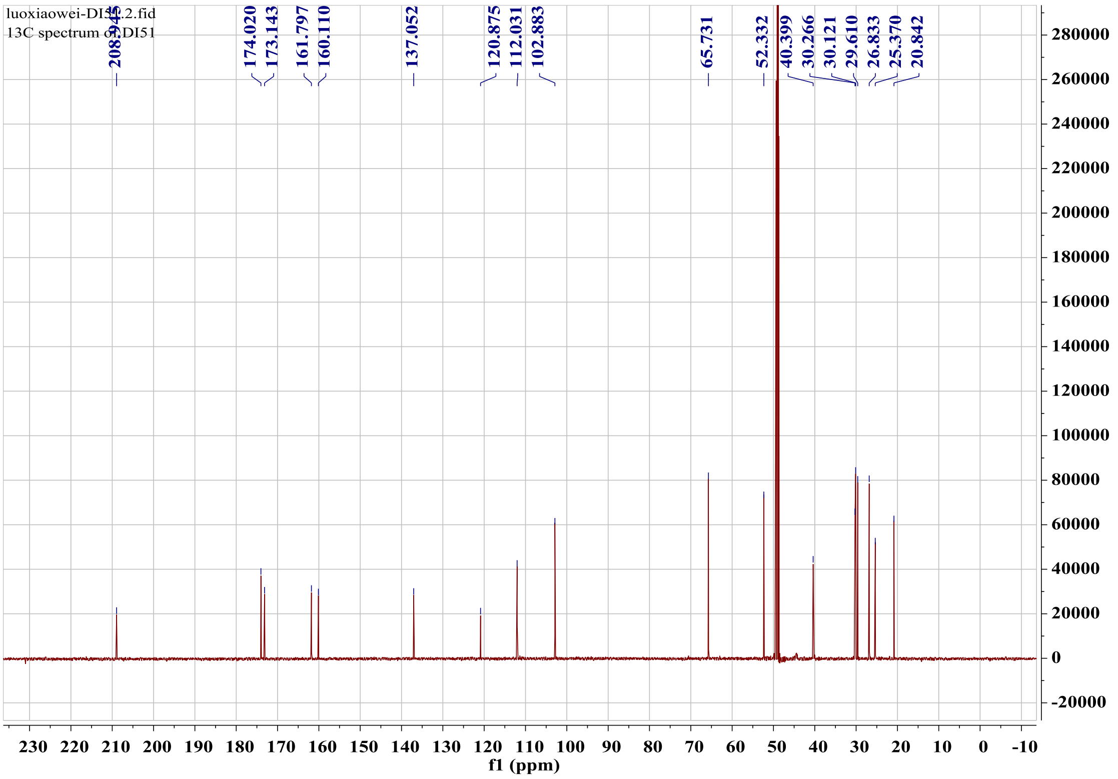

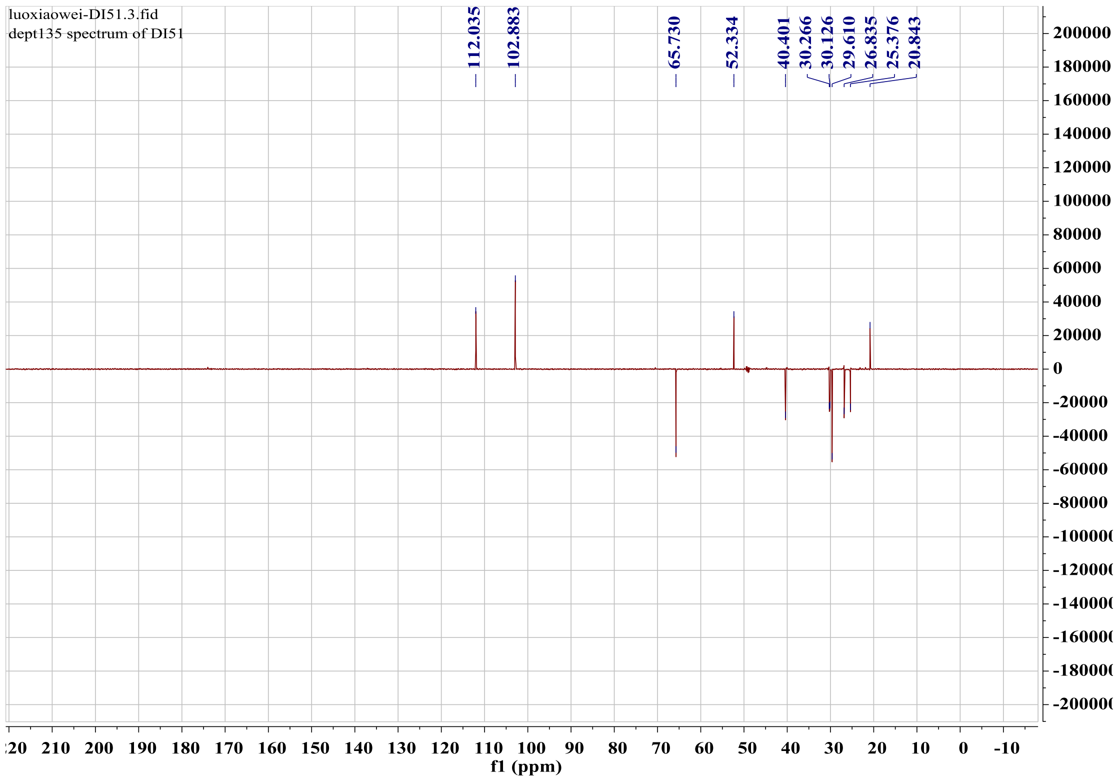


**Figure S2.** $^{13}$C NMR and DEPT spectra of dothiorelone O (**1**) ($CD_3OD$, 125 MHz)

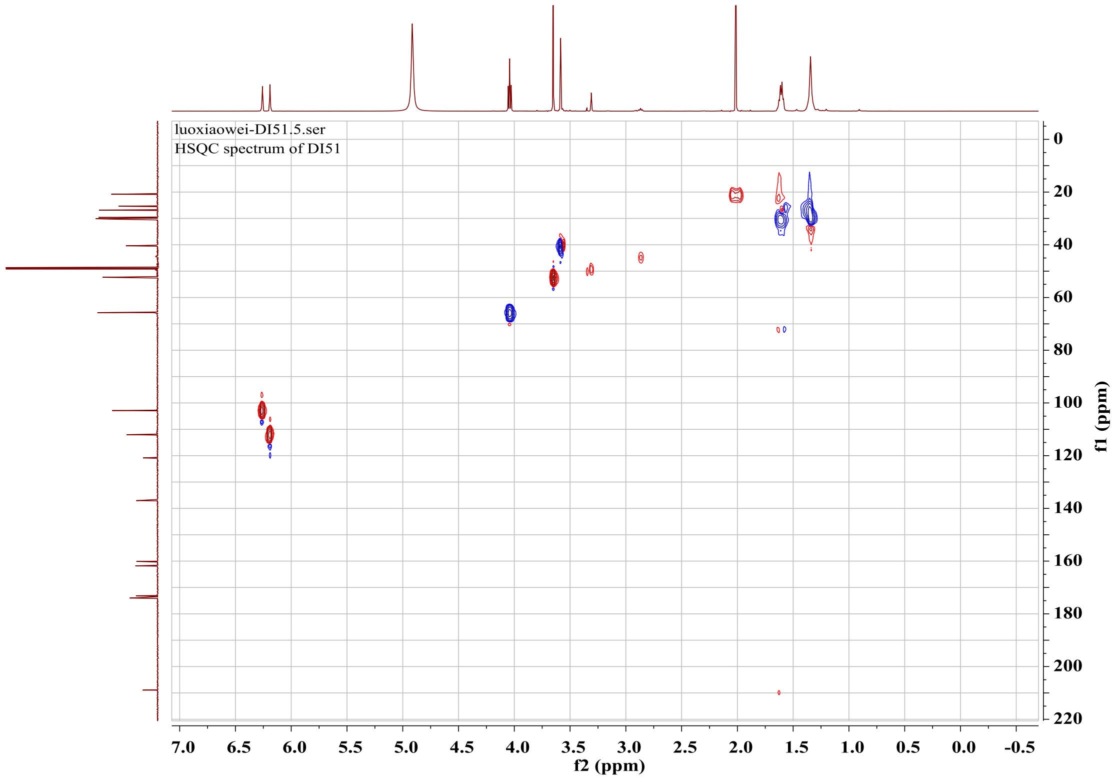


**Figure S3.** HSQC spectrum of dothiorelone O (**1**) ($CD_3OD$)

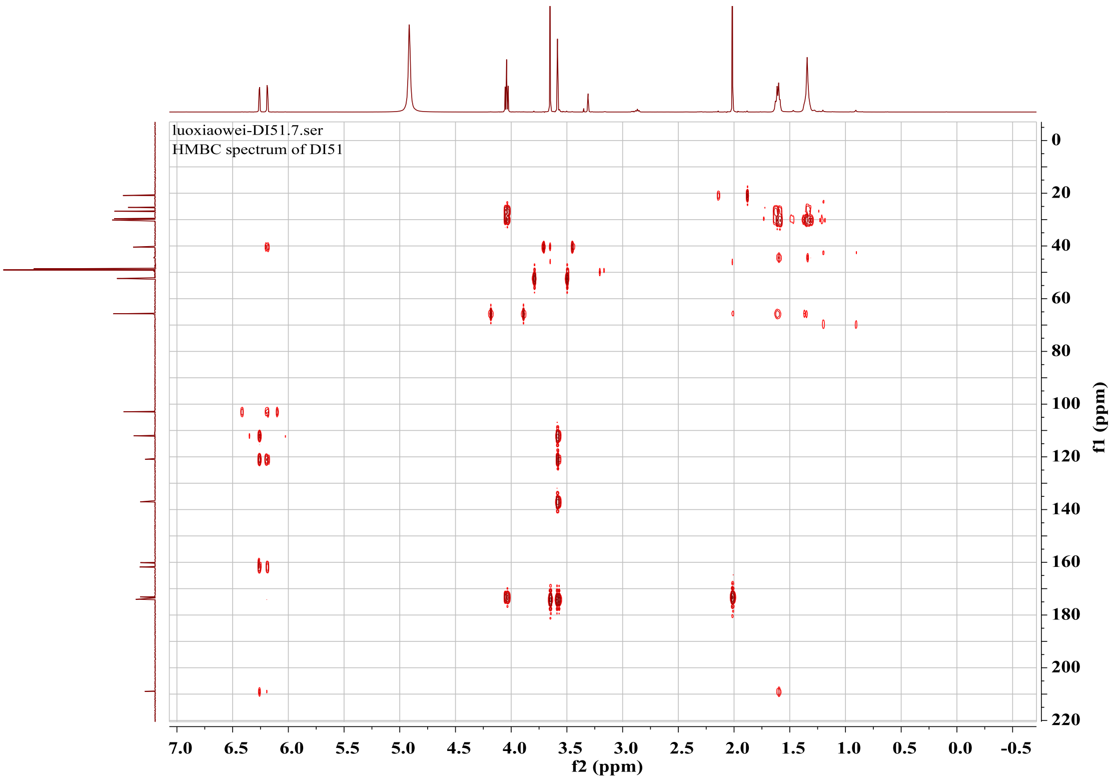


**Figure S4.** HMBC spectrum of dothiorelone O (**1**) ($CD_3OD$)

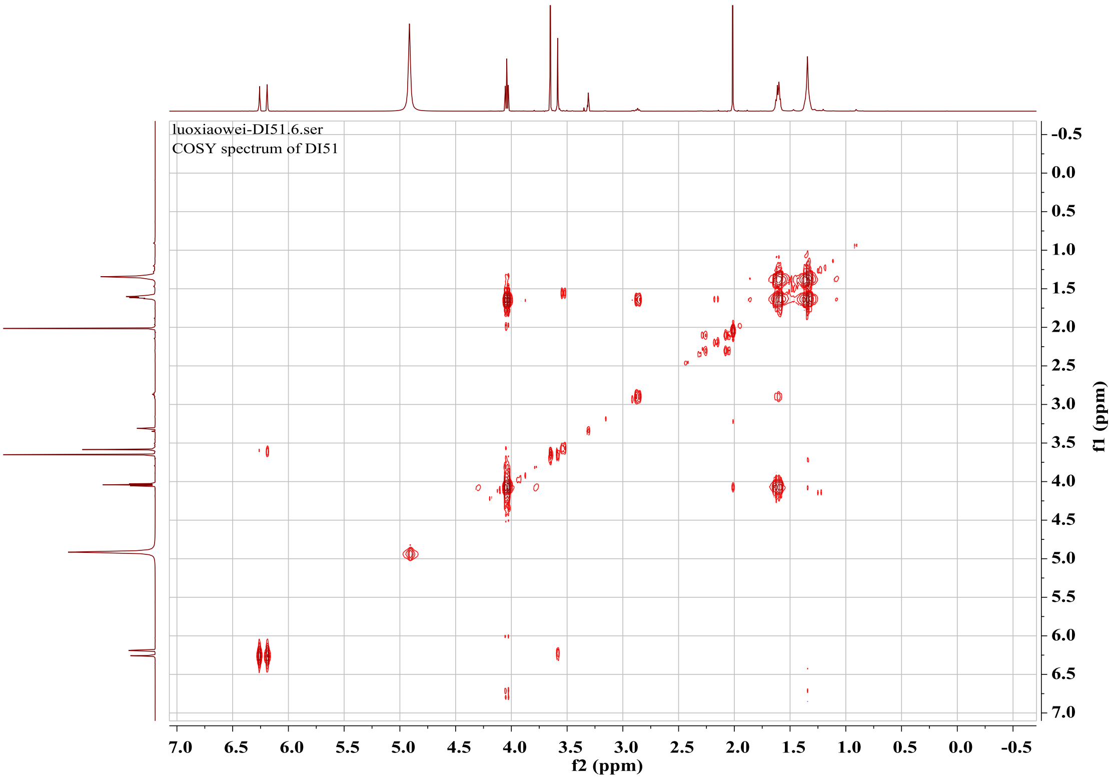


**Figure S5.** $^1H$-$^1H$ COSY spectrum of dothiorelone O (**1**) ($CD_3OD$)

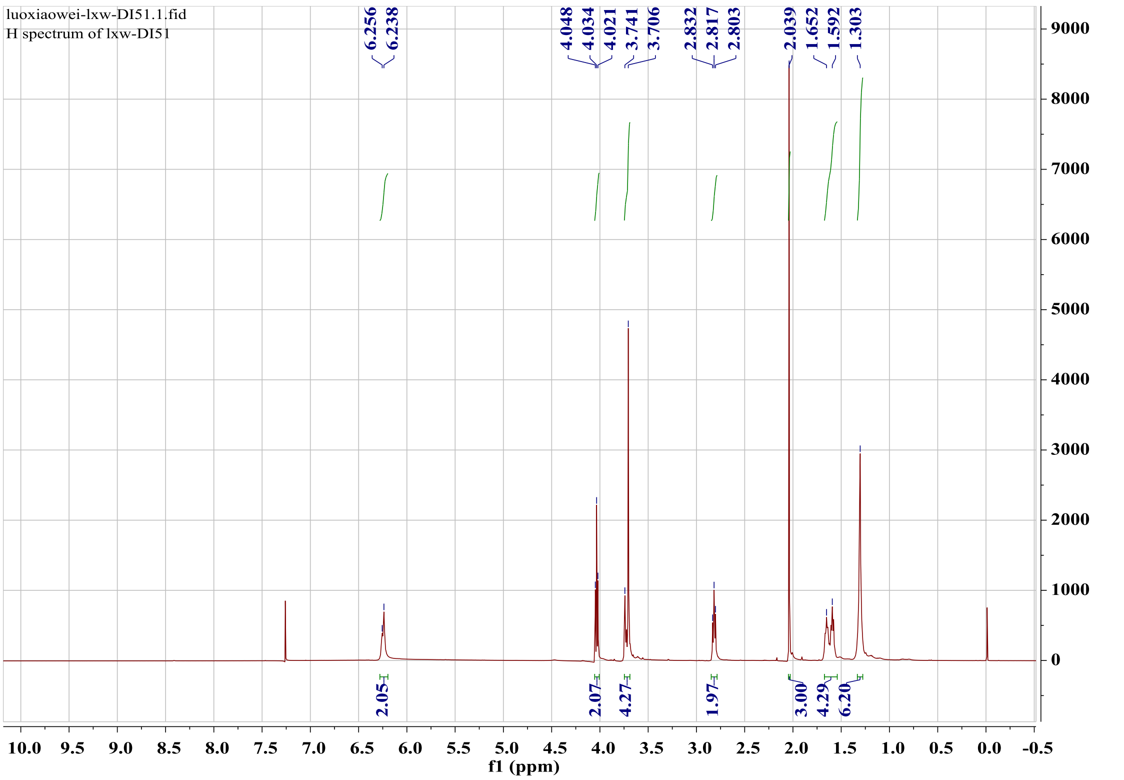


**Figure S6**. $^{1}$H NMR spectrum of dothiorelone O (**1**) ($CDCl_3$, 500 MHz)

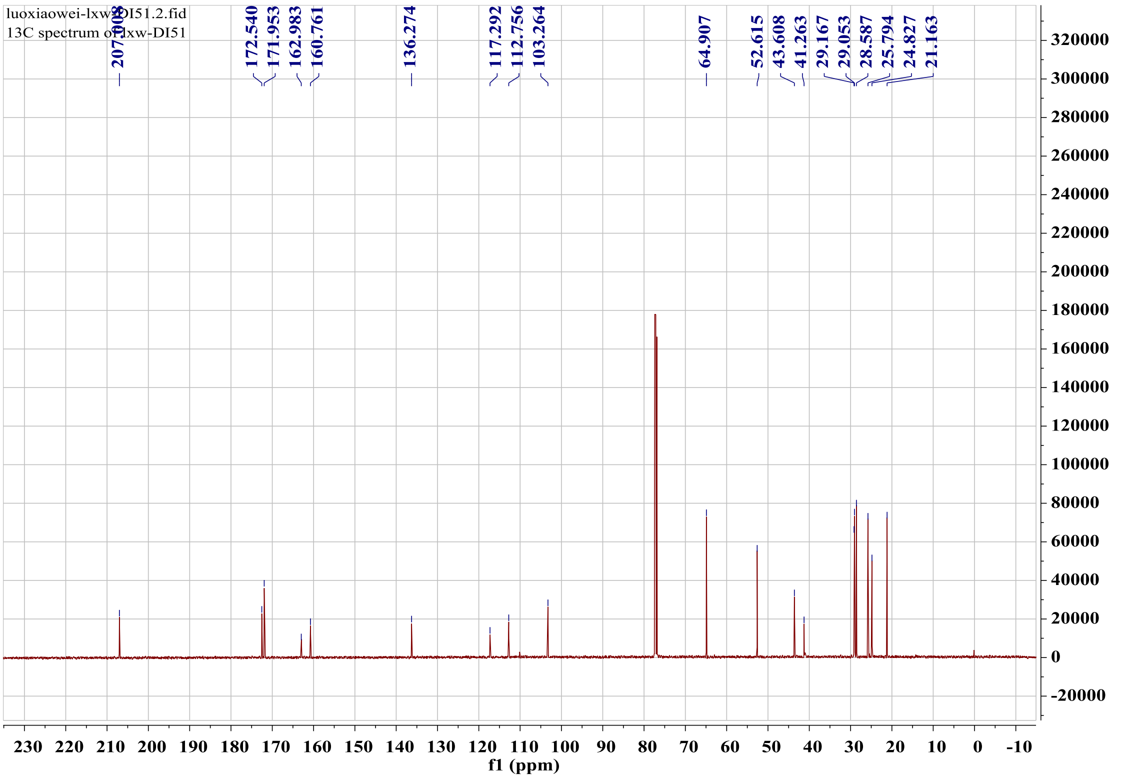

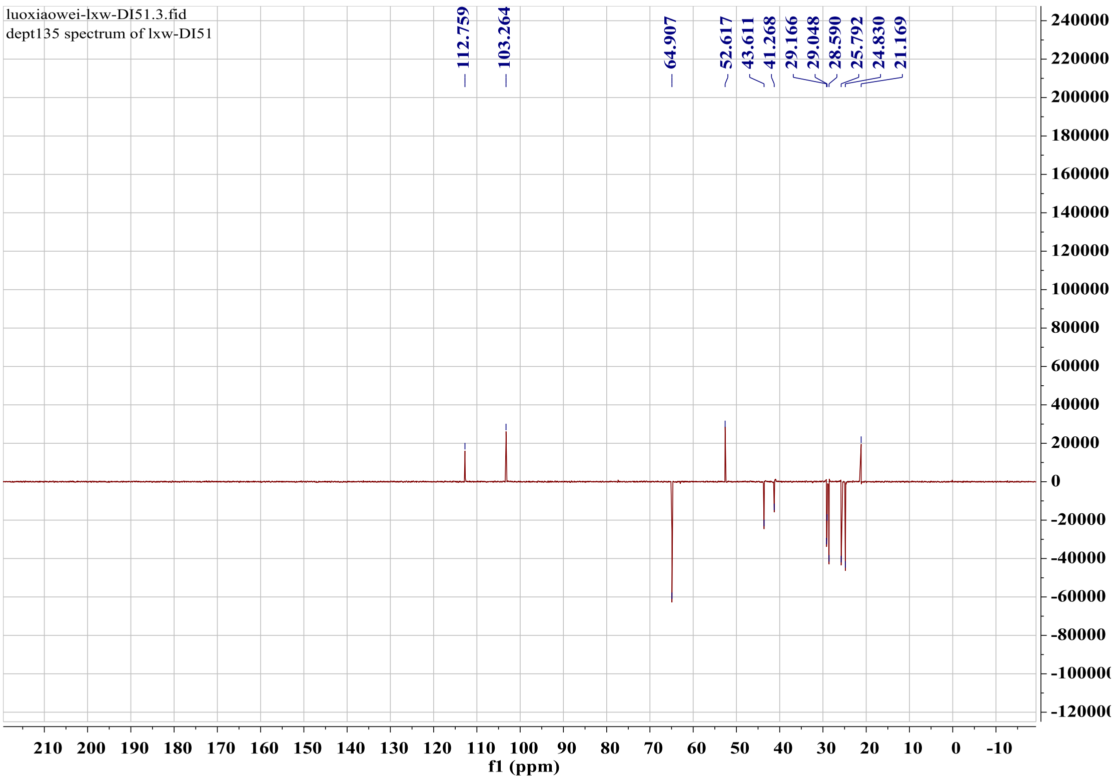


**Figure S7.** $^{13}$C NMR and DEPT spectra of dothiorelone O (**1**) ($CDCl_3$, 125 MHz)

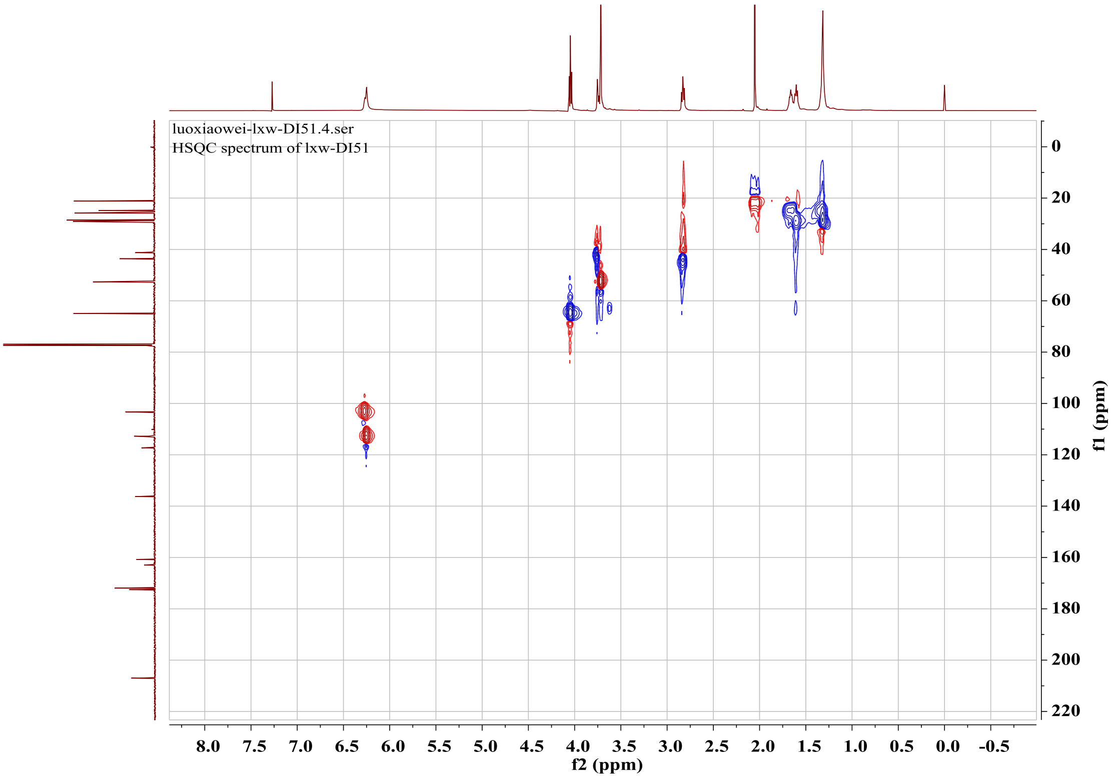


**Figure S8.** HSQC spectrum of dothiorelone O (**1**) ($CDCl_3$)

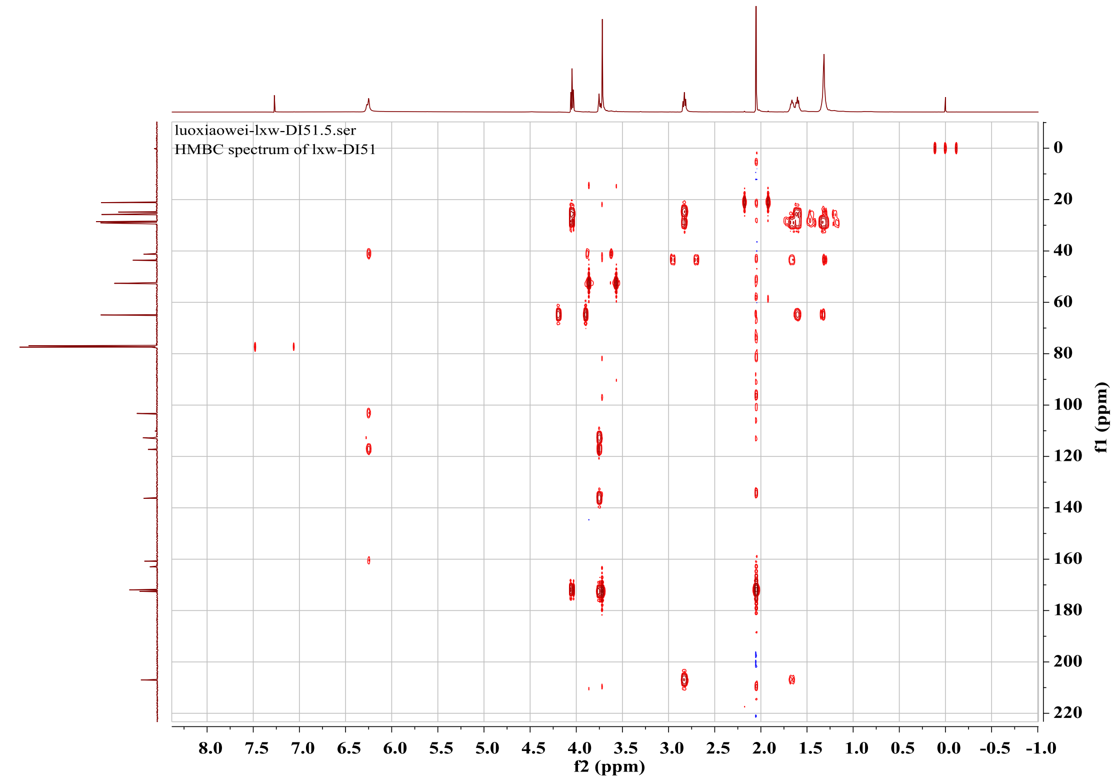


**Figure S9.** HMBC spectrum of dothiorelone O (**1**) ($CDCl_3$)

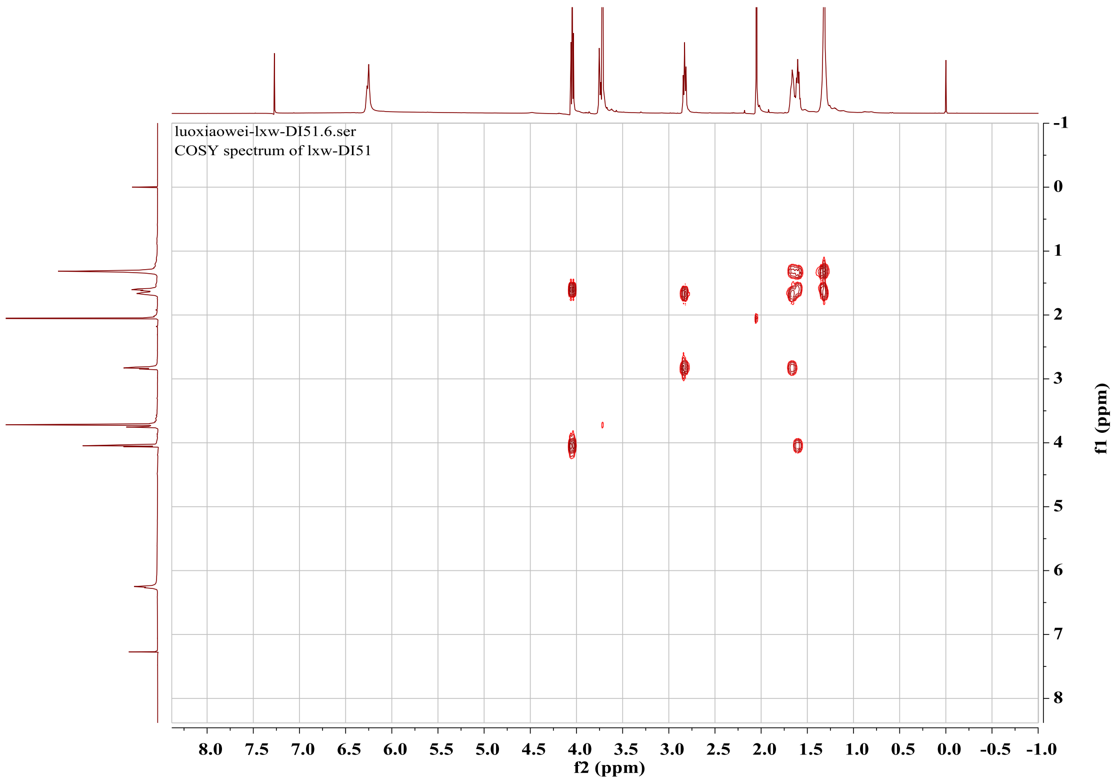


**Figure S10.** $^1H$-$^1H$ COSY spectrum of dothiorelone O (**1**) ($CDCl_3$)

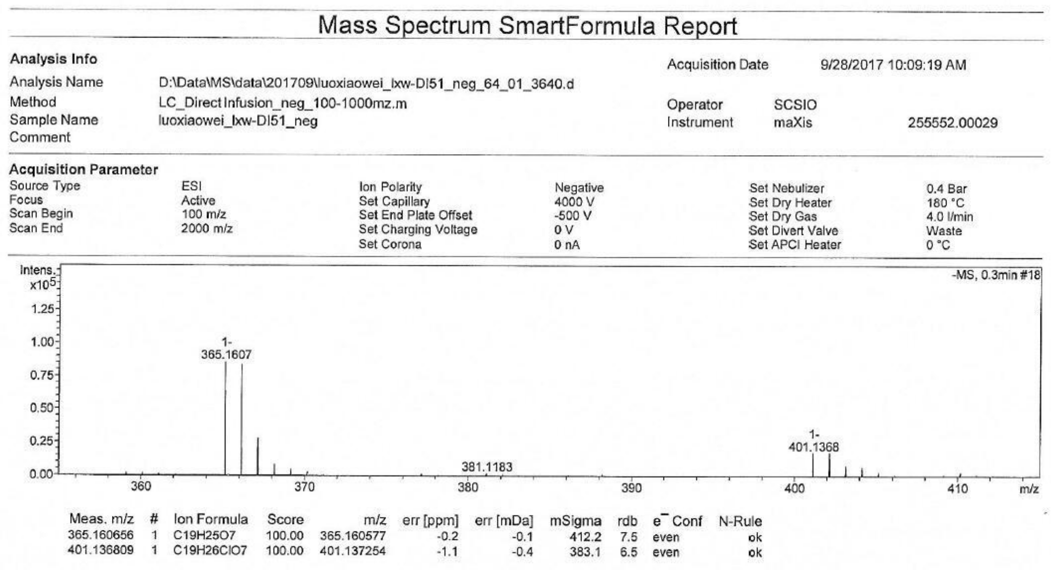


**Figure S11.** Negative HR-ESI-MS spectrum of dothiorelone O (**1**)

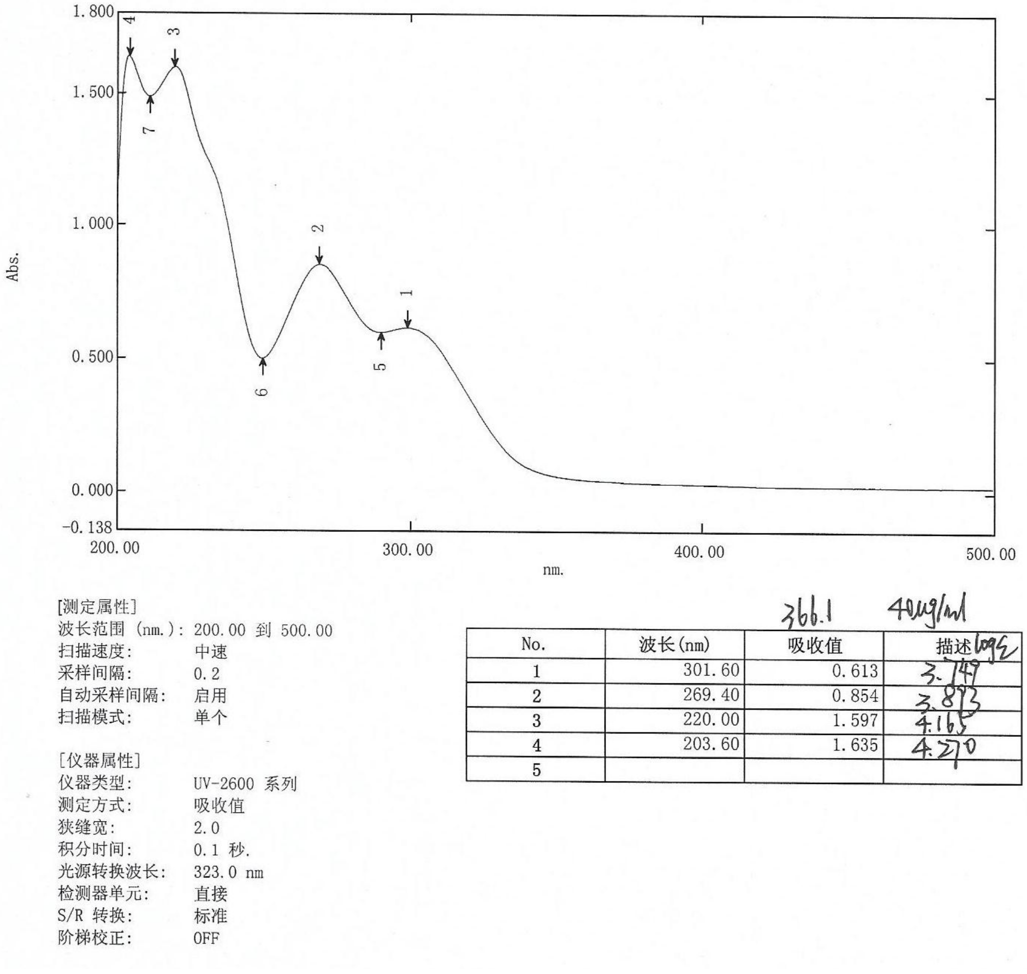


**Figure S12.** UV spectrum of dothiorelone O (**1**)

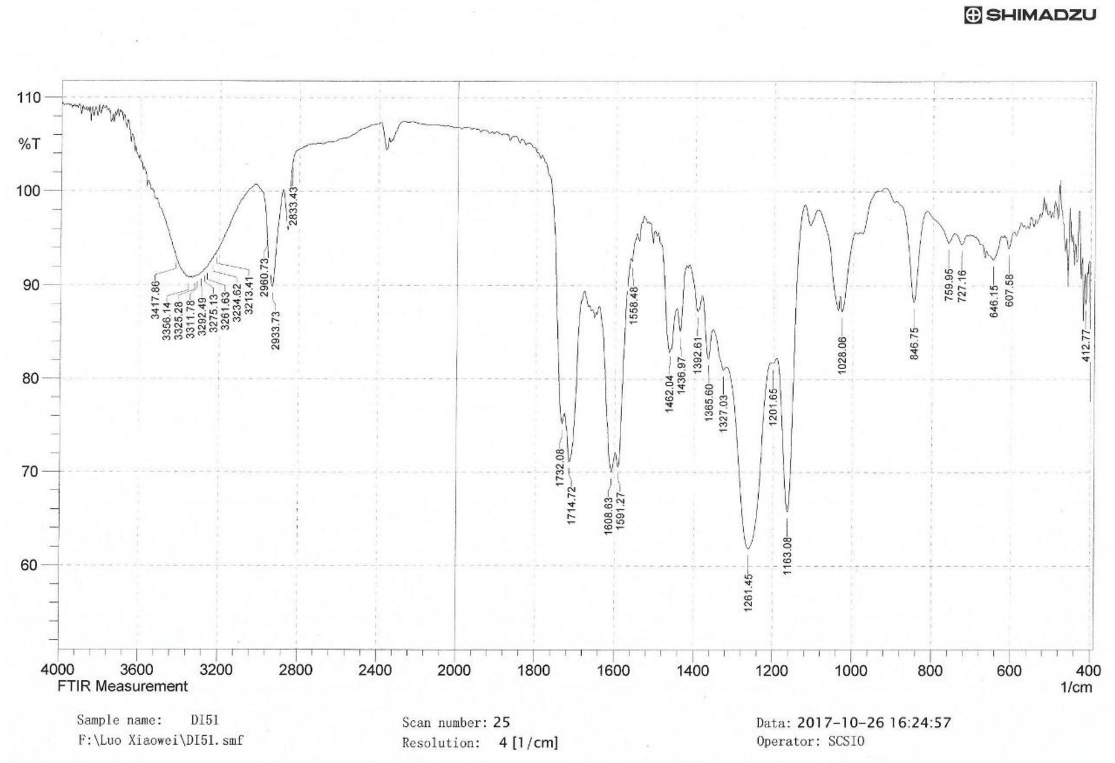

**Figure S13.** IR spectrum of dothiorelone O (**1**)

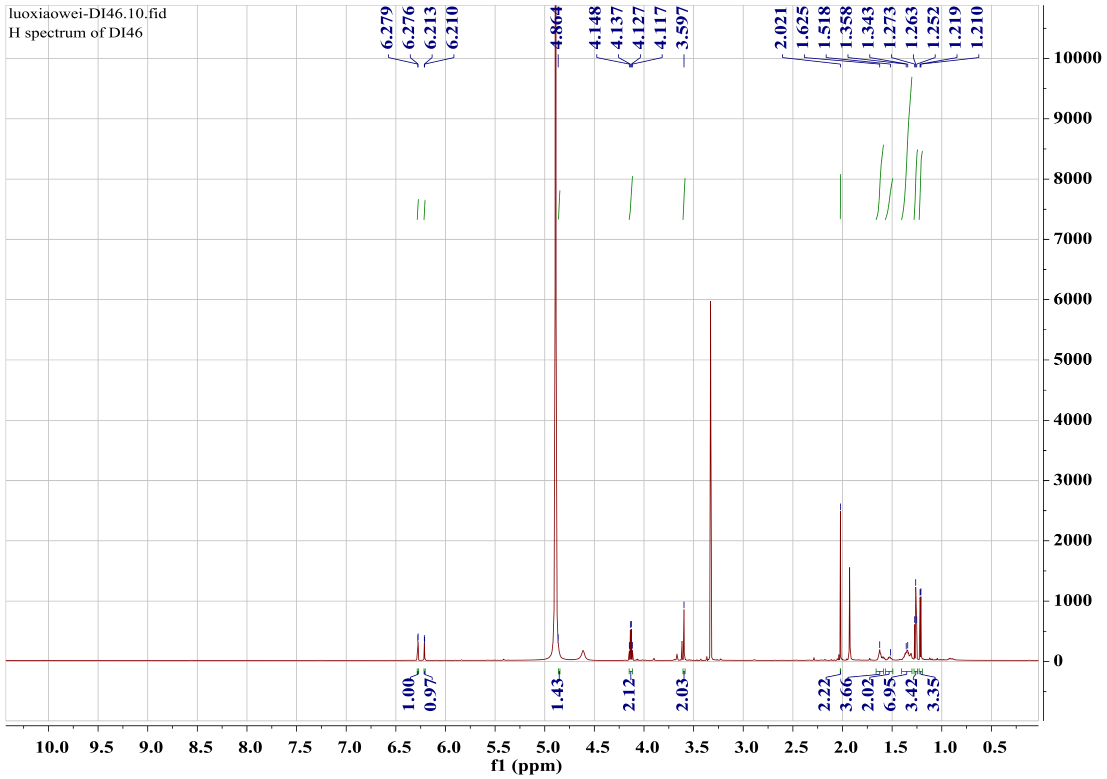

**Figure S14**. $^{1}$H NMR spectrum of (15*R*)-acetoxydothiorelone A (**2**) ($CD_3OD$, 700 MHz)

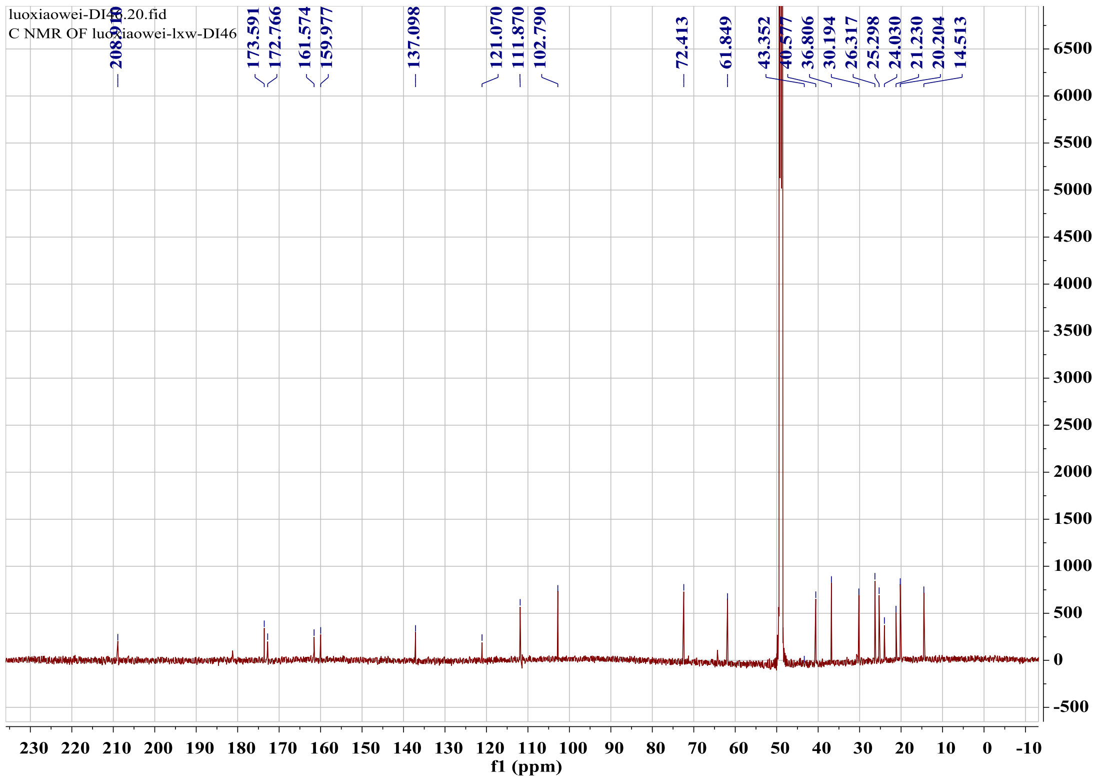


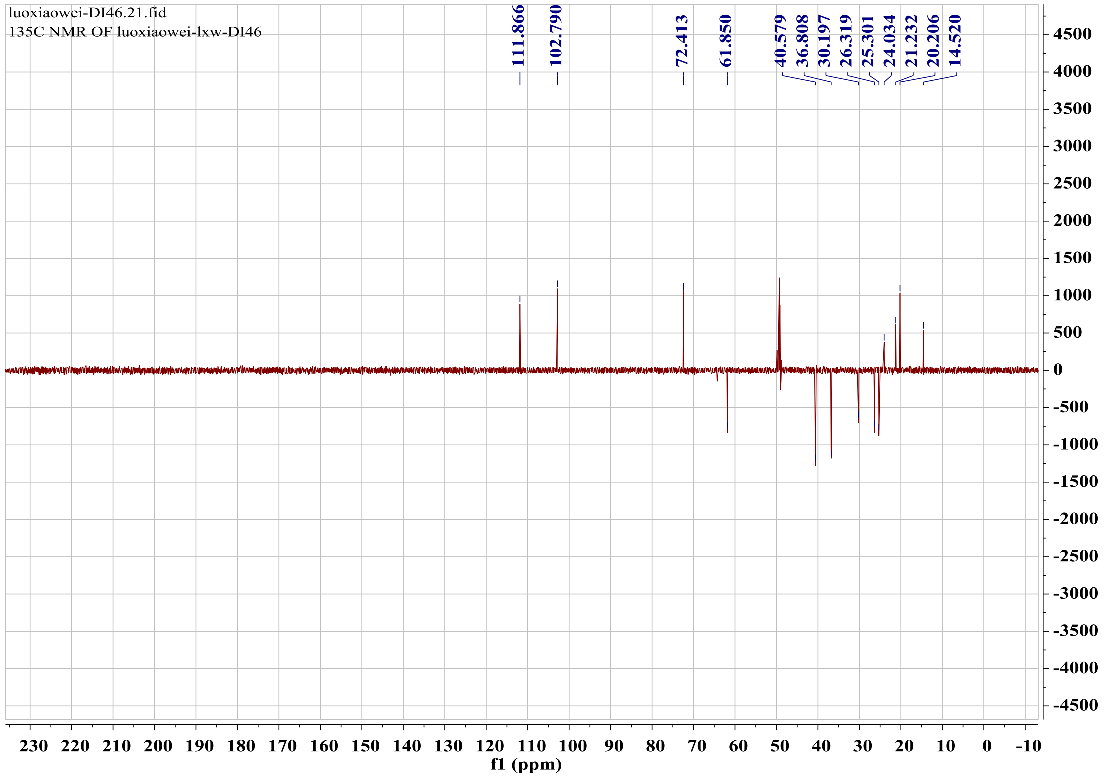


**Figure S15.** $^{13}$C NMR and DEPT spectra of (15*R*)-acetoxydothiorelone A (**2**) ($CD_3OD$, 175 MHz)

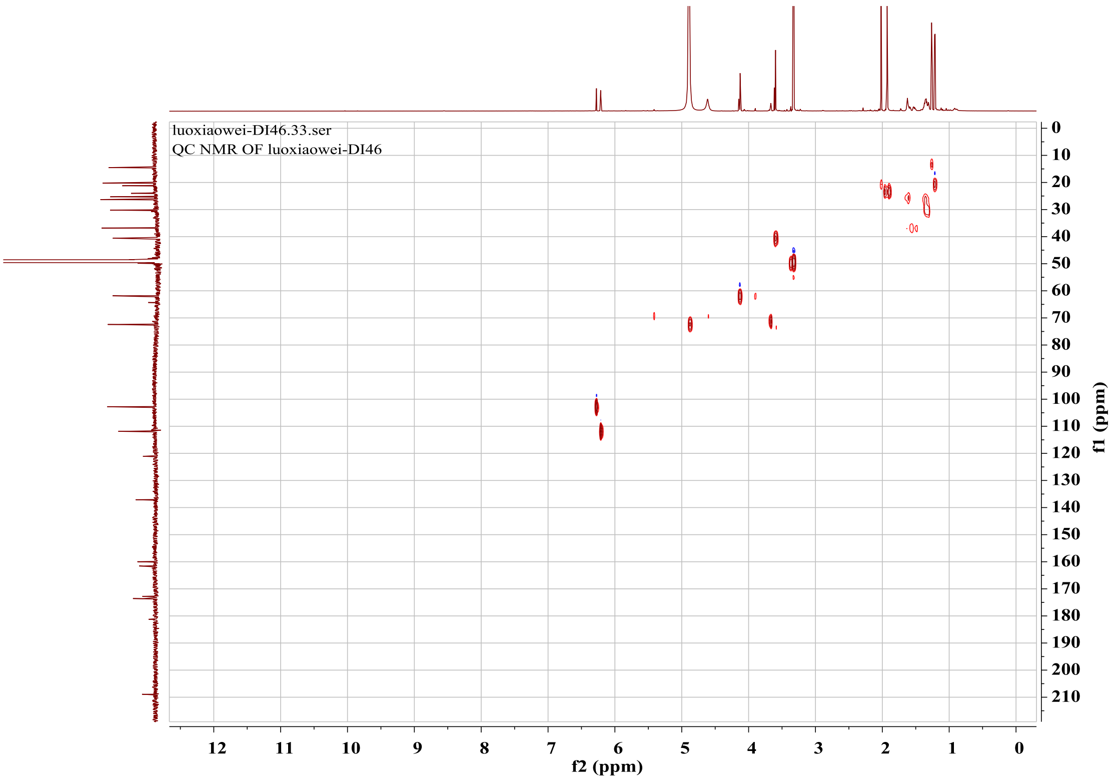


**Figure S16.** HSQC spectrum of (15*R*)-acetoxydothiorelone A (**2**) ($CD_3OD$)

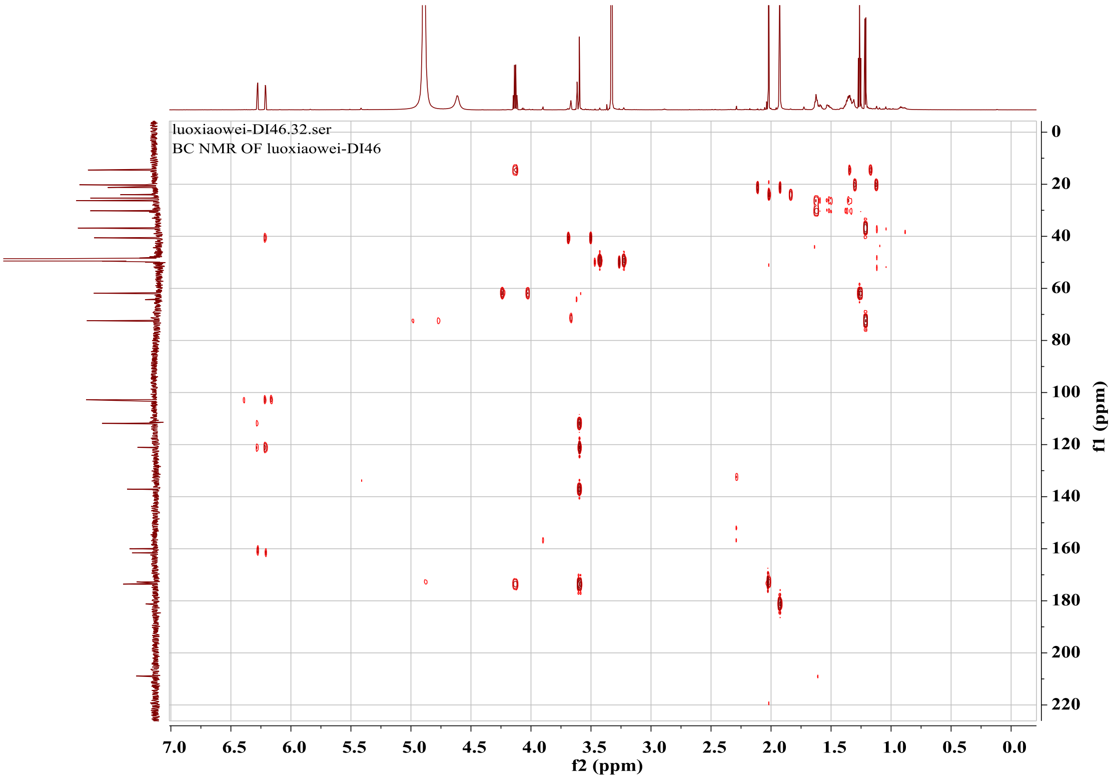


**Figure S17.** HMBC spectrum of (15*R*)-acetoxydothiorelone A (**2**) ($CD_3OD$)

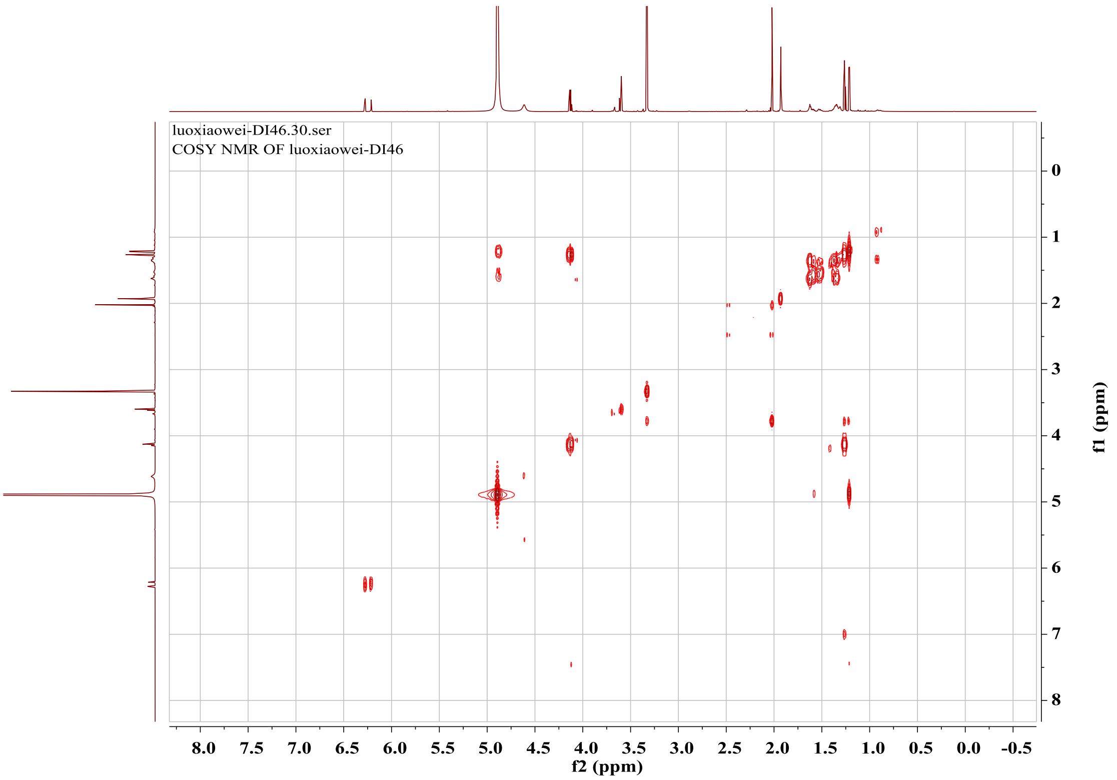


**Figure S18.** $^{1}$H-$^{1}$H COSY spectrum of (15*R*)-acetoxydothiorelone A (**2**) ($CD_3OD$)

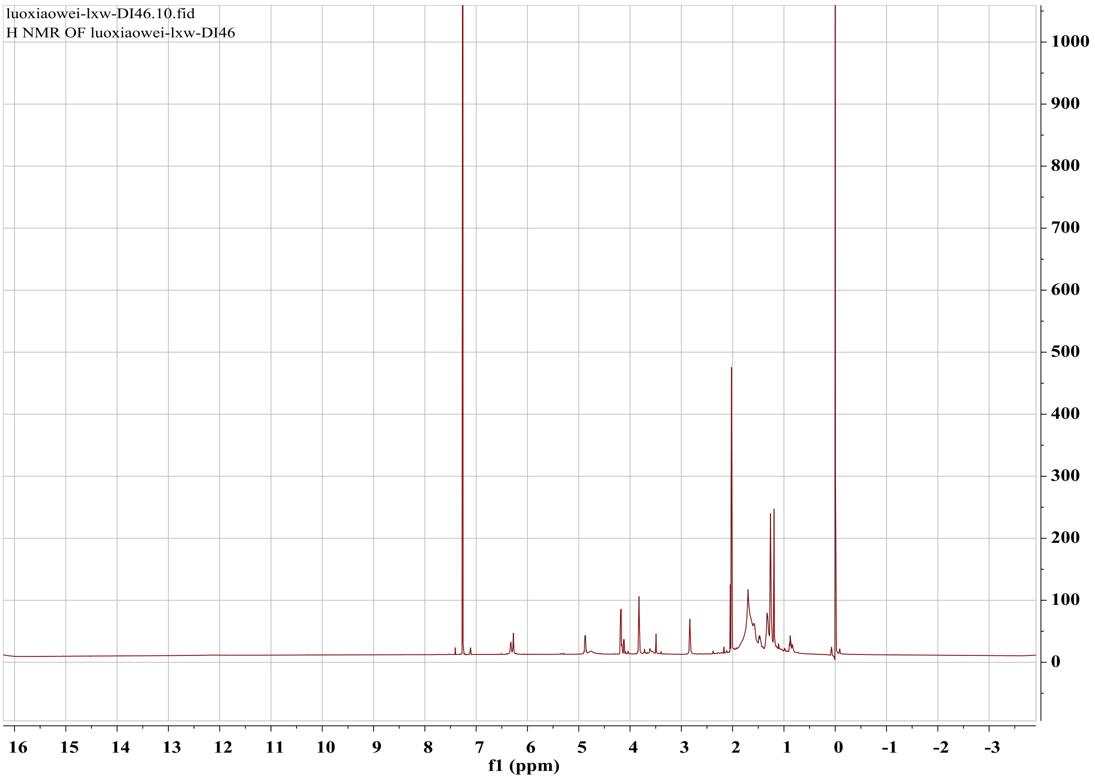


**Figure S19**. $^{1}$H NMR spectrum of (15*R*)-acetoxydothiorelone A (**2**) ($CDCl_3$, 700 MHz)

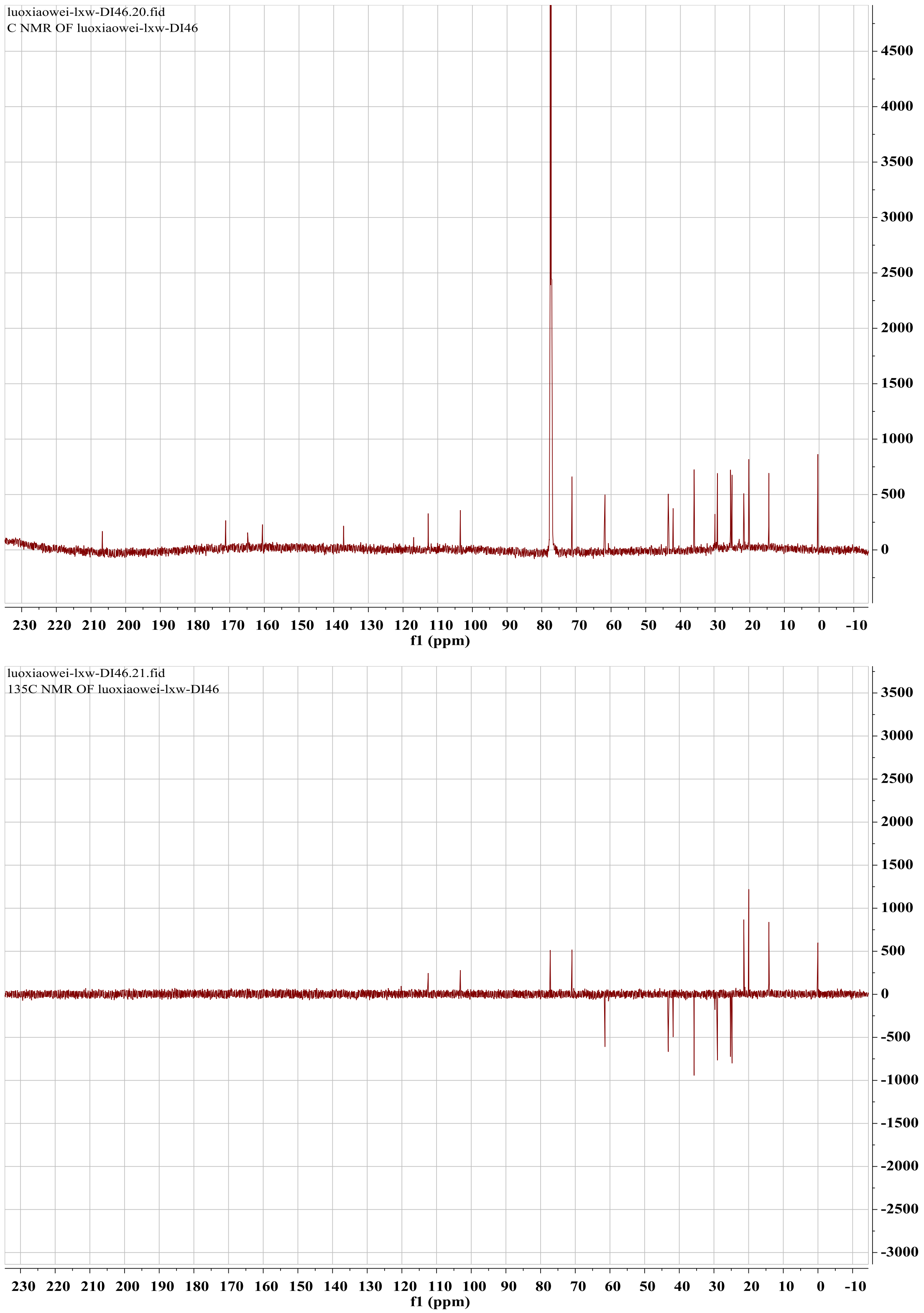


**Figure S20.** $^{13}$C NMR and DEPT spectra of (15*R*)-acetoxydothiorelone A (**2**) ($CDCl_3$, 175 MHz)

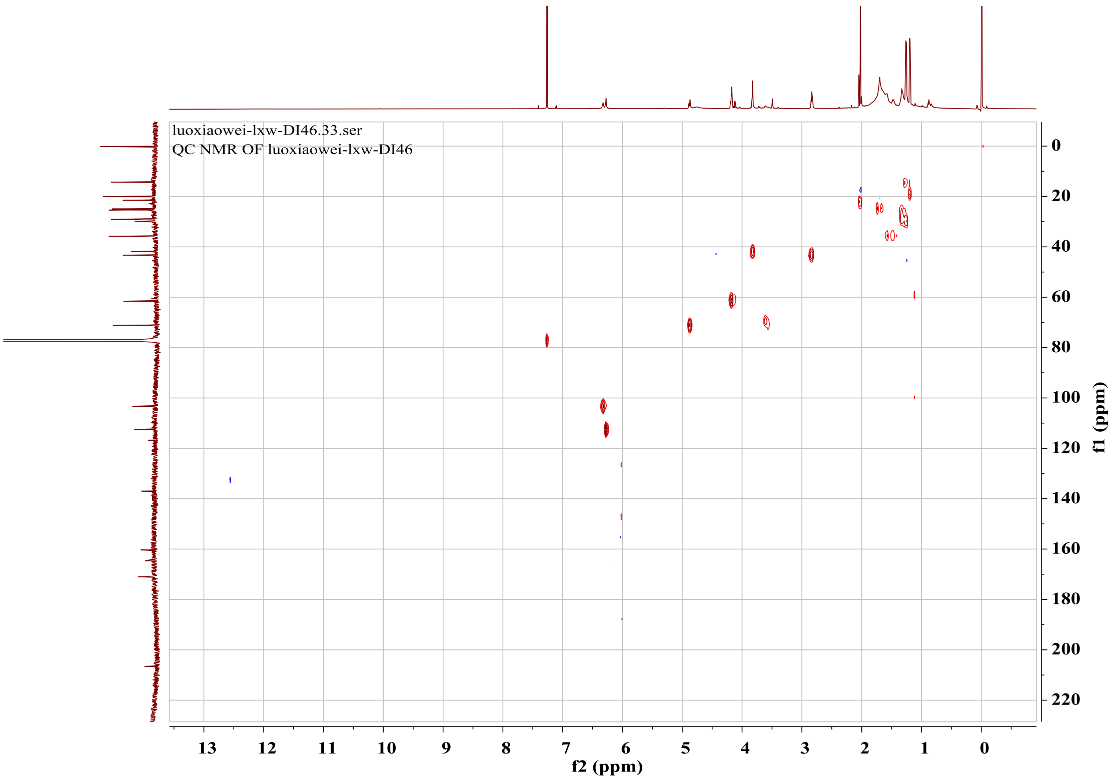


**Figure S21.** HSQC spectrum of (15*R*)-acetoxydothiorelone A (**2**) ($CDCl_3$)

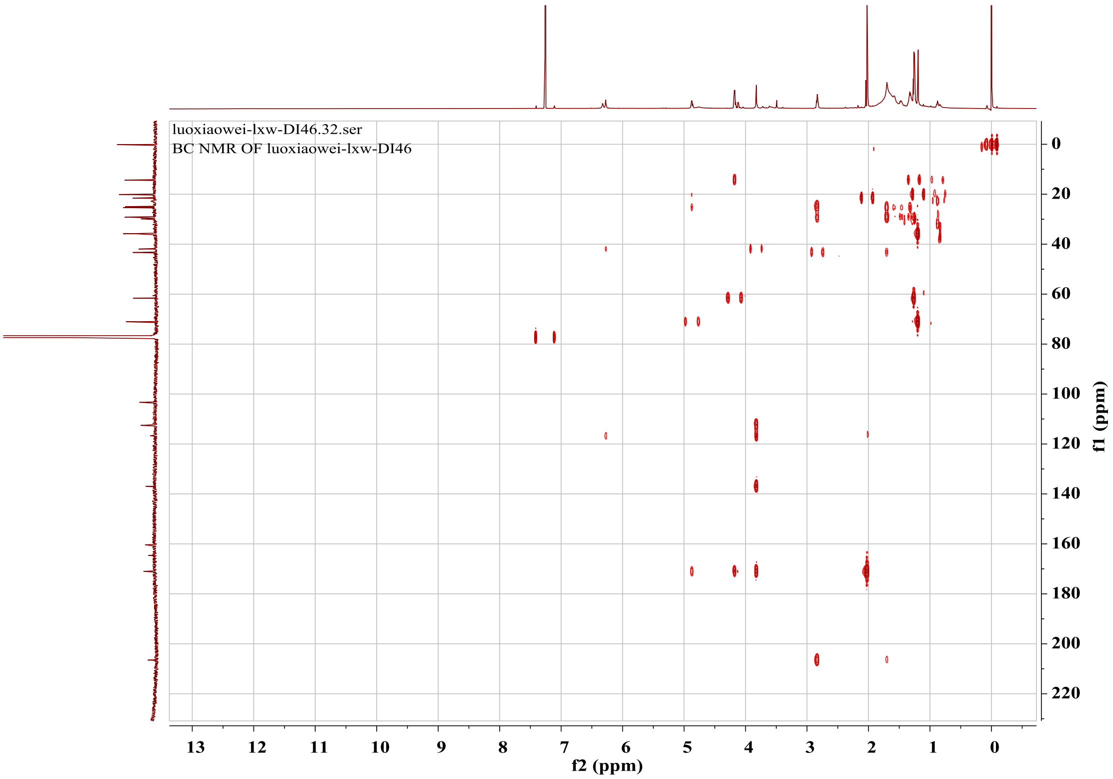


**Figure S22.** HMBC spectrum of (15*R*)-acetoxydothiorelone A (**2**) ($CDCl_3$)

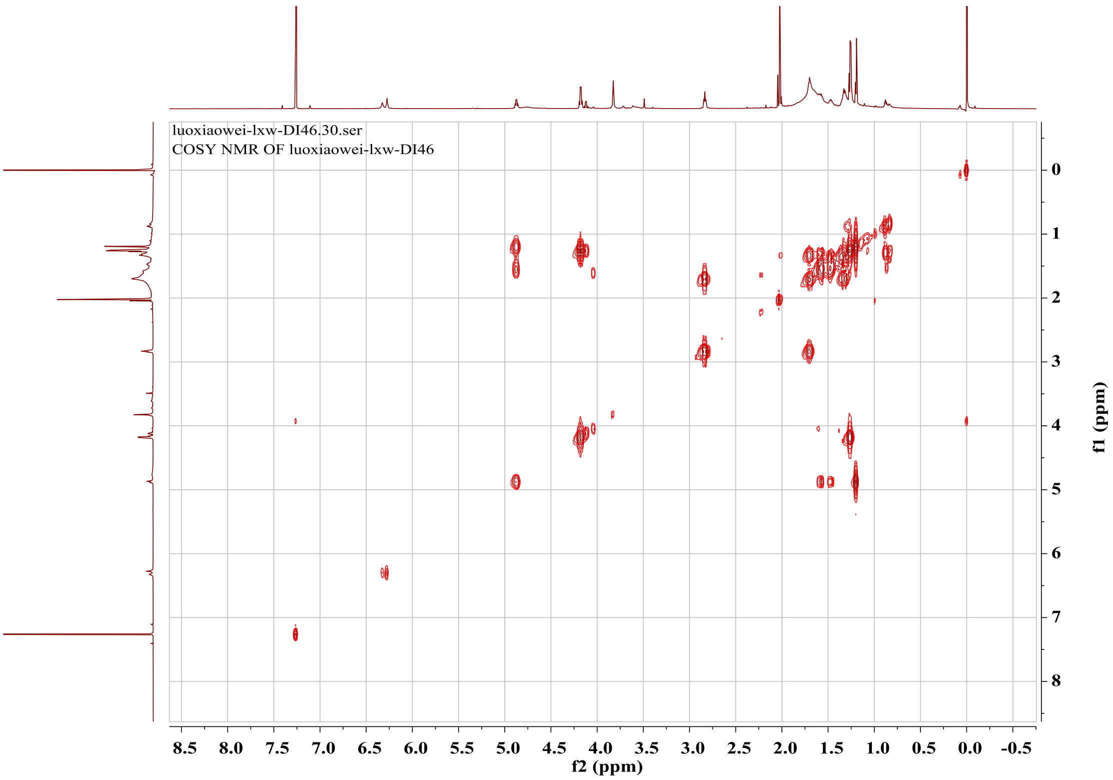


**Figure S23.** $^{1}H$-$^{1}H$ COSY spectrum of (15*R*)-acetoxydothiorelone A (**2**) ($CDCl_3$)

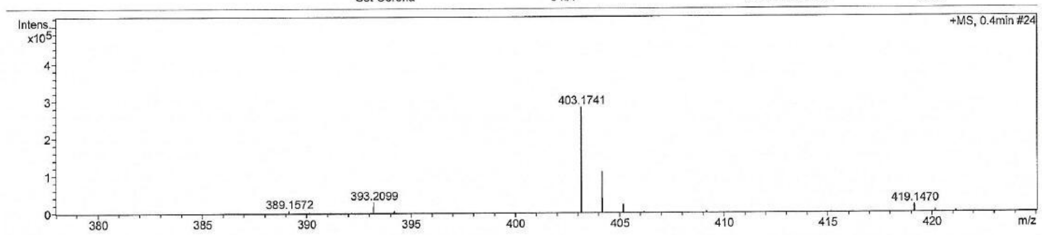

Mass Spectrum SmartFormula Report

**Analysis Info**

| | | | |
|---|---|---|---|
| Analysis Name | D:\Data\MS\data\201710\luoxiaowei_lxw-DI46_pos_81_01_3651.d | Acquisition Date | 10/9/2017 4:49:24 PM |
| Method | LC_Direct Infusion_pos_70-500mz.m | Operator | SCSIO |
| Sample Name | luoxiaowei_lxw-DI46_pos | Instrument | maXis 255552.00029 |
| Comment | | | |

**Acquisition Parameter**

| | | | | | |
|---|---|---|---|---|---|
| Source Type | ESI | Ion Polarity | Positive | Set Nebulizer | 0.4 Bar |
| Focus | Active | Set Capillary | 4500 V | Set Dry Heater | 180 °C |
| Scan Begin | 70 m/z | Set End Plate Offset | -500 V | Set Dry Gas | 4.0 l/min |
| Scan End | 1500 m/z | Set Charging Voltage | 0 V | Set Divert Valve | Waste |
| | | Set Corona | 0 nA | Set APCI Heater | 0 °C |



| Meas. m/z | # | Ion Formula | Score | m/z | err [ppm] | err [mDa] | mSigma | rdb | e⁻ Conf | N-Rule |
|---|---|---|---|---|---|---|---|---|---|---|
| 403.174114 | 1 | C20H28NaO7 | 100.00 | 403.172724 | -3.4 | -1.4 | 85.0 | 6.5 | even | ok |
| 419.146993 | 1 | C20H28KO7 | 100.00 | 419.146661 | -0.8 | -0.3 | 55.8 | 6.5 | even | ok |
| 783.356276 | 1 | C40H56NaO14 | 100.00 | 783.356227 | 0.1 | 0.0 | 95.3 | 12.5 | even | ok |

**Figure S24.** HR-ESI-MS spectrum of (15*R*)-acetoxydothiorelone A (**2**)

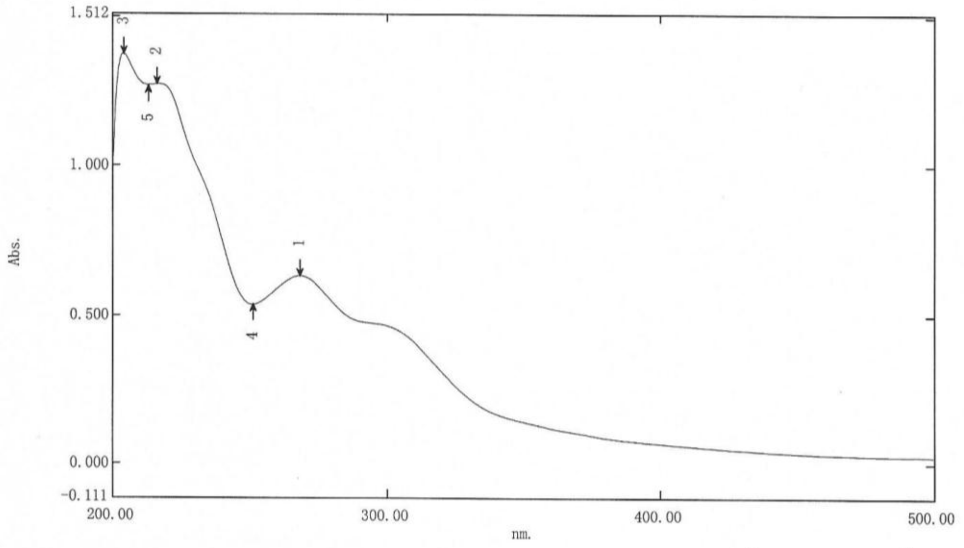


[测定属性]
波长范围 (nm.): 200.00 到 500.00
扫描速度: 中速
采样间隔: 0.2
自动采样间隔: 启用
扫描模式: 单个

[仪器属性]
仪器类型: UV-2600 系列
测定方式: 吸收值
狭缝宽: 2.0
积分时间: 0.1 秒.
光源转换波长: 323.0 nm
检测器单元: 直接
S/R 转换: 标准
阶梯校正: OFF

380.1 35μg/ml

| No. | 波长(nm) | 吸收值 | 描述 logε |
|---|---|---|---|
| 1 | 303.40 | 0.453 | 3.692 |
| 2 | 268.80 | 0.631 | 3.778 |
| 3 | 219.60 | 1.268 | 4.[illegible] (4.139) |
| 4 | 204.00 | 1.376 | 4.174 |
| 5 | | | |

**Figure S25.** UV spectrum of (15*R*)-acetoxydothiorelone A (**2**)

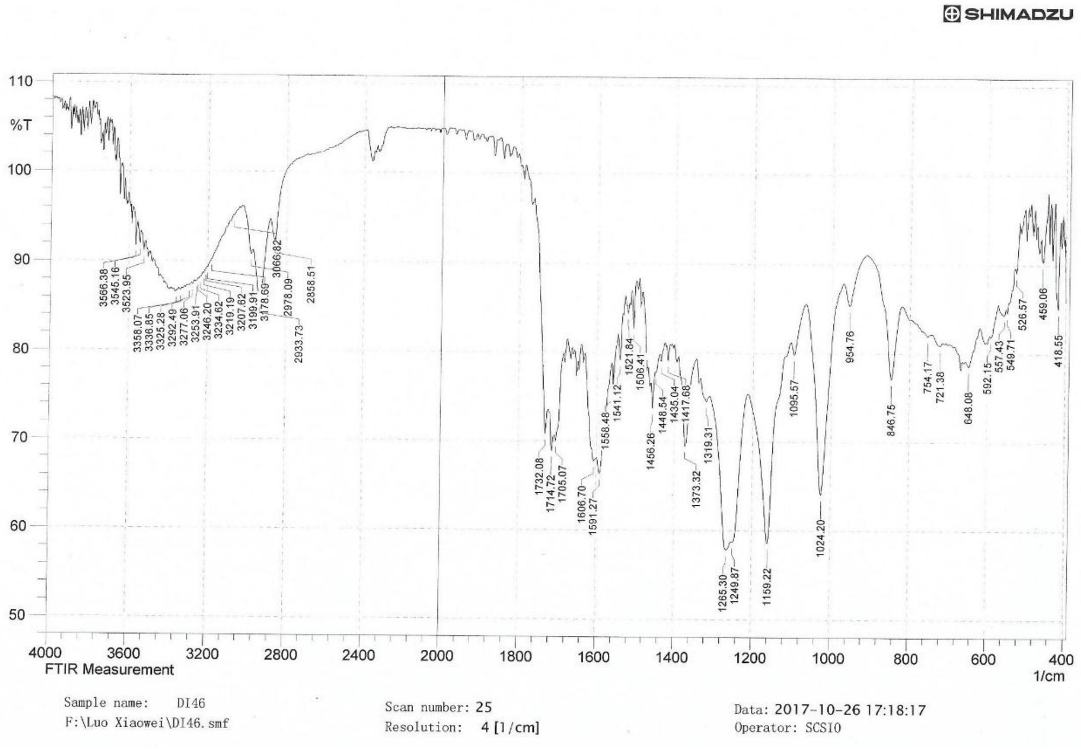


**Figure S26.** IR spectrum of (15*R*)-acetoxydothiorelone A (**2**)

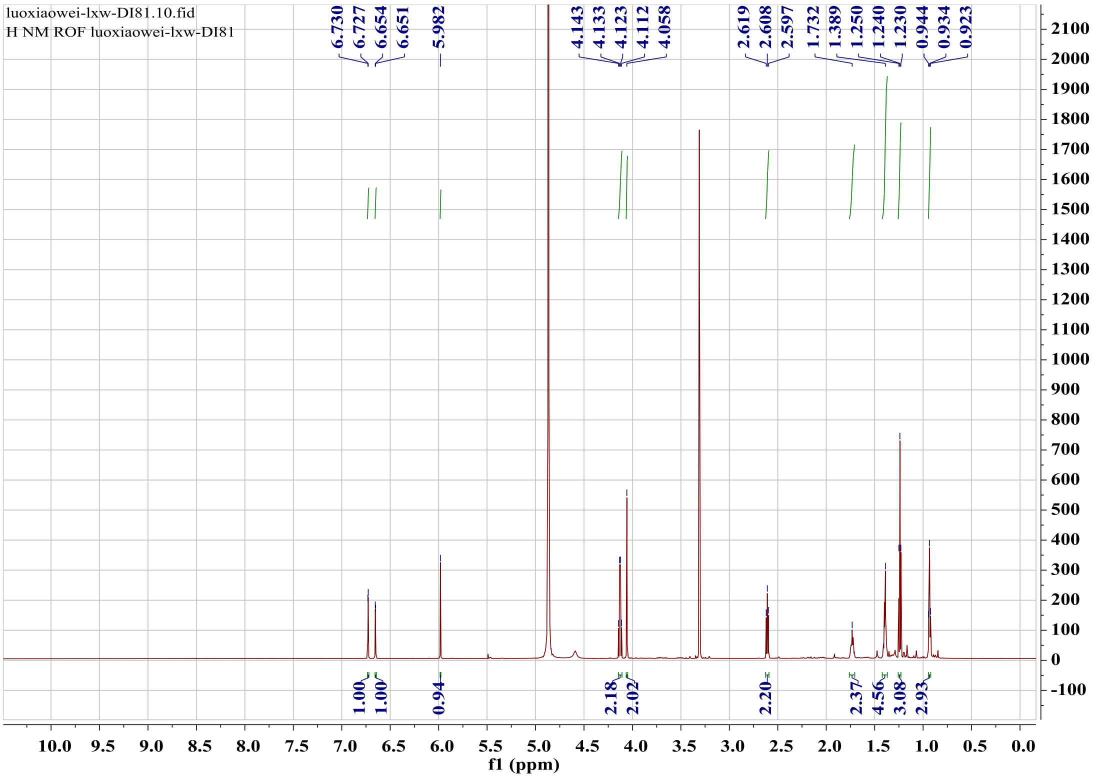


**Figure S27**. $^{1}$H NMR spectrum of pestalotiopsone H (**13**) ($CD_3OD$, 700 MHz)

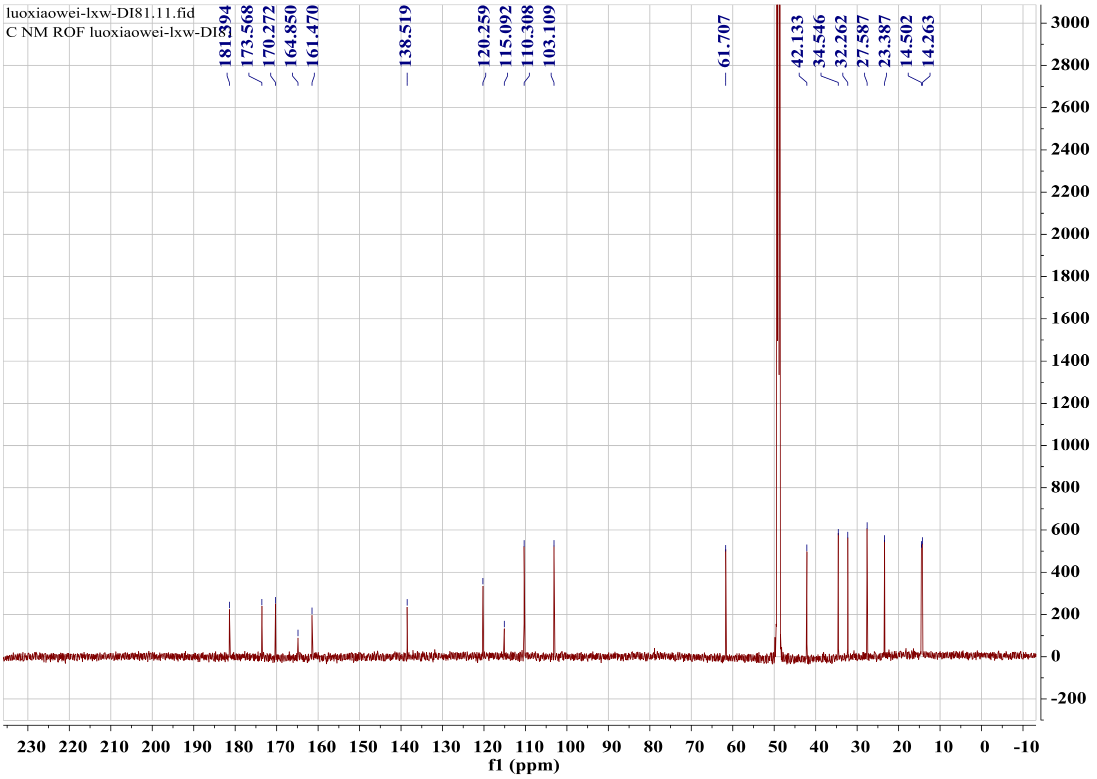

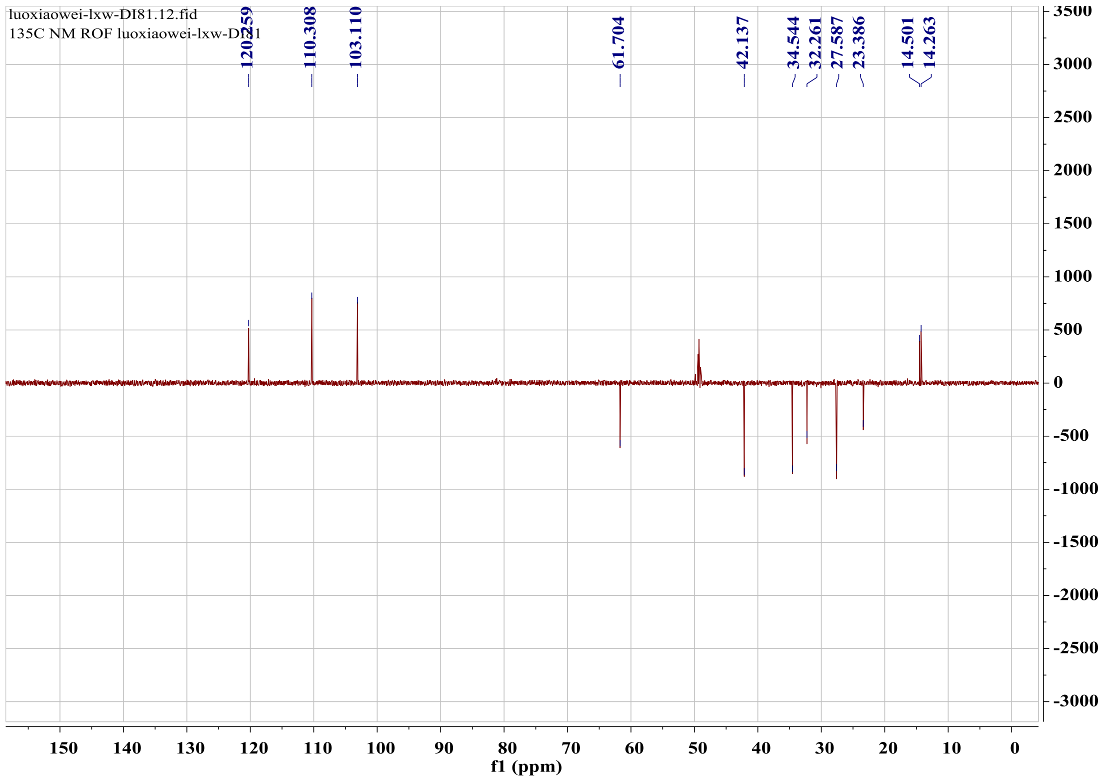


**Figure S28.** $^{13}$C NMR and DEPT spectra of pestalotiopsone H (**13**) ($CD_3OD$, 175 MHz)

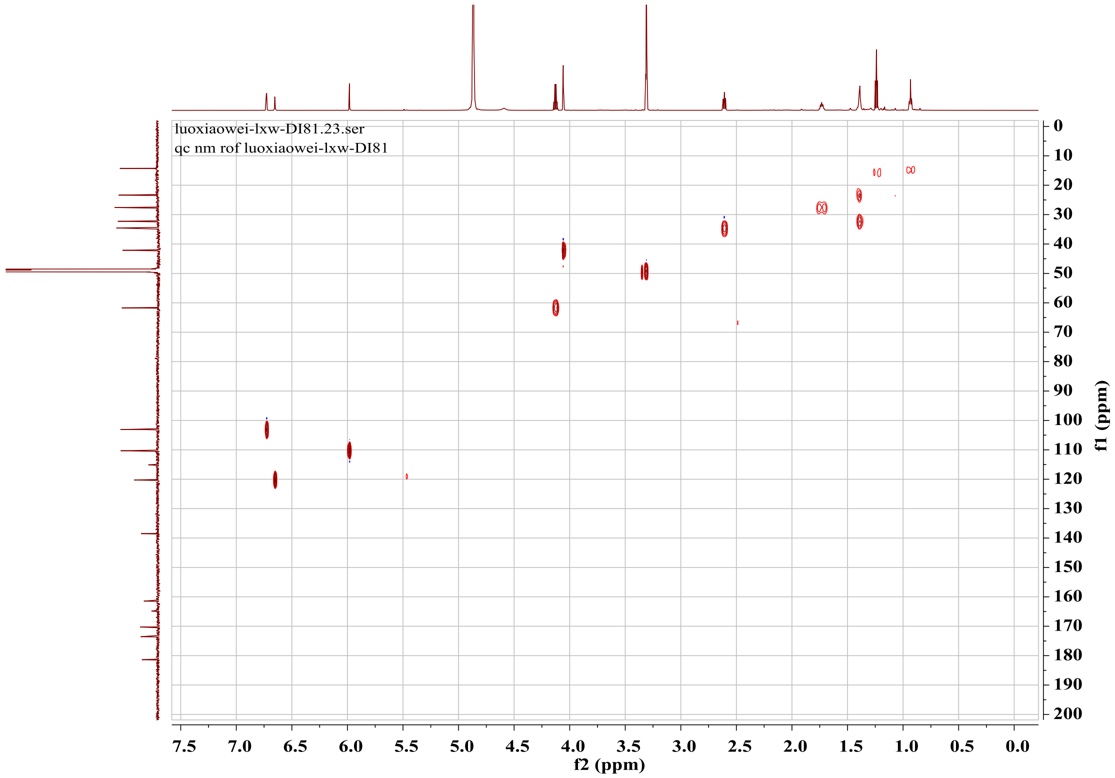


**Figure S29.** HSQC spectrum of pestalotiopsone H (**13**) ($CD_3OD$)

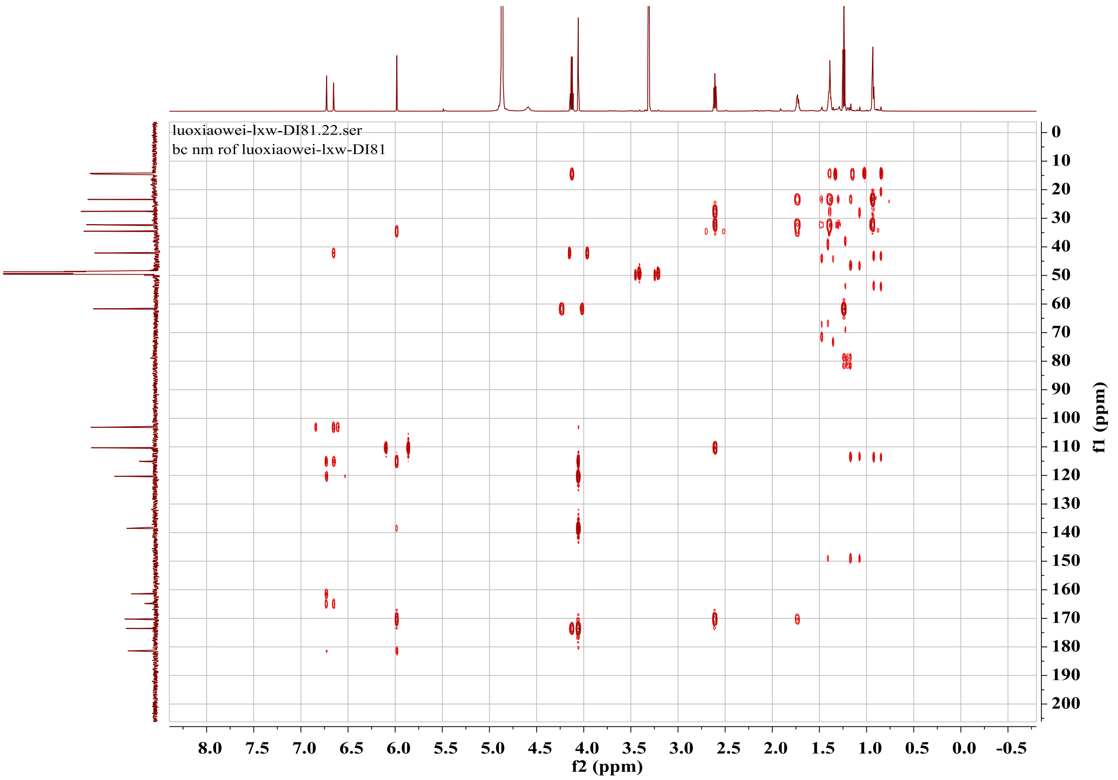


**Figure S30.** HMBC spectrum of pestalotiopsone H (**13**) ($CD_3OD$)

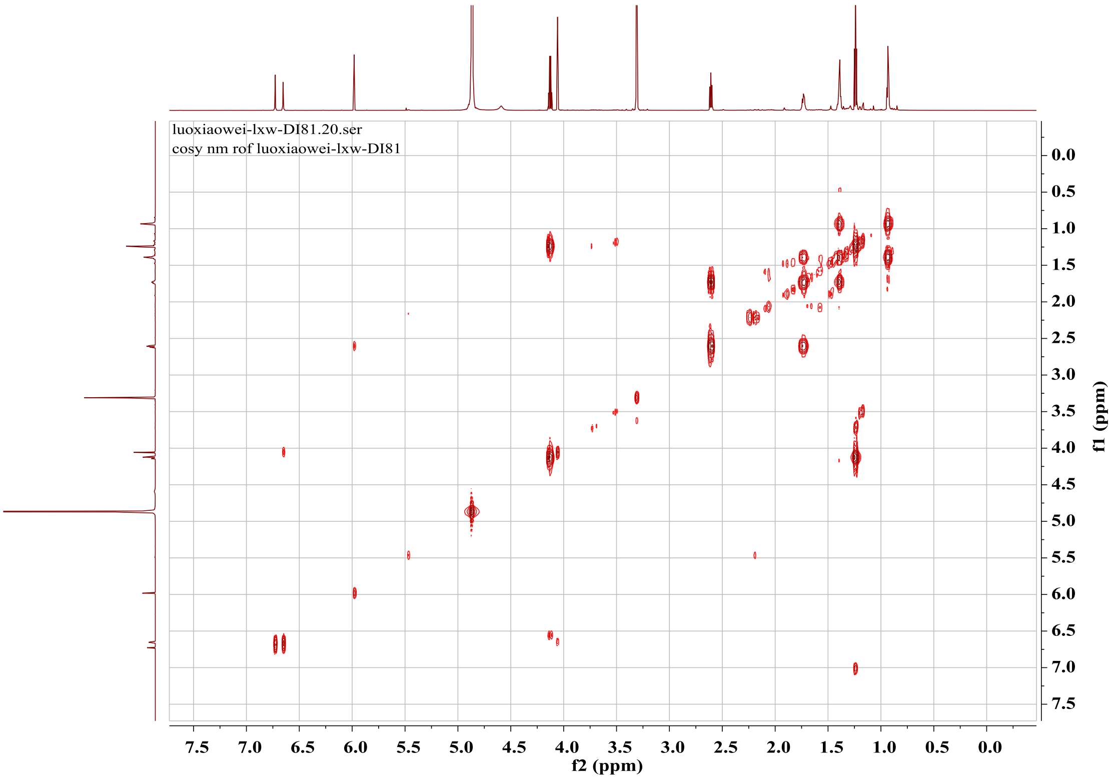


**Figure S31.** $^1H$-$^1H$ COSY spectrum of pestalotiopsone H (**13**) ($CD_3OD$)

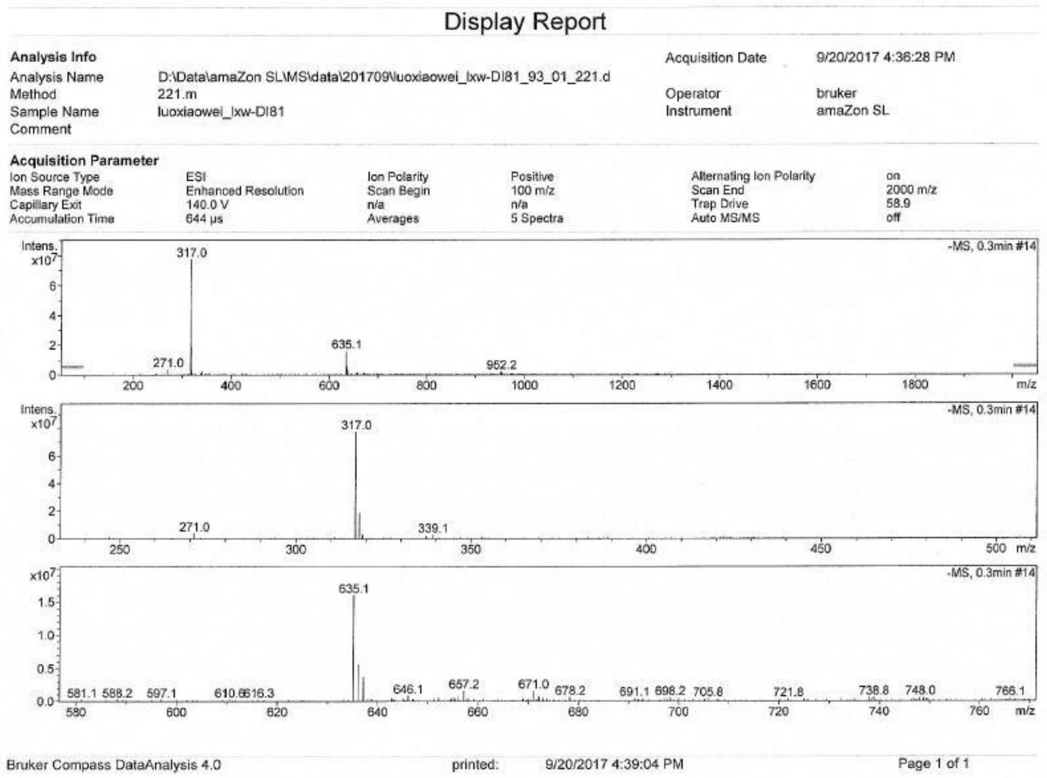
Display Report

Analysis Info
Analysis Name D:\Data\amaZon SL\MS\data\201709\luoxiaowei_lxw-DI81_93_01_221.d
Method 221.m
Sample Name luoxiaowei_lxw-DI81
Comment
Acquisition Date 9/20/2017 4:36:28 PM
Operator bruker
Instrument amaZon SL

| Acquisition Parameter | | | | | |
|---|---|---|---|---|---|
| Ion Source Type | ESI | Ion Polarity | Positive | Alternating Ion Polarity | on |
| Mass Range Mode | Enhanced Resolution | Scan Begin | 100 m/z | Scan End | 2000 m/z |
| Capillary Exit | 140.0 V | n/a | n/a | Trap Drive | 58.9 |
| Accumulation Time | 644 µs | Averages | 5 Spectra | Auto MS/MS | off |



Bruker Compass DataAnalysis 4.0 printed: 9/20/2017 4:39:04 PM Page 1 of 1

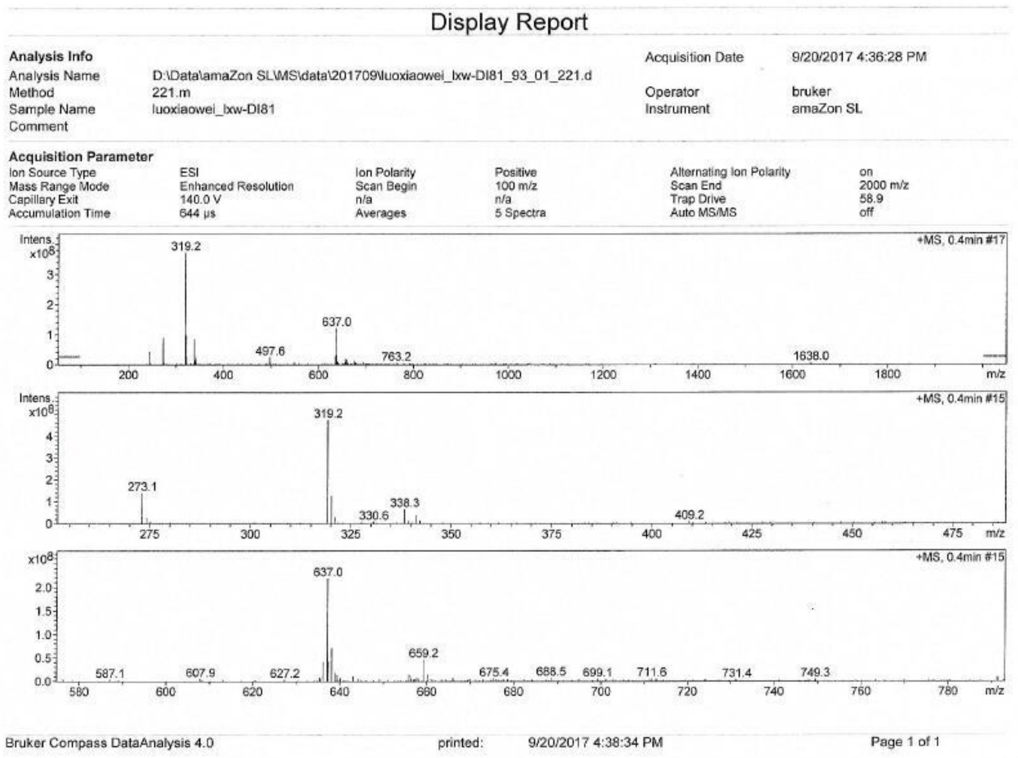
Display Report

Analysis Info
Analysis Name D:\Data\amaZon SL\MS\data\201709\luoxiaowei_lxw-DI81_93_01_221.d
Method 221.m
Sample Name luoxiaowei_lxw-DI81
Comment
Acquisition Date 9/20/2017 4:36:28 PM
Operator bruker
Instrument amaZon SL

| Acquisition Parameter | | | | | |
|---|---|---|---|---|---|
| Ion Source Type | ESI | Ion Polarity | Positive | Alternating Ion Polarity | on |
| Mass Range Mode | Enhanced Resolution | Scan Begin | 100 m/z | Scan End | 2000 m/z |
| Capillary Exit | 140.0 V | n/a | n/a | Trap Drive | 58.9 |
| Accumulation Time | 644 µs | Averages | 5 Spectra | Auto MS/MS | off |



Bruker Compass DataAnalysis 4.0 printed: 9/20/2017 4:38:34 PM Page 1 of 1

**Figure S32.** Positive and negative LR-ESI-MS spectra of pestalotiopsone H (**13**)

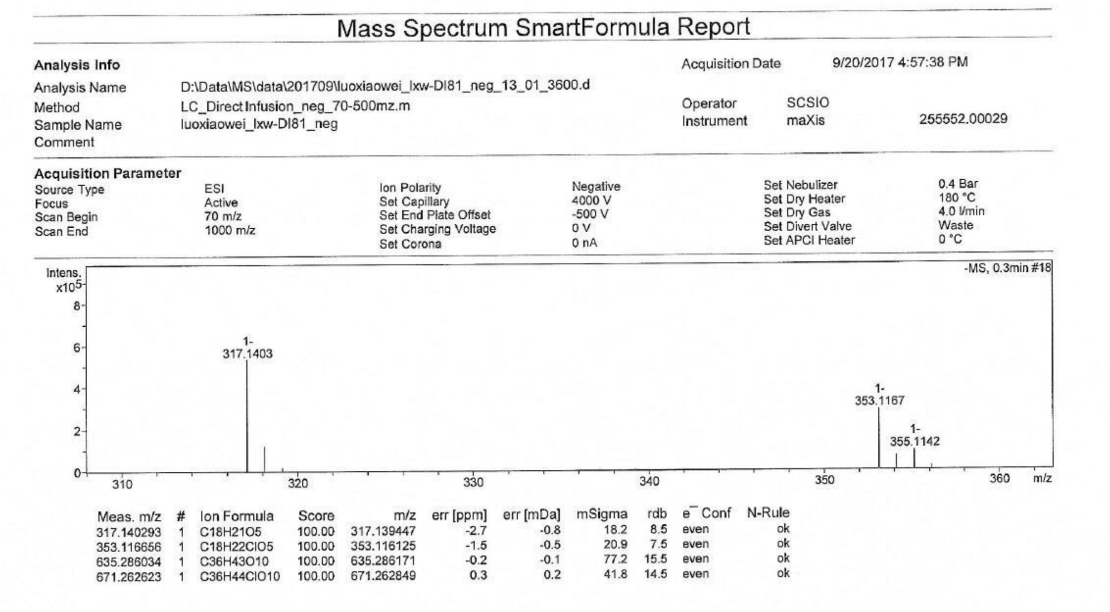


**Figure S33.** HR-ESI-MS spectrum of pestalotiopsone H (**13**)

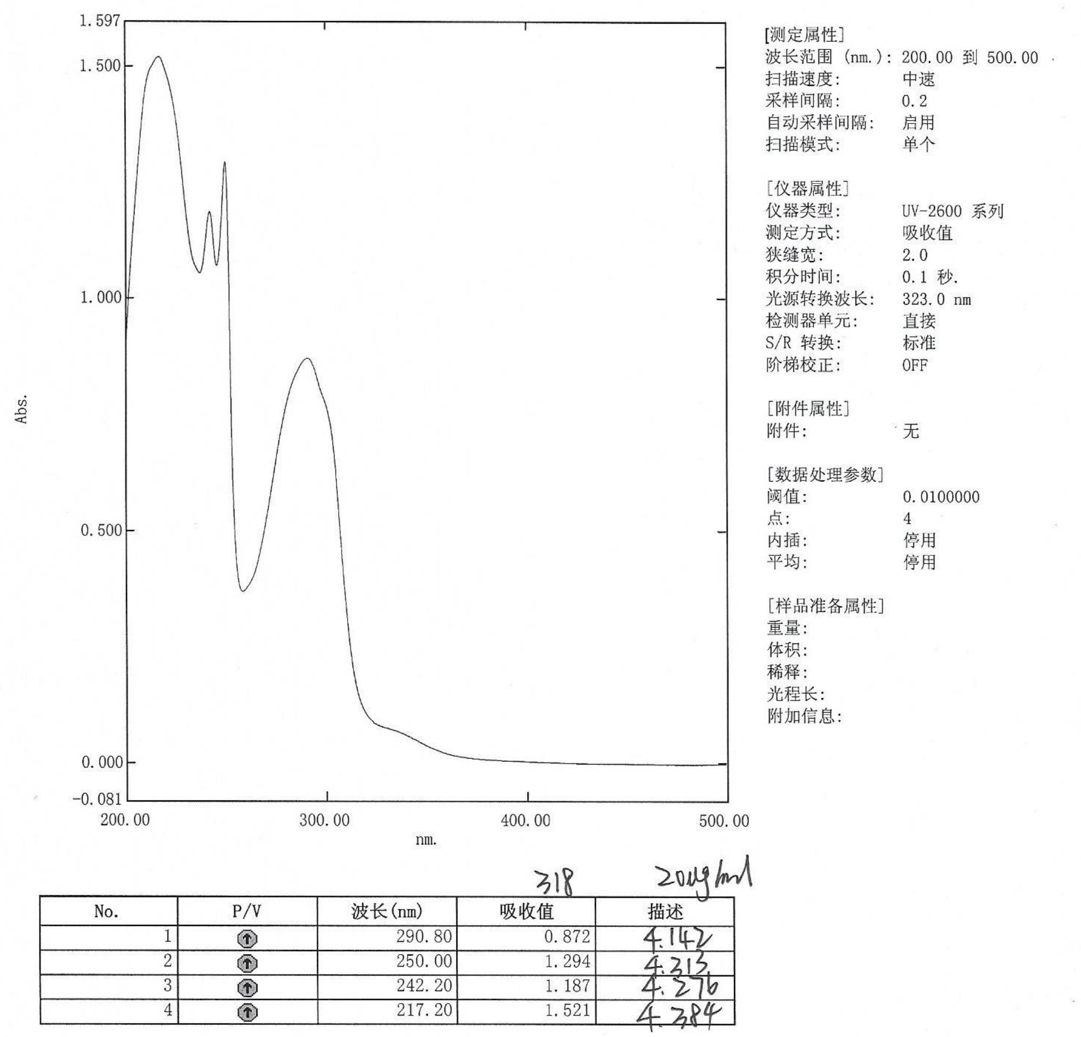


**Figure S34.** UV spectrum of pestalotiopsone H (**13**)

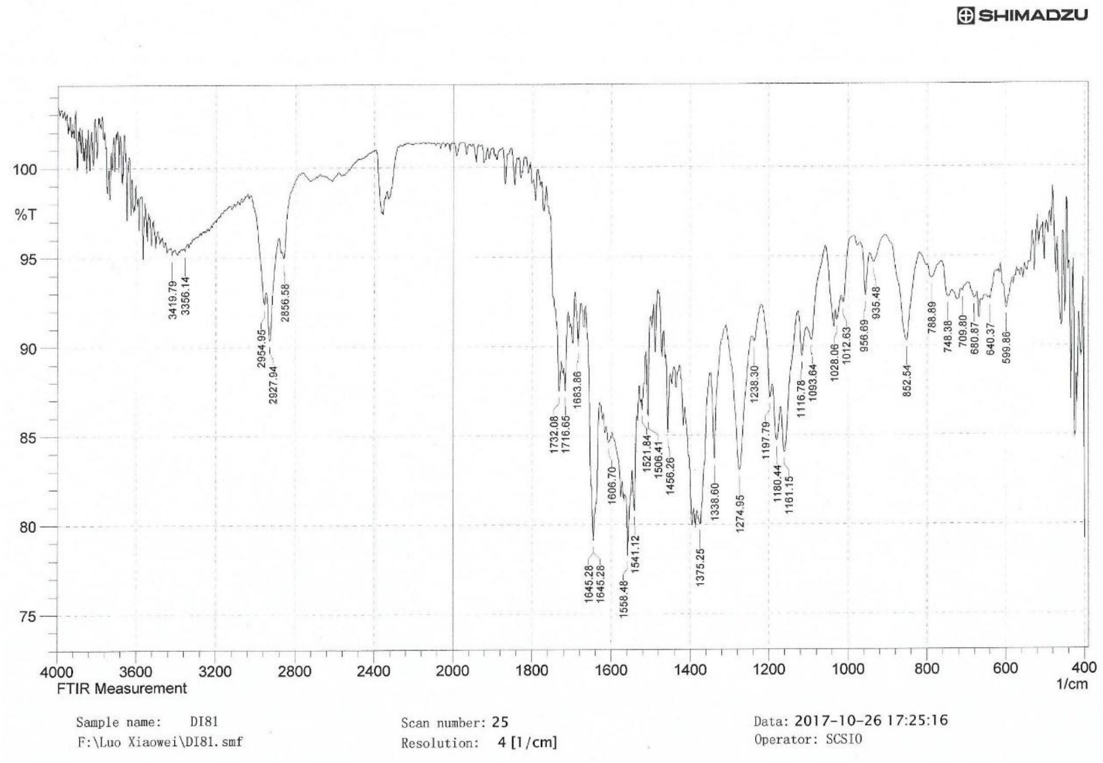


**Figure S35.** IR spectrum of pestalotiopsone H (**13**)

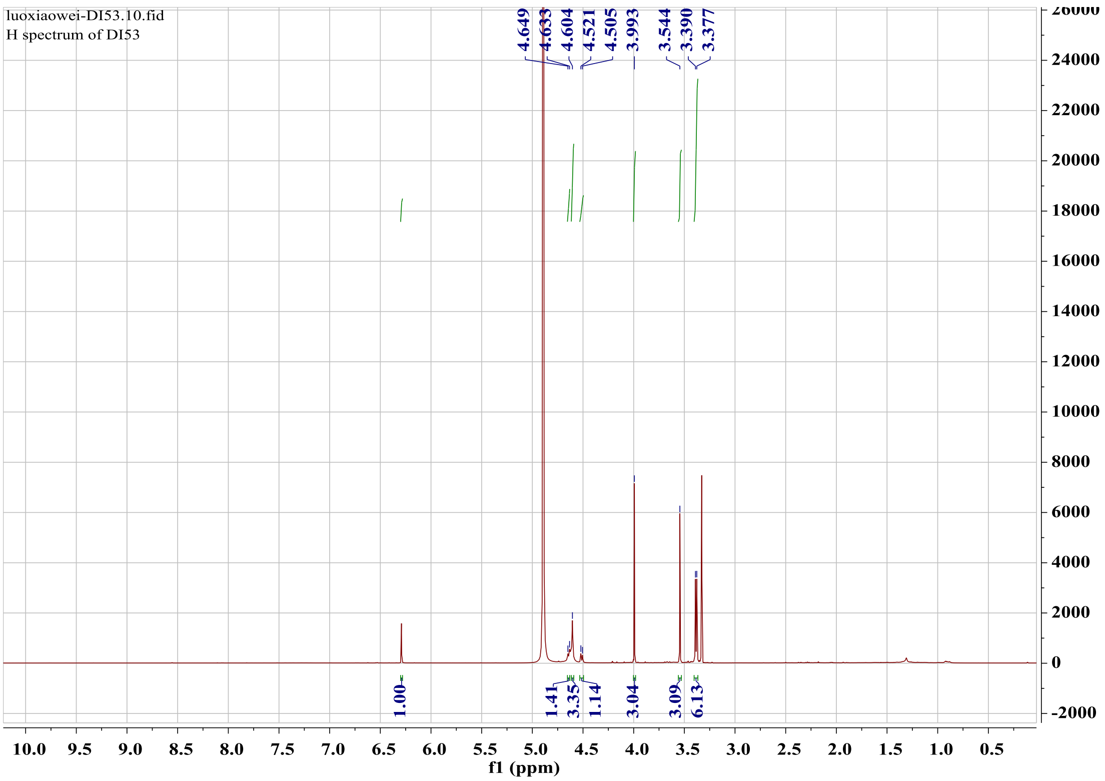


**Figure S36**. $^{1}$H NMR spectrum of (±)-microsphaerophthalide H (**17**) ($CD_3OD$, 700 MHz)

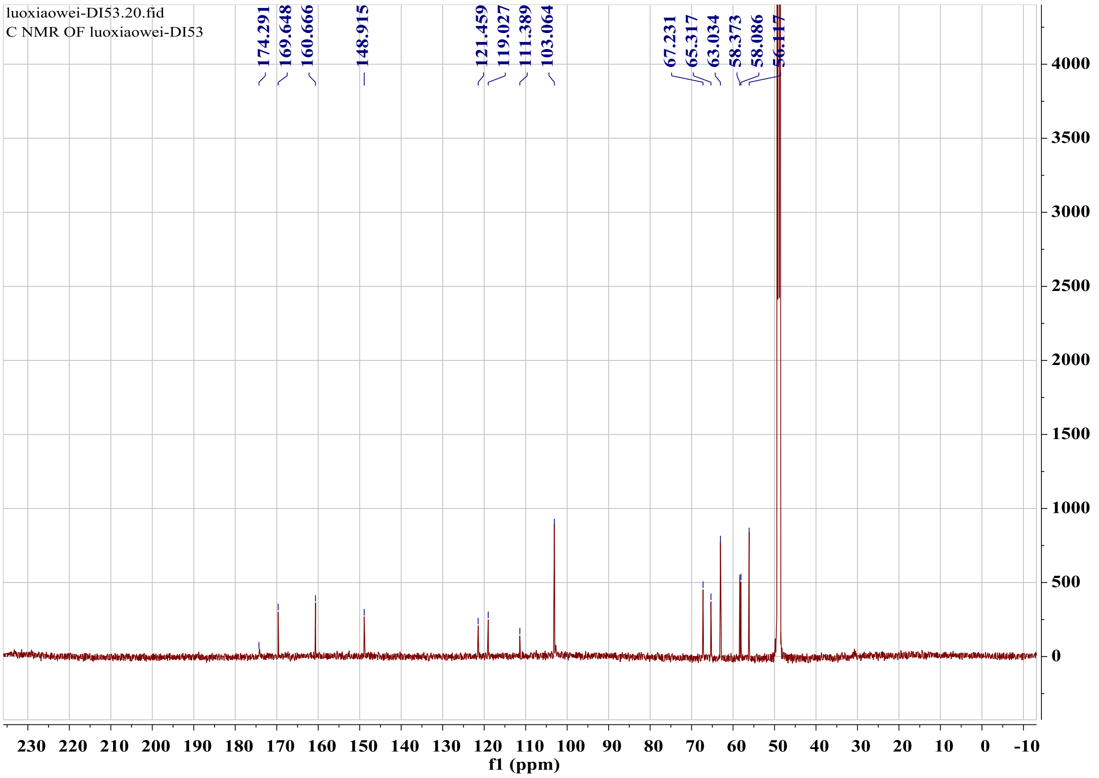


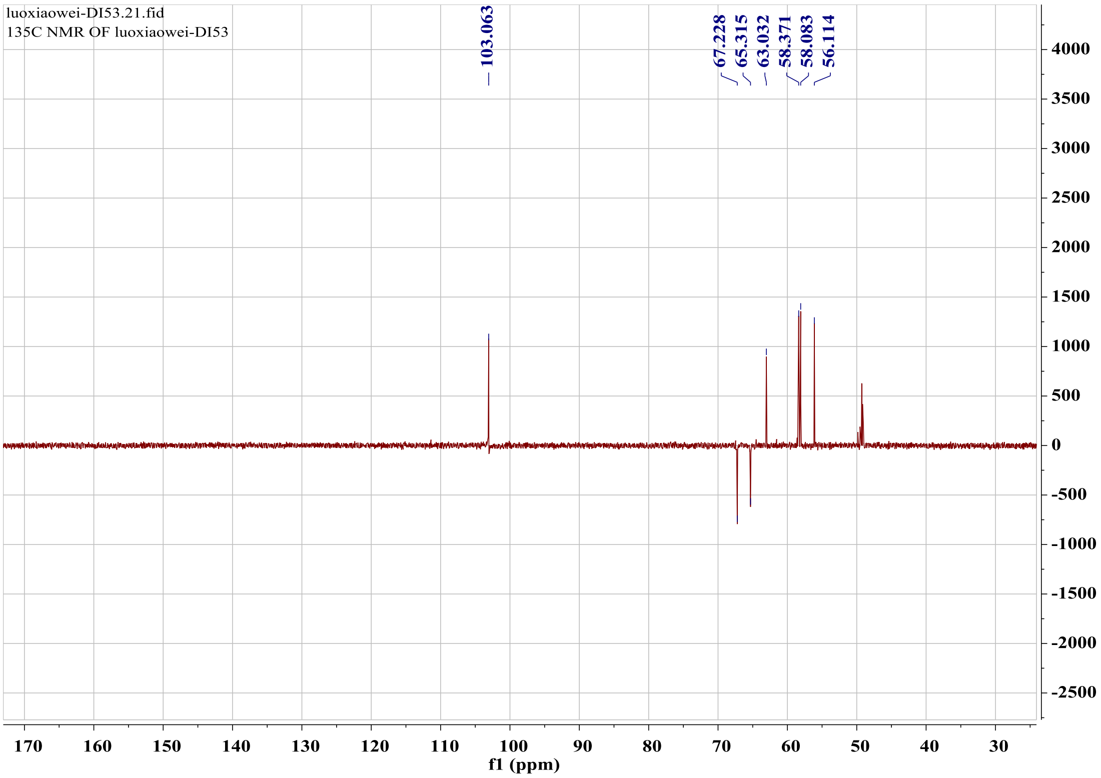


**Figure S37.** $^{13}$C NMR and DEPT spectra of (±)-microsphaerophthalide H (**17**) (CD$_3$OD, 175 MHz)

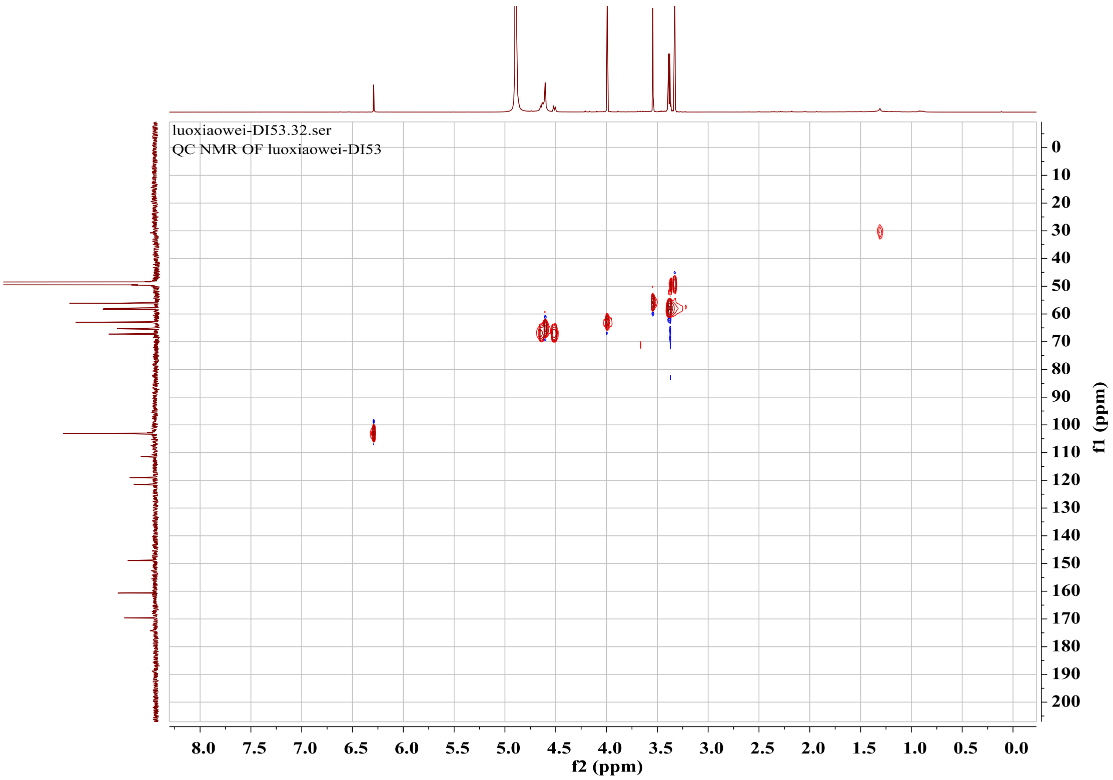


**Figure S38.** HSQC spectrum of (±)-microsphaerophthalide H (**17**) ($CD_3OD$)

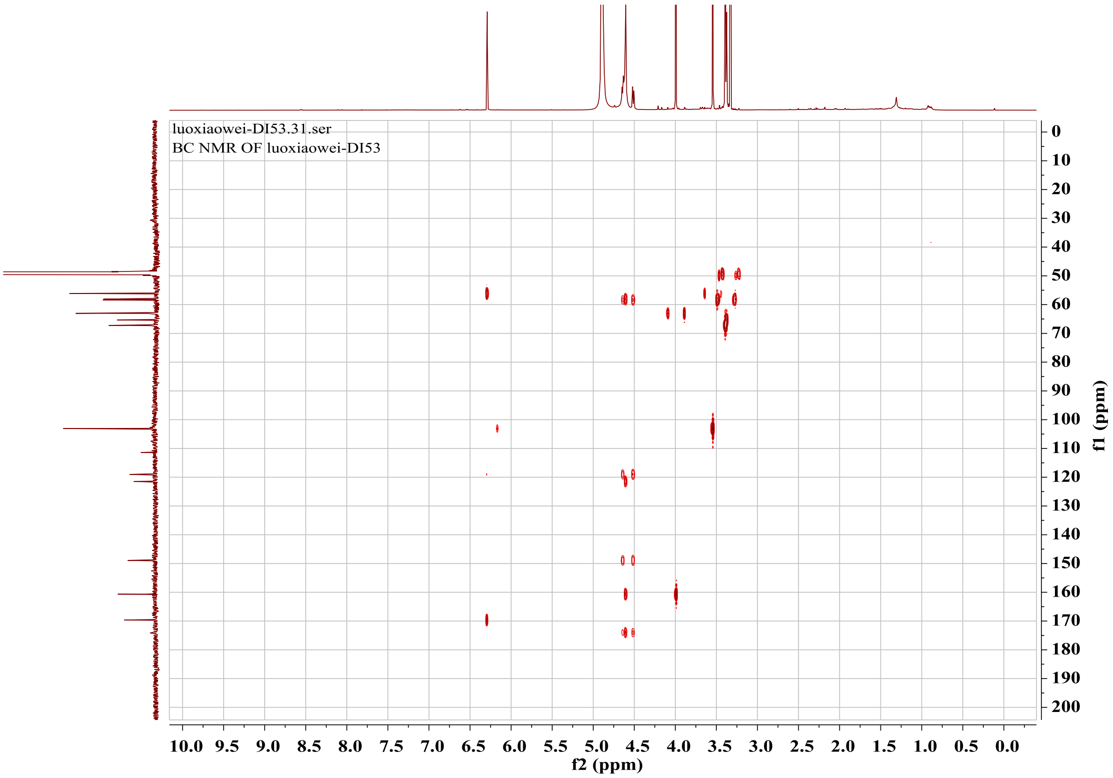


**Figure S39.** HMBC spectrum of (±)-microsphaerophthalide H (**17**) ($CD_3OD$)

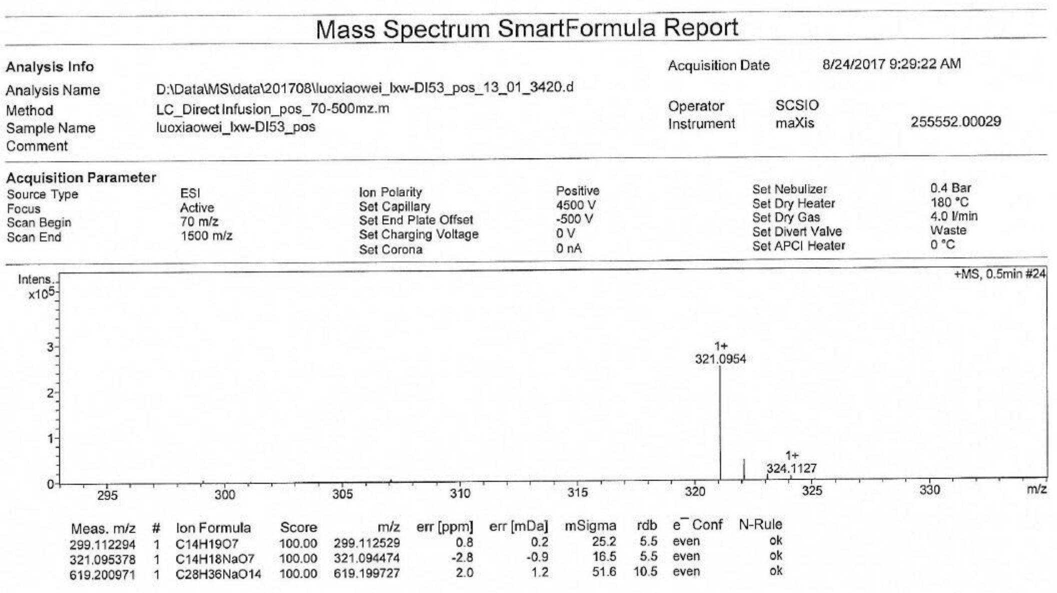


**Figure S40.** HR-ESI-MS spectrum of (±)-microsphaerophthalide H (**17**)

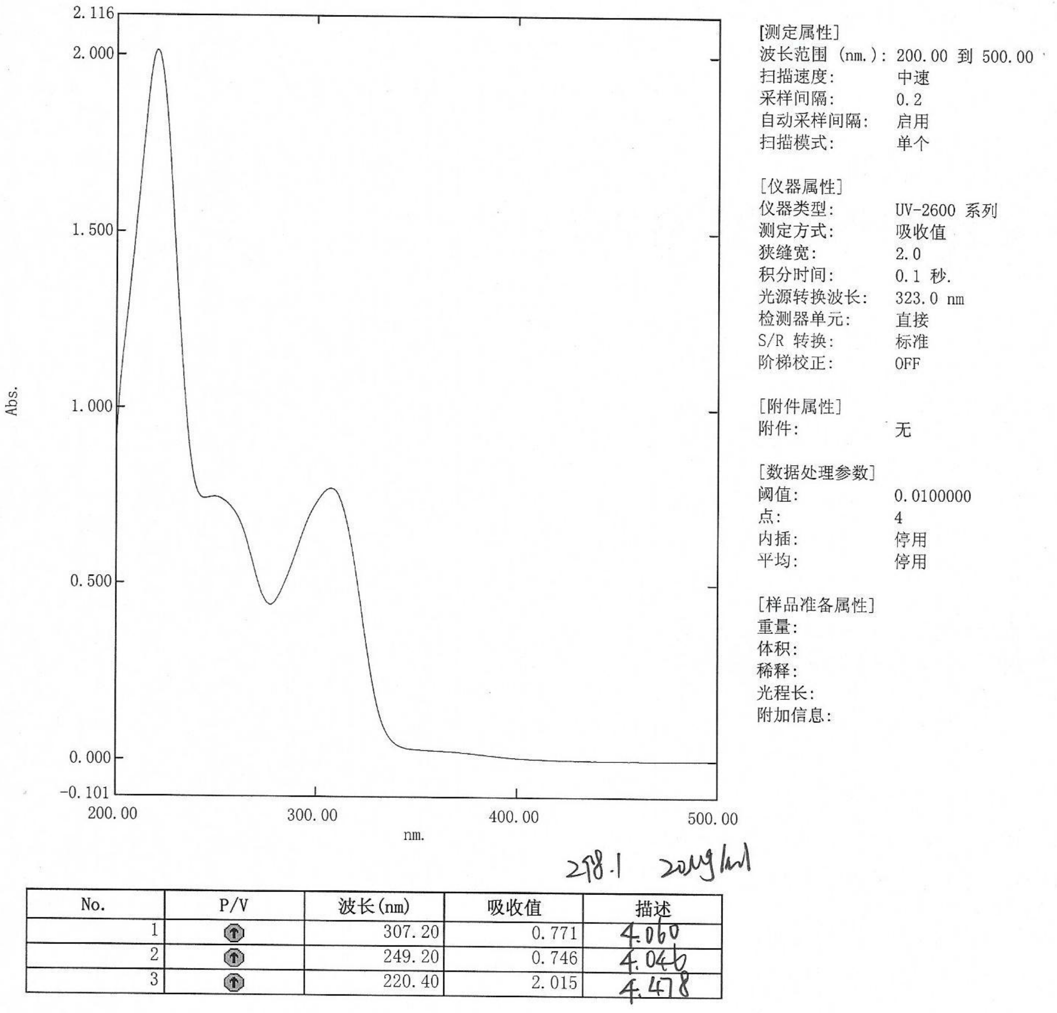


**Figure S41.** UV spectrum of (±)-microsphaerophthalide H (**17**)

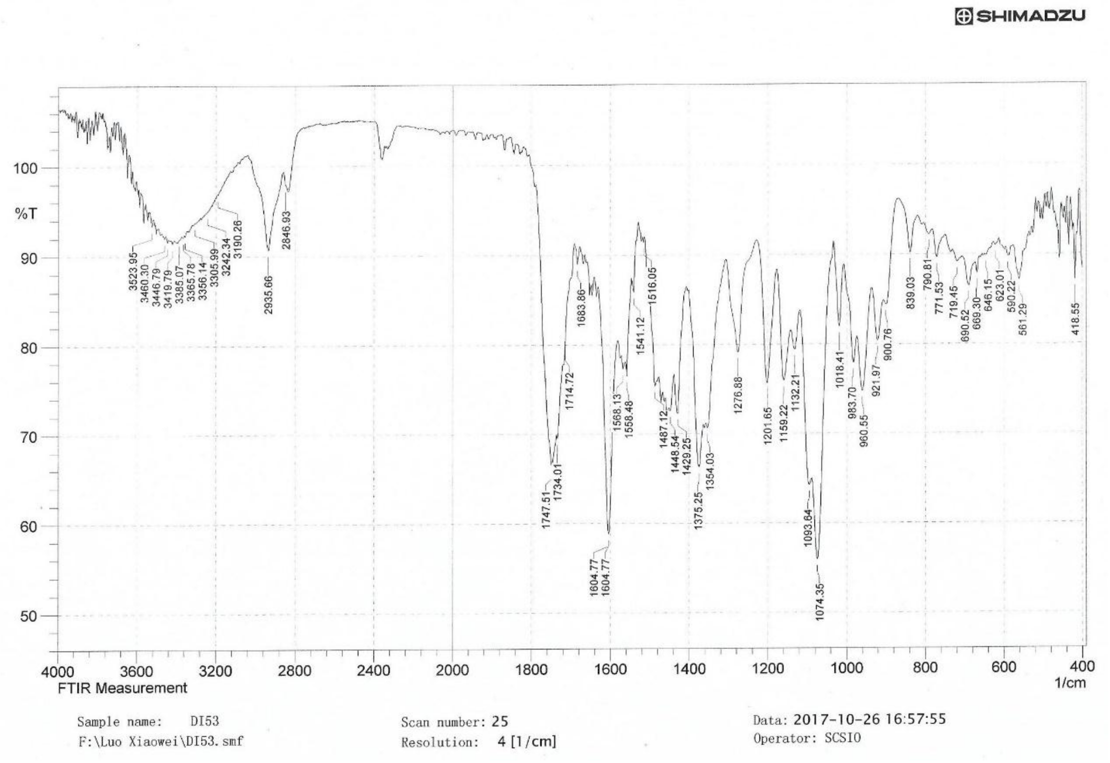


**Figure S42.** IR spectrum of (±)-microsphaerophthalide H (**17**)

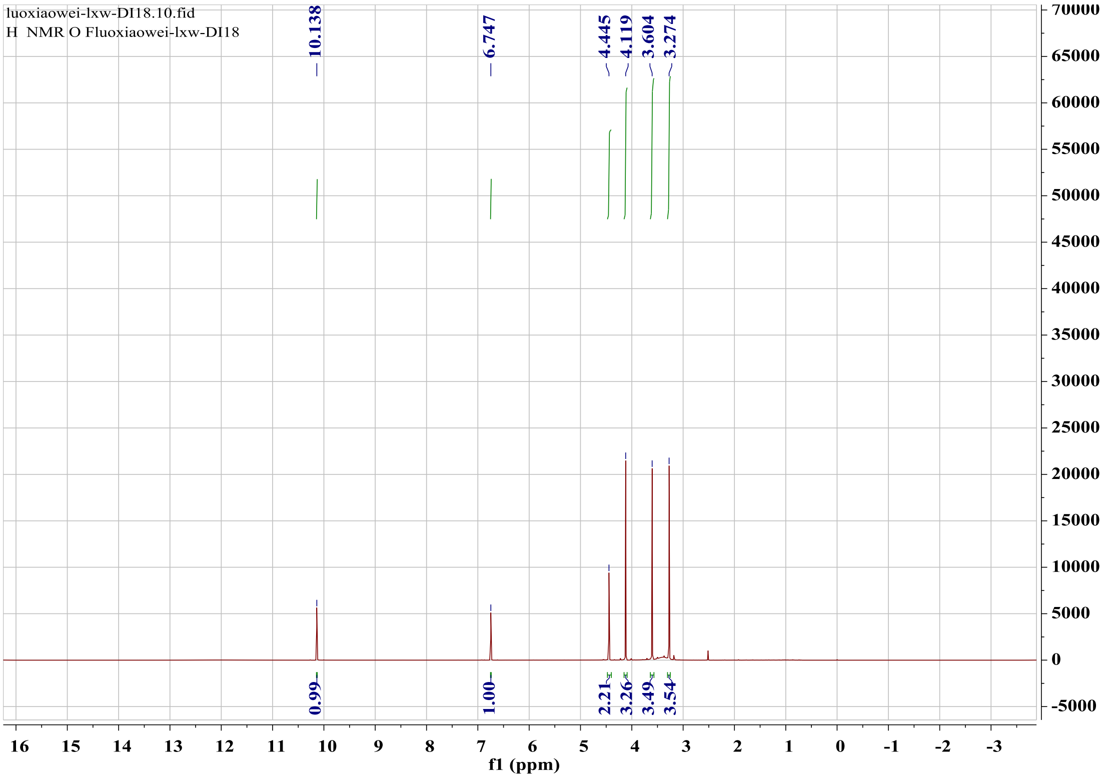


**Figure S43**. $^{1}$H NMR spectrum of microsphaerophthalide I (**18**) (DMSO-$d_6$, 700 MHz)

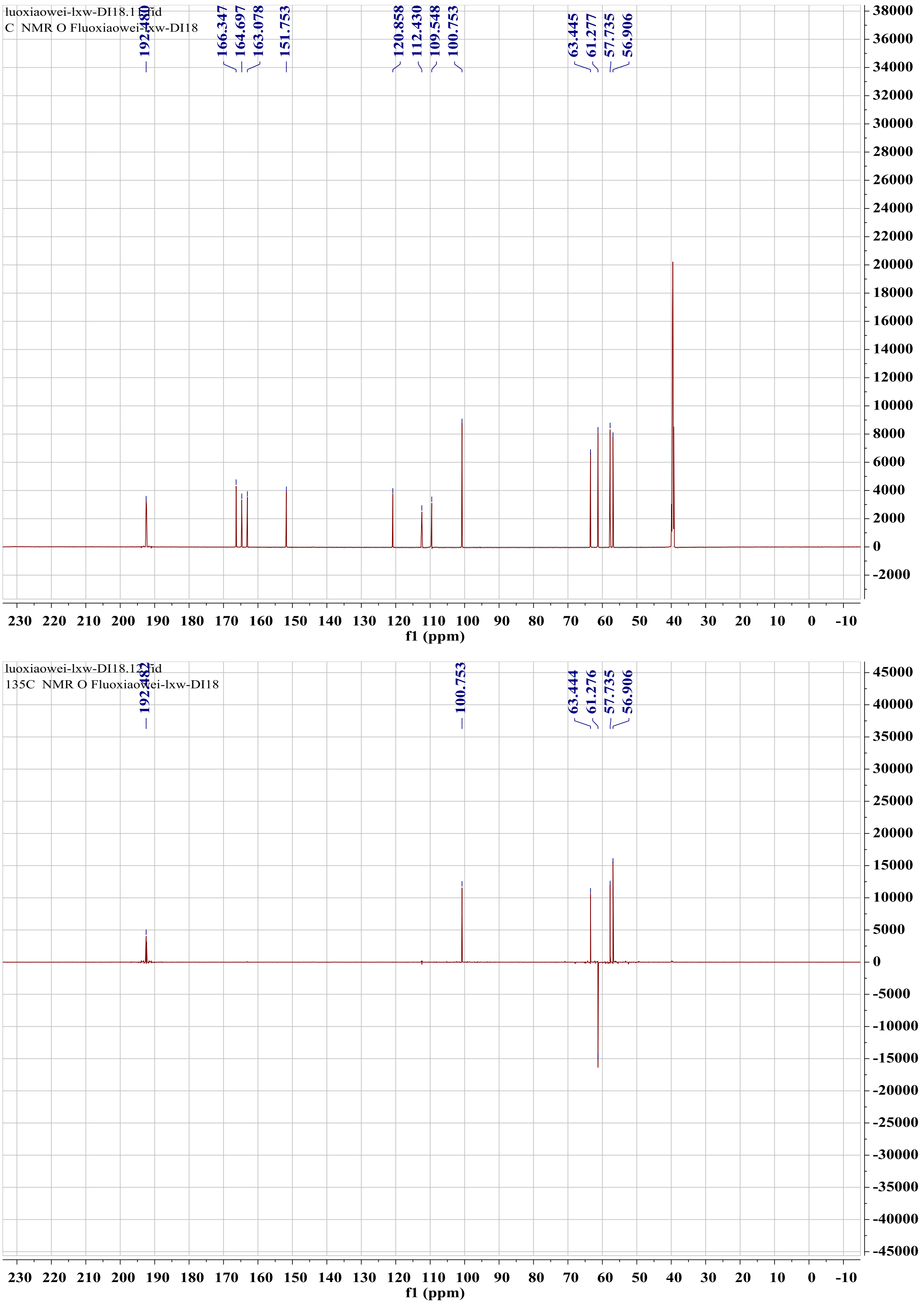


**Figure S44.** $^{13}$C NMR and DEPT spectra of microsphaerophthalide I (**18**) (DMSO-$d_6$, 175 MHz)

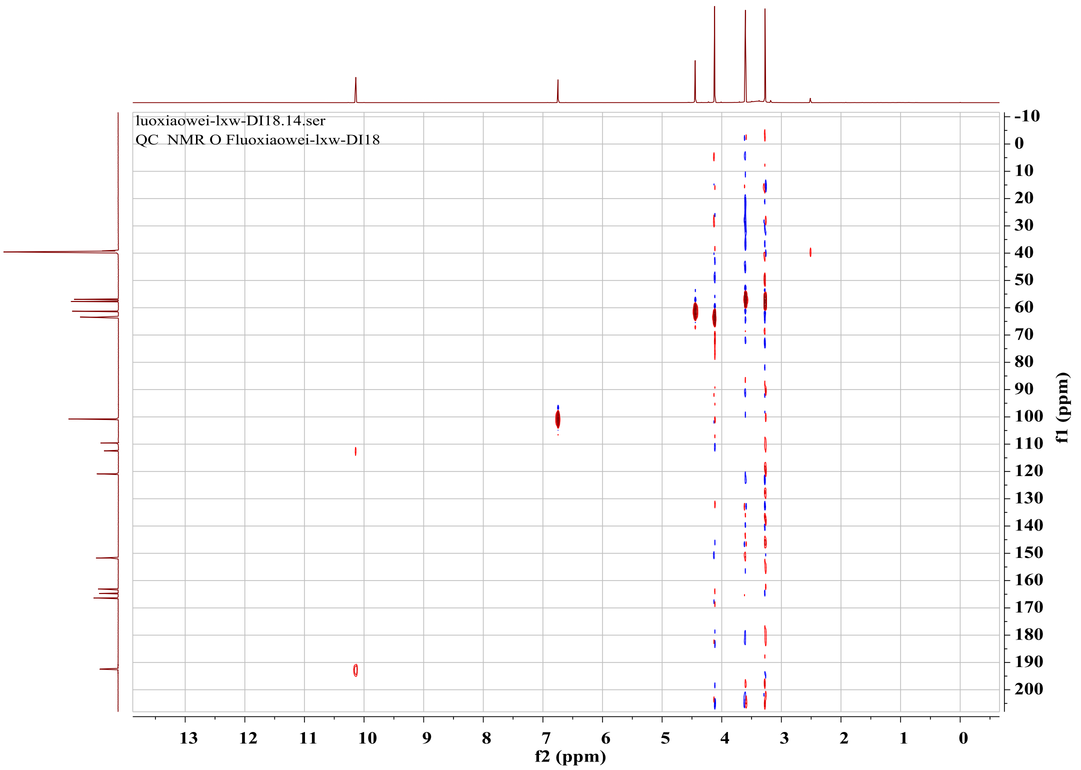


**Figure S45.** HSQC spectrum of microsphaerophthalide I (**18**) (DMSO-$d_6$)

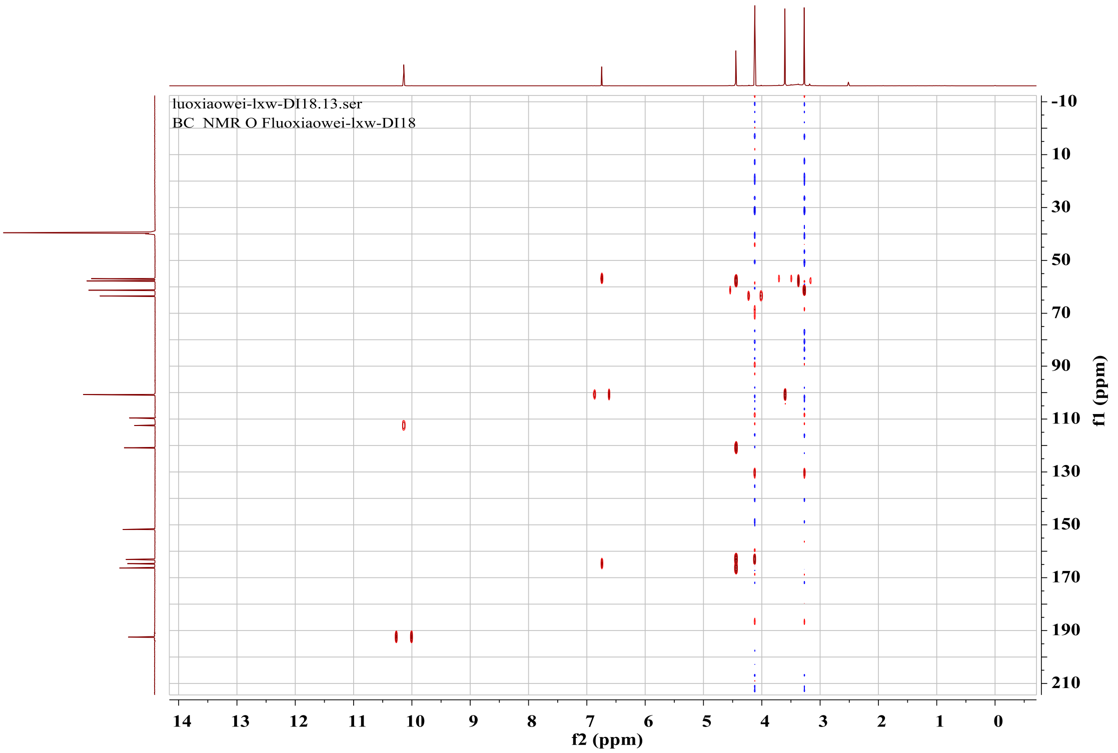


**Figure S46.** HMBC spectrum of microsphaerophthalide I (**18**) (DMSO-$d_6$)

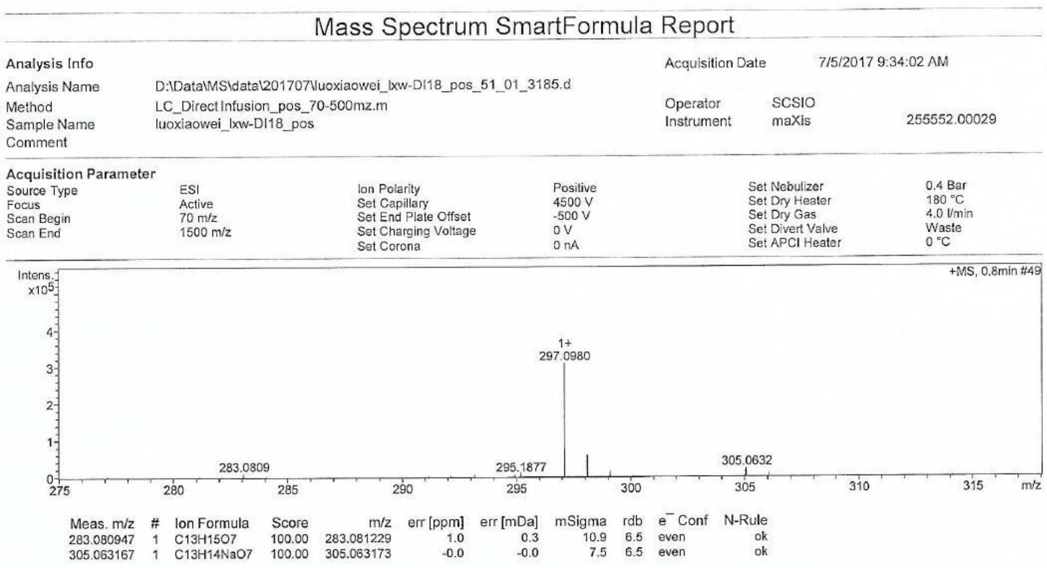


**Figure S47.** HR-ESI-MS spectrum of microsphaerophthalide I (**18**)

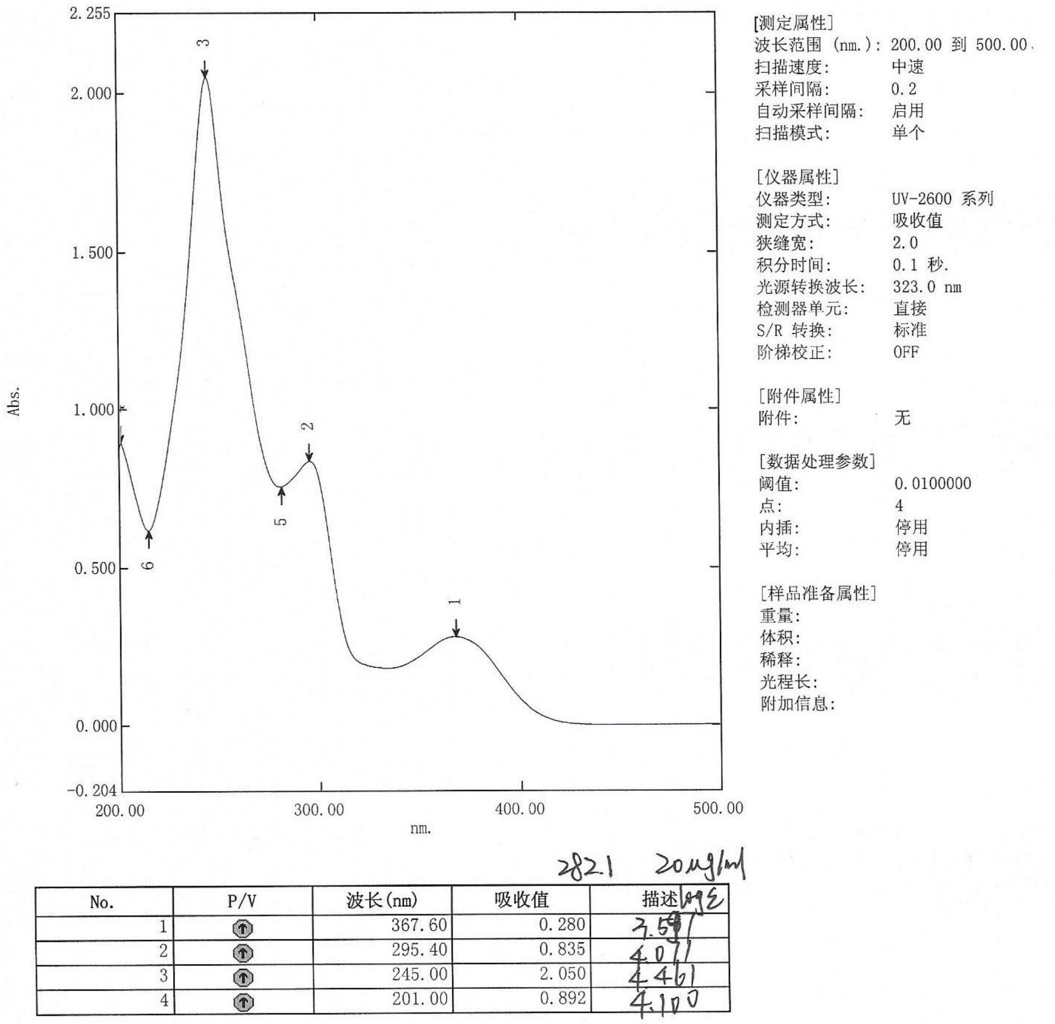


**Figure S48.** UV spectrum of microsphaerophthalide I (**18**)

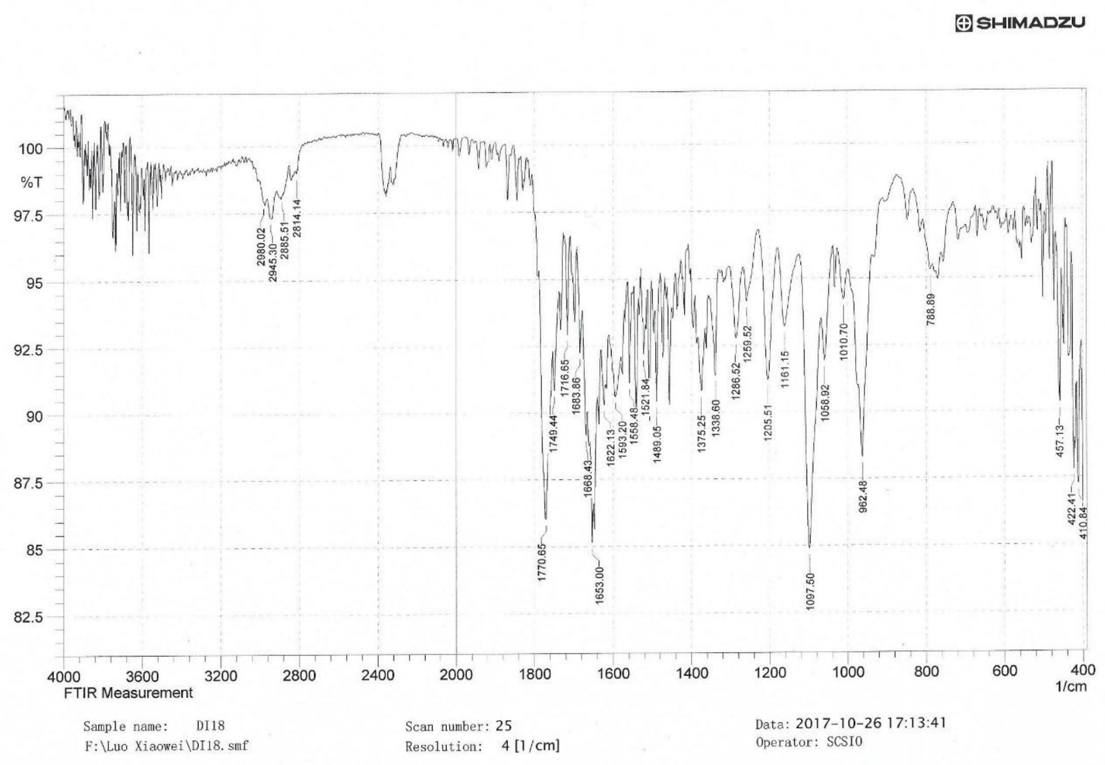


**Figure S49.** IR spectrum of microsphaerophthalide I (**18**)

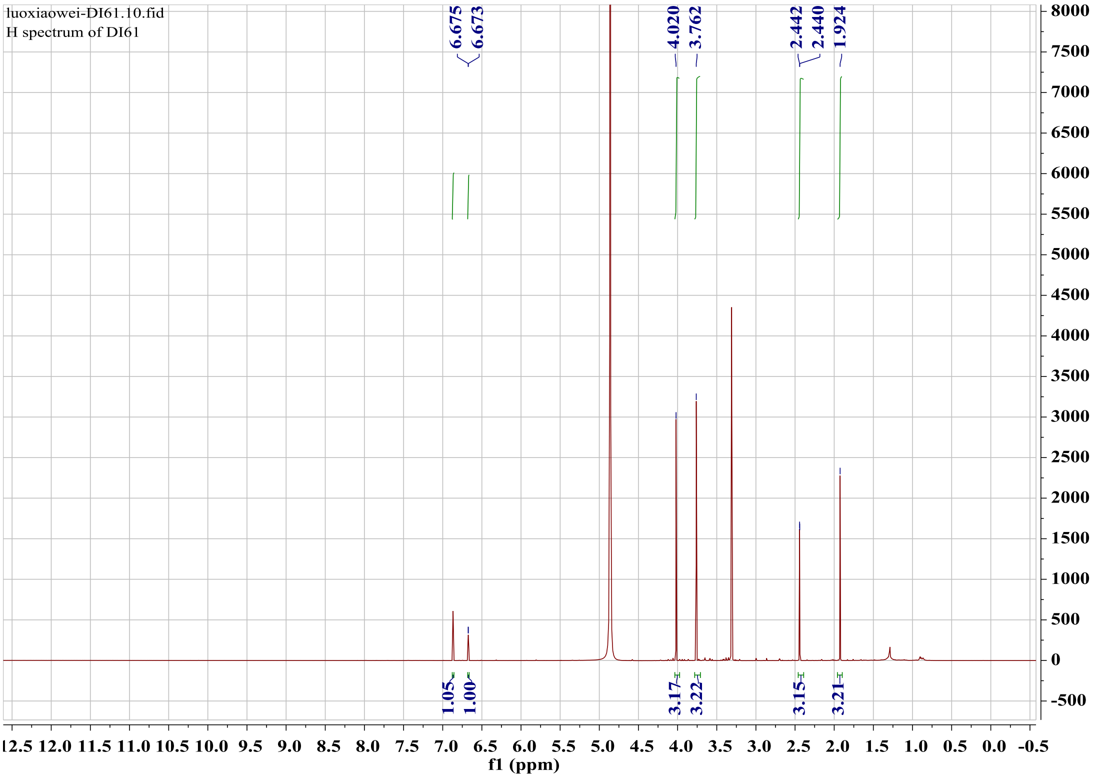


**Figure S50**. $^{1}$H NMR spectrum of methyl convolvulopyrone (**21**) ($CD_3OD$, 700 MHz)

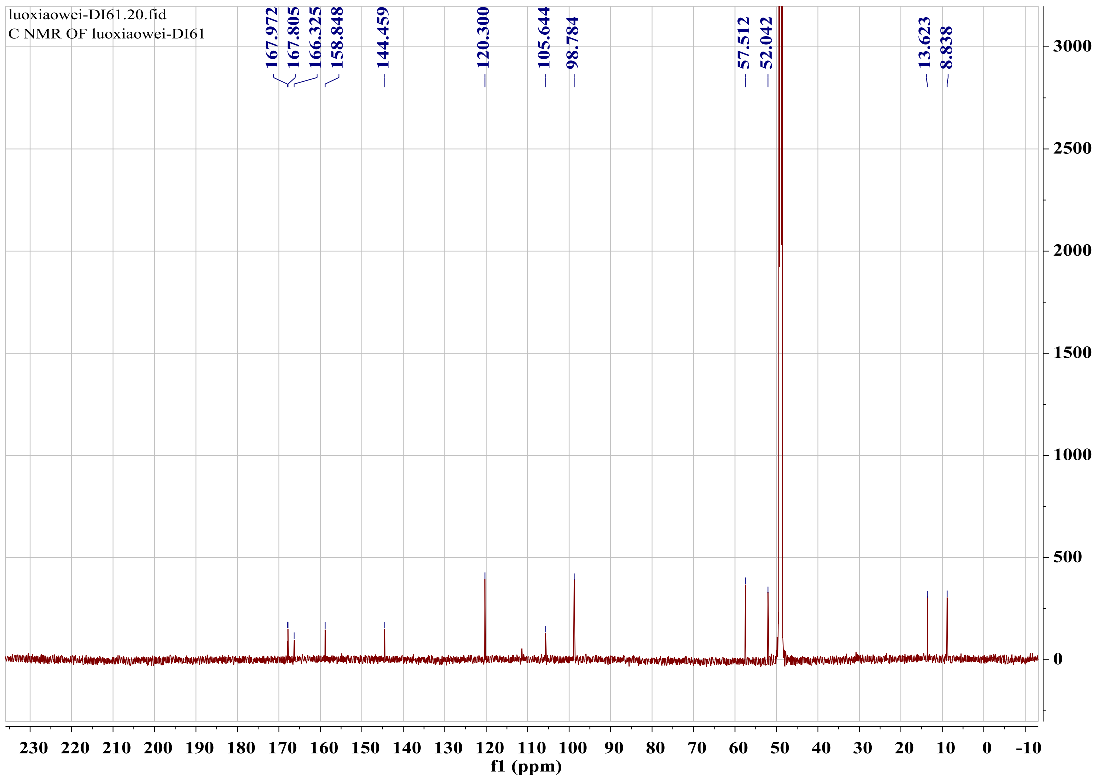


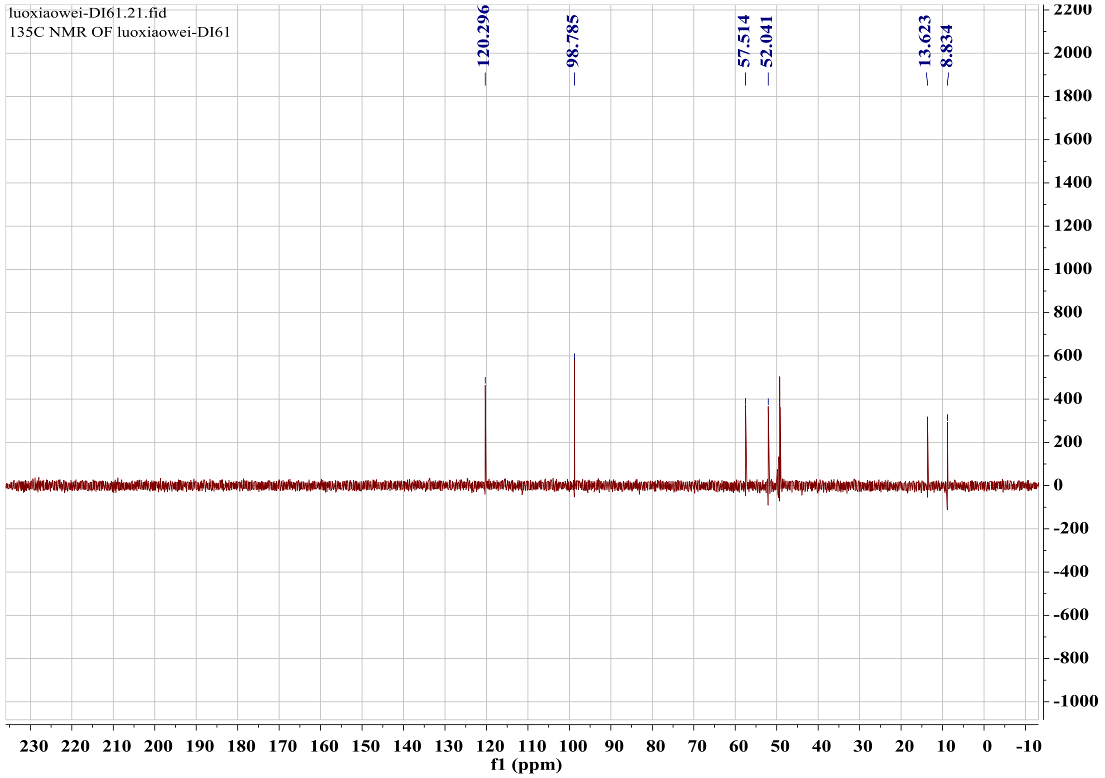


**Figure S51.** $^{13}C$ NMR and DEPT spectra of methyl convolvulopyrone (**21**) ($CD_3OD$, 175 MHz)

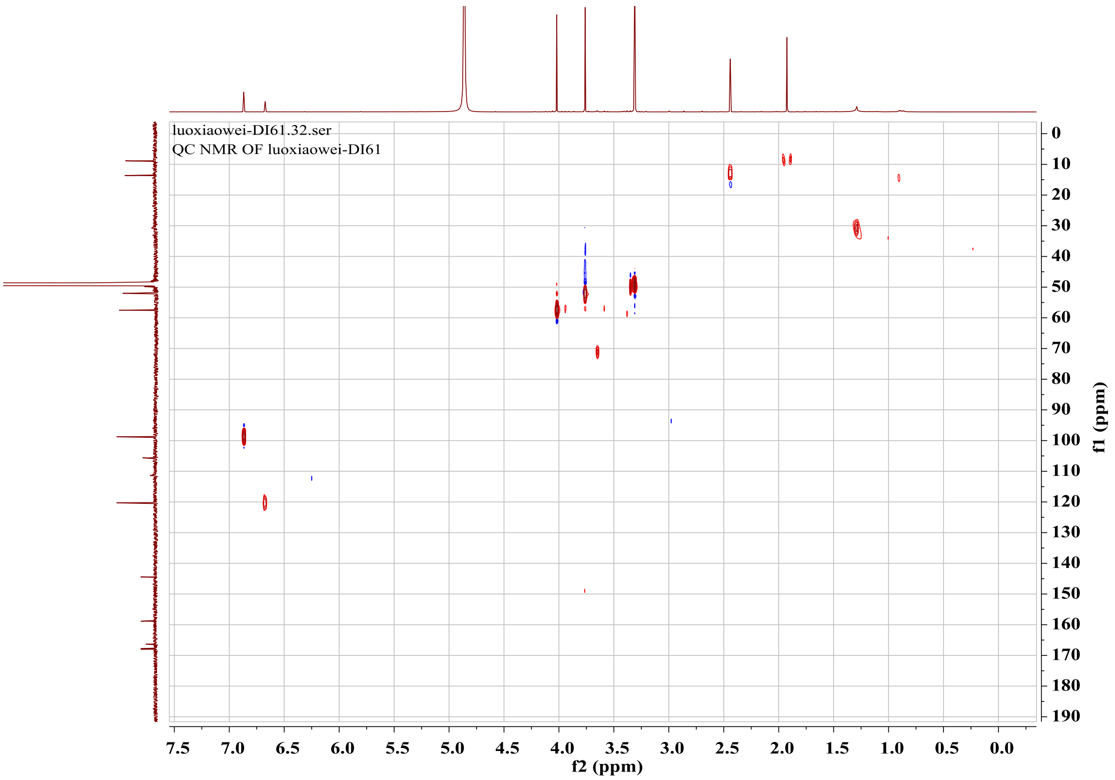


**Figure S52.** HSQC spectrum of methyl convolvulopyrone (**21**) ($CD_3OD$)

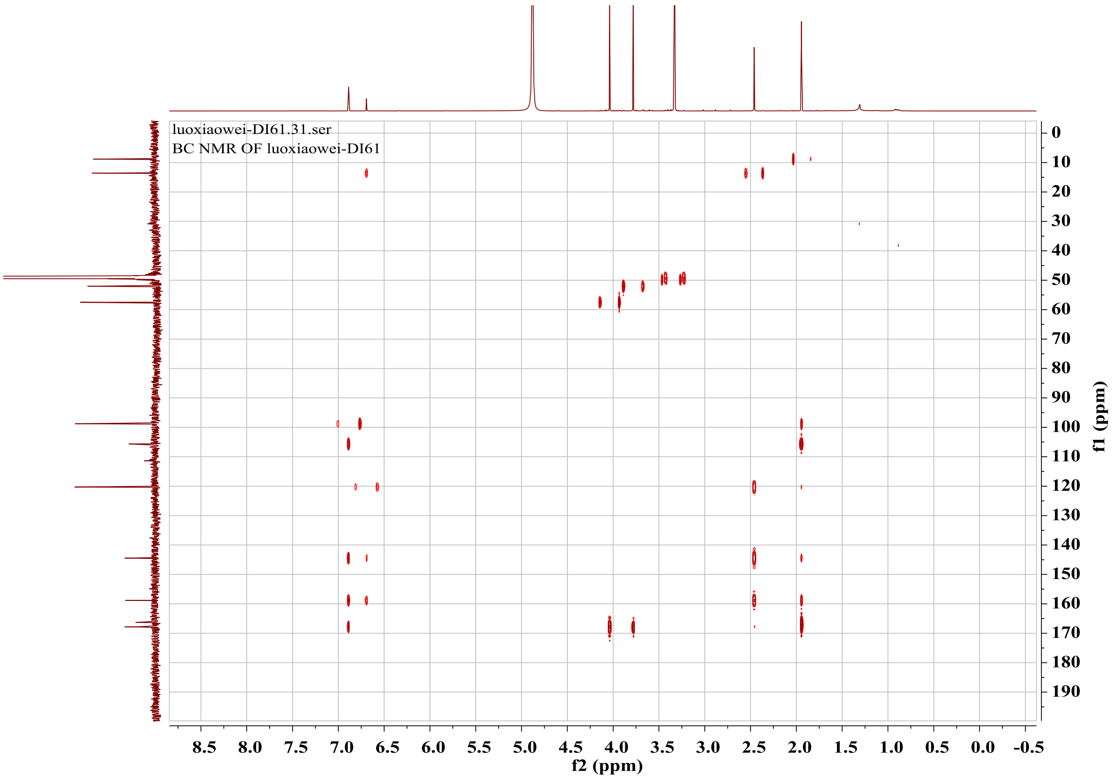


**Figure S53.** HMBC spectrum of methyl convolvulopyrone (**21**) ($CD_3OD$)

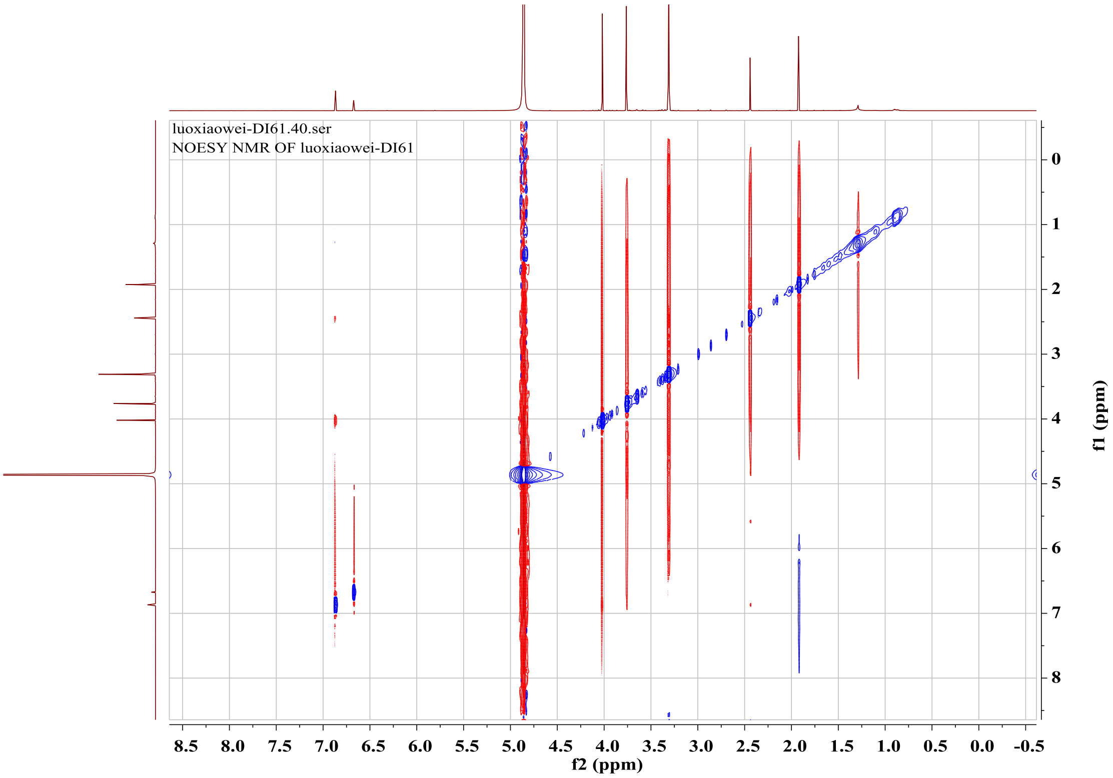


**Figure S54.** NOESY spectrum of methyl convolvulopyrone (**21**) ($CD_3OD$)

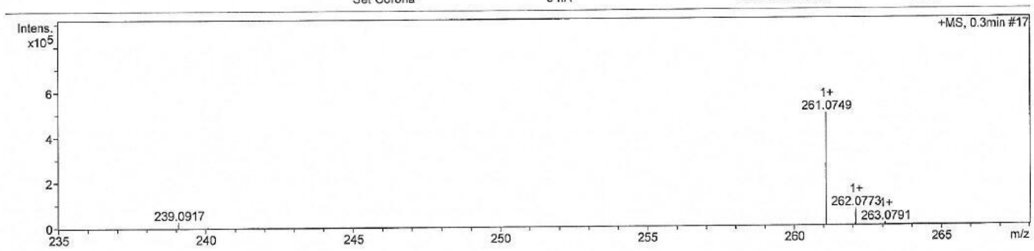

Mass Spectrum SmartFormula Report

**Analysis Info**

| | | | |
|---|---|---|---|
| Analysis Name | D:\Data\MS\data\201708\luoxiaowei_lxw-DI61_pos_73_01_3402.d | Acquisition Date | 8/22/2017 11:55:31 AM |
| Method | LC_DirectInfusion_pos_70-500mz.m | Operator | SCSIO |
| Sample Name | luoxiaowei_lxw-DI61_pos | Instrument | maXis 255552.00029 |
| Comment | | | |

**Acquisition Parameter**

| | | | | | |
|---|---|---|---|---|---|
| Source Type | ESI | Ion Polarity | Positive | Set Nebulizer | 0.4 Bar |
| Focus | Active | Set Capillary | 4500 V | Set Dry Heater | 180 °C |
| Scan Begin | 70 m/z | Set End Plate Offset | -500 V | Set Dry Gas | 4.0 l/min |
| Scan End | 1500 m/z | Set Charging Voltage | 0 V | Set Divert Valve | Waste |
| | | Set Corona | 0 nA | Set APCI Heater | 0 °C |



| Meas. m/z | # | Ion Formula | Score | m/z | err [ppm] | err [mDa] | mSigma | rdb | e⁻ Conf | N-Rule |
|---|---|---|---|---|---|---|---|---|---|---|
| 239.091660 | 1 | C12H15O5 | 100.00 | 239.091400 | 1.1 | 0.3 | 30.5 | 5.5 | even | ok |
| 261.074856 | 1 | C12H14NaO5 | 100.00 | 261.073344 | -5.8 | -1.5 | 8.0 | 5.5 | even | ok |
| 499.158964 | 1 | C24H28NaO10 | 100.00 | 499.157468 | -3.0 | -1.5 | 13.8 | 10.5 | even | ok |

**Figure S55.** HR-ESI-MS spectrum of methyl convolvulopyrone (**21**)

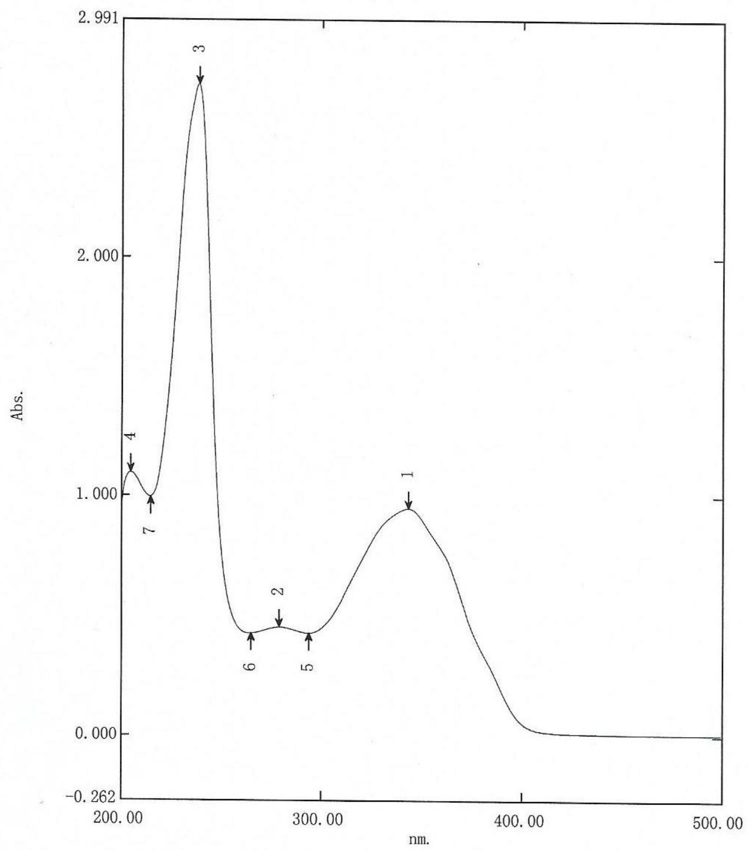


[测定属性]
波长范围 (nm.): 200.00 到 500.00
扫描速度: 中速
采样间隔: 0.2
自动采样间隔: 启用
扫描模式: 单个

[仪器属性]
仪器类型: UV-2600 系列
测定方式: 吸收值
狭缝宽: 2.0
积分时间: 0.1 秒.
光源转换波长: 323.0 nm
检测器单元: 直接
S/R 转换: 标准
阶梯校正: OFF

[附件属性]
附件: 无

[数据处理参数]
阈值: 0.0100000
点: 4
内插: 停用
平均: 停用

[样品准备属性]
重量:
体积:
稀释:
光程长:
附加信息:

Mr. 238.1 20ug/ml

| No. | P/V | 波长(nm) | 吸收值 | 描述 logε |
|---|---|---|---|---|
| 1 | | 342.80 | 0.952 | 4.054 |
| 2 | | 279.00 | 0.453 | 3.733 |
| 3 | | 238.60 | 2.720 | 4.510 |
| 4 | | 204.60 | 1.100 | 4.117 |

**Figure S56.** UV spectrum of methyl convolvulopyrone (**21**)

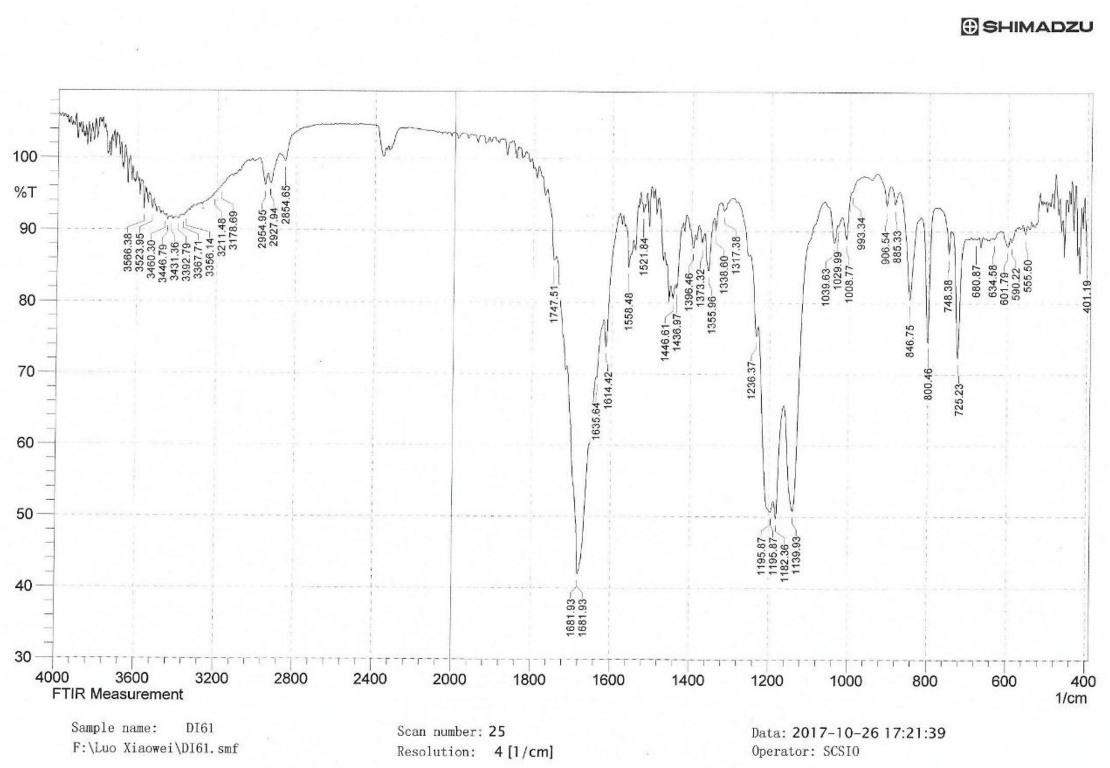


**Figure S57.** IR spectrum of methyl convolvulopyrone (**21**)

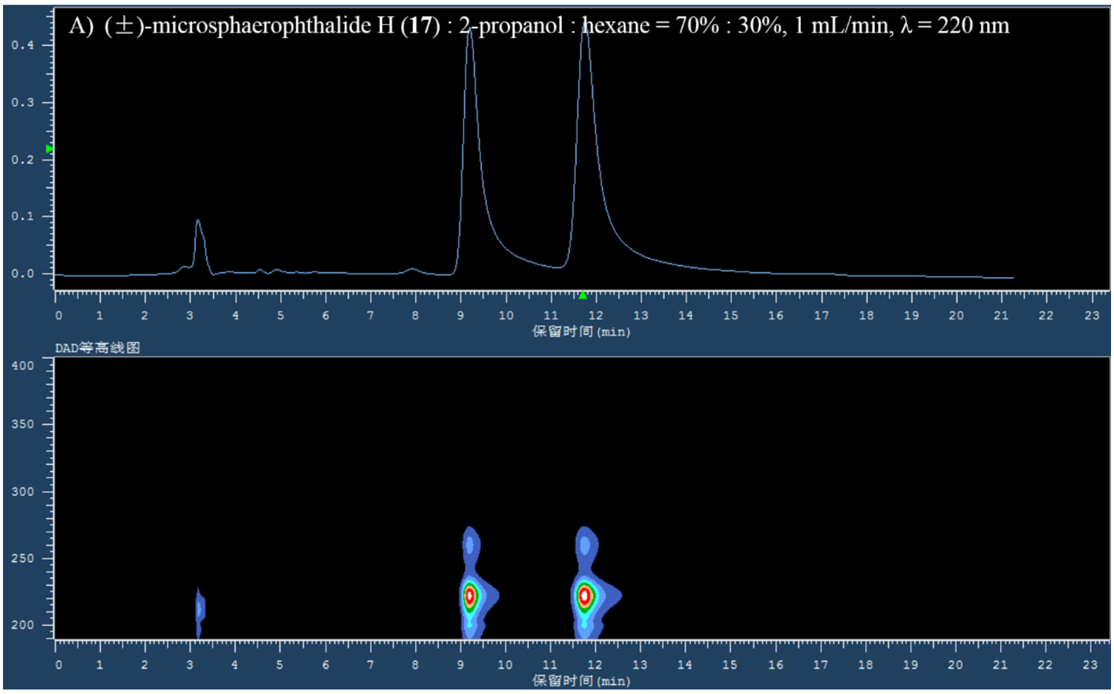

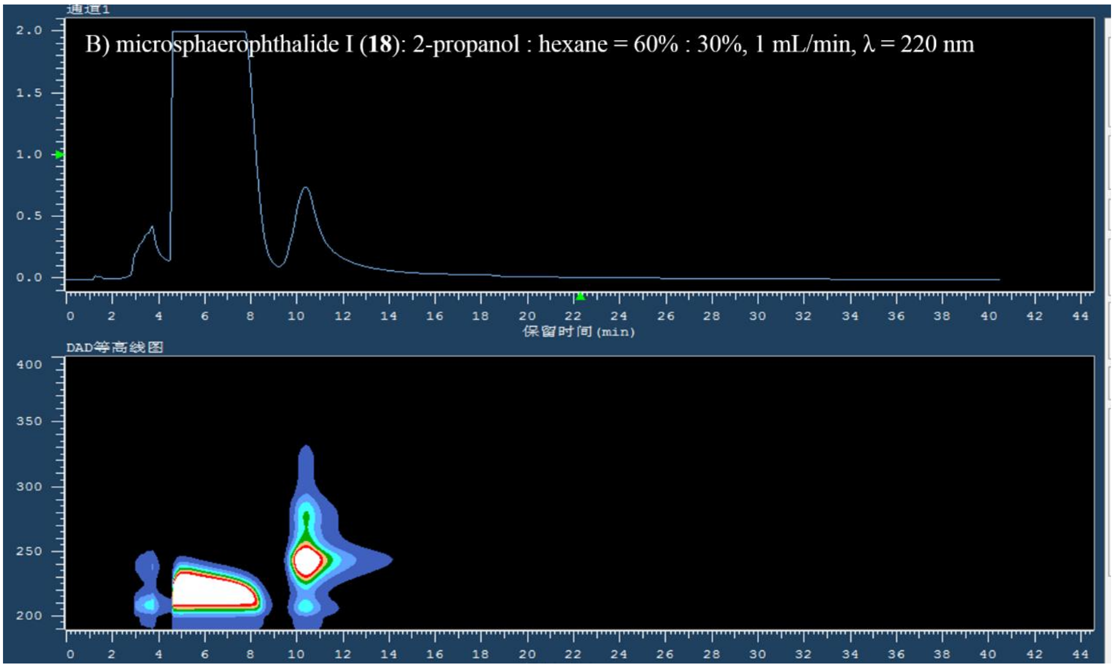


**Figure S58.** Chiral HPLC analysis of **17** (A, dissolved in MeOH) and **18** (B, DMSO) using CHIRALPAK IC column (250 × 4.6 mm, 5 $\mu$m).

**ECD calculation.**

The calculated ECD spectrum of compound **18** was according to our recent published literature (Luo et al., 2018). Briefly, compound **18** was subjected to random conformational searches by means of the Spartan 14 software using the Merck Molecular Force Field (MMFF) method. The conformers with Boltzmann-population of over 5% (the relative energy within 6 kcal/mol) were chosen for ECD calculations using Gaussian 09 software. The ECD spectra of different conformers were generated using the program SpecDis 1.6 (University of Würzburg) and Prism 5.0 (GraphPad Software Inc.) with a half-bandwidth of 0.3 eV and UV shift of – 15 nm, according to the Boltzmann-calculated contribution of each conformer after UV correction.

**Table S1. Energies of 18 at MMFF94 force field.**

| Configuration | Conformer | Energy (kcal/mol) | Population (%) |
|---|---|---|---|
| (3*R*)-**18** | 1 | 51.88 | 56.6 |
| (3*R*)-**18** | 2 | 53.46 | 30.0 |
| (3*R*)-**18** | 3 | 58.05 | 4.7 |
| (3*S*)-**18** | 1 | 51.88 | 56.6 |
| (3*S*)-**18** | 2 | 53.46 | 30.0 |
| (3*S*)-**18** | 3 | 58.05 | 4.7 |
| (3*S*)-**18** | 4 | 58.63 | 3.7 |

**Table S2. Energies of 18 at B3LYP/6–31+g(d, p) level in methanol.**

| Configuration | Conformer | E (Hartree) | E (kcal/mol) | Population (%) |
|---|---|---|---|---|
| (3*R*)-**18** | 1 | –1030.6614826 | –646750.39 | 46.31 |
| (3*R*)-**18** | 2 | –1030.6612844 | –646750.26 | 37.53 |
| (3*R*)-**18** | 3 | –1030.6604891 | –646749.76 | 16.15 |
| (3*S*)-**18** | 1 | –1030.6614825 | –646750.39 | 39.22 |
| (3*S*)-**18** | 2 | –1030.6612843 | –646750.26 | 31.78 |
| (3*S*)-**18** | 3 | –1030.6604888 | –646749.76 | 13.68 |
| (3*S*)-**18** | 4 | –1030.660596 | –646749.83 | 15.32 |

(3*R*)-**18**

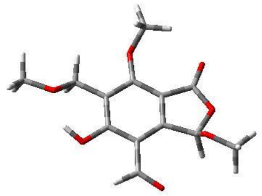

Conf.1 (46.31%)

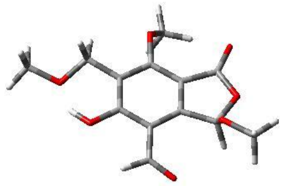

Conf.2 (37.53%)

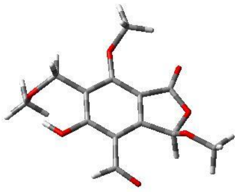

Conf.3 (16.15%)

(3*S*)-**18**

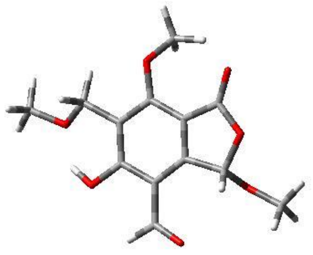

Conf.1 (39.22%)

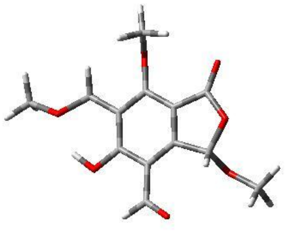

Conf.2 (31.78%)

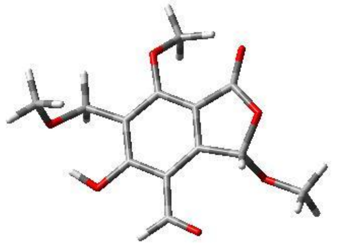

Conf.3 (13.68%)

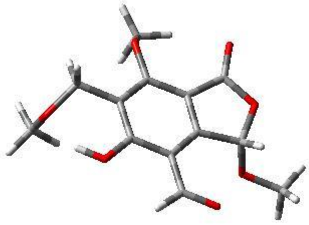

Conf.4 (15.32%)

**Figure S59.** The optimized conformers and equilibrium populations of microsphaerophthalide I (**18**)

*The physicochemical data of the known compounds **3–12**, **14–16**, **19–20**, **22–28**.*

*16-acetoxydothiorelone C(**3**)*: colorless oil; $^{1}$H NMR ($CD_3OD$, 700 MHz): $\delta_H$ 3.58 (2H, s, H-2), 6.17 (1H, d, $J$ = 2.2 Hz, H-4), 6.22 (1H, d, $J$ = 2.2 Hz, H-6), 2.85 (2H, t, $J$ = 7.6 Hz, H-10), 1.61 (2H, m,

H-11), 1.35 (2H, overlapped, H-12), 1.35 (2H, overlapped, H-13), 1.35 (2H, overlapped, H-14), 1.61 (2H, m, H-15), 4.05 (2H, t, *J* =7.1 Hz, H-16), 4.12 (2H, q, *J* = 7.1 Hz, H-1′), 1.24 (3H, t, *J* = 7.1 Hz, H-2′), 2.02 (3H, s, H-2″). $^{13}$C NMR ($CD_3OD$, 175MHz) $\delta_C$: 173.7 (qC, C-1), 40.8 ($CH_2$, C-2), 137.2 (qC, C-3), 112.5 (CH, C-4), 162.9 (qC, C-5), 103.1 (CH, C-6), 160.2 (qC, C-7), 120.4 (qC, C-8), 208.8 (qC, C-9), 45.0 ($CH_2$, C-10), 25.5 ($CH_2$, C-11), 30.3 ($CH_2$, C-12), 30.2 ($CH_2$, C-13), 26.9 ($CH_2$, C-14), 29.6 ($CH_2$, C-15), 65.7 ($CH_2$, C-16), 61.8 ($CH_2$, C-1′), 14.5 ($CH_3$, C-2′), 173.1 (qC, C-1″), 20.8 ($CH_3$, C-2″). HRESIMS *m/z* 403.1729 $[M+Na]^+$ (calcd for $C_{20}H_{28}NaO_7$, 403.1733), 419.1465 $[M+K]^+$ (calcd for $C_{20}H_{28}KO_7$, 419.1472).

*Dothiorelone C(**4**):* colorless oil; $^1$H NMR ($CD_3OD$, 700 MHz): $\delta_H$ 3.58 (2H, s, H-2), 6.20 (1H, d, *J* = 2.2 Hz, H-4), 6.25 (1H, d, *J* = 2.2 Hz, H-6), 2.90 (2H, t, *J* = 7.6 Hz, H-10), 1.60 (2H, m, H-11), 1.31 (2H, overlapped, H-12), 1.31 (2H, overlapped, H-13), 1.31 (2H, overlapped, H-14), 1.53 (2H, m, H-15), 3.54 (2H, t, *J* = 7.1 Hz, H-16), 4.12 (2H, q, *J* = 7.1 Hz, H-1′), 1.25 (3H, t, *J* = 7.1 Hz, H-2′). $^{13}$C NMR ($CD_3OD$, 175MHz) $\delta_C$: 173.6 (qC, C-1), 40.6 ($CH_2$, C-2), 137.1 (qC, C-3), 111.9 (CH, C-4), 161.7 (qC, C-5), 102.8 (CH, C-6), 160.0 (qC, C-7), 121.0 (qC, C-8), 209.0 (qC, C-9), 45.2 ($CH_2$, C-10), 25.4 ($CH_2$, C-11), 30.4 ($CH_2$, C-12), 30.4 ($CH_2$, C-13), 26.8 ($CH_2$, C-14), 33.6 ($CH_2$, C-15), 63.0 ($CH_2$, C-16), 61.8 ($CH_2$, C-1′), 14.5 ($CH_3$, C-2′). LRESIMS *m/z* 361.2 $[M+Na]^+$, 699.1 $[2M+Na]^+$, 337.1 $[M–H]^–$, 373.0 $[M+Cl]^–$.

*(14R)-acetoxydothiorelone B(**5**)*: colorless oil; $[\alpha]_D^{25}$ – 3.5 (*c* 0.17, MeOH); $^1$H NMR ($CD_3OD$, 700 MHz): $\delta_H$ 3.58 (2H, s, H-2), 6.19 (1H, d, *J* = 2.2 Hz, H-4), 6.26 (1H, d, *J* = 2.2 Hz, H-6), 2.91 (2H, t, *J* = 7.6 Hz, H-10), 1.60 (2H, m, H-11), 1.33 (2H, m, H-12), 1.55 (2H, m, H-13), 4.79 (1H, m, H-14), 1.55 (2H, m, H-15), 0.88 (3H, t, *J* = 7.1 Hz, H-16), 4.11 (2H, q, *J* = 7.1 Hz, H-1′), 1.24 (3H, t, *J* = 7.1 Hz, H-2′), 2.03 (3H, s, H-2″). $^{13}$C NMR ($CD_3OD$, 175MHz) $\delta_C$: 173.6 (qC, C-1), 40.6 ($CH_2$, C-2), 137.1 (qC, C-3), 111.9 (CH, C-4), 161.6 (qC, C-5), 102.8 (CH, C-6), 160.0 (qC, C-7), 121.0 (qC, C-8), 208.7 (qC, C-9), 45.0 ($CH_2$, C-10), 25.3 ($CH_2$, C-11), 26.2 ($CH_2$, C-12), 34.5 ($CH_2$, C-13), 76.9 (CH, C-14), 28.0 ($CH_2$, C-15), 10.0 ($CH_3$, C-16), 61.8 ($CH_2$, C-1′), 14.5 ($CH_3$, C-2′), 173.0 (qC, C-1″), 21.1 ($CH_3$, C-2″). LRESIMS *m/z* 403.2 $[M+Na]^+$, 419.2 $[M+K]^+$, 783.1 $[2M+Na]^+$; 379.1 $[M–H]^–$, 415.0 $[M+Cl]^–$, 759.1 $[2M–H]^–$; HRESIMS *m/z* 403.1740 $[M+Na]^+$ (calcd for $C_{20}H_{28}NaO_7$, 403.1733), 419.1471 $[M+K]^+$ (calcd for $C_{20}H_{28}KO_7$, 419.1472), 783.3561 $[2M+Na]^+$ (calcd for $C_{40}H_{56}NaO_{14}$, 783.3568).

*Cytosporone N (**6**):* colorless oil; $^1$H NMR ($CD_3OD$, 500 MHz): $\delta_H$ 3.58 (2H, s, H-2), 6.20 (1H, d,

$J$ = 2.2 Hz, H-4), 6.27 (1H, d, $J$ = 2.2 Hz, H-6), 2.90 (2H, t, $J$ = 7.6 Hz, H-10), 1.60 (2H, m, H-11), 1.31 (2H, overlapped, H-12), 1.31 (2H, overlapped, H-13), 1.31 (2H, overlapped, H-14), 1.31(2H, overlapped, H-15), 0.90 (3H, t, $J$ = 7.1 Hz, H-16), 3.65 (3H, s, H-1′). $^{13}$C NMR ($CD_3OD$, 125 MHz) $\delta_C$: 174.0 (qC, C-1), 40.3 ($CH_2$, C-2), 136.9 (qC, C-3), 111.8 (CH, C-4), 161.3 (qC, C-5), 102.8 (CH, C-6), 159.8 (qC, C-7), 121.2 (qC, C-8), 209.0 (qC, C-9), 45.2 ($CH_2$, C-10), 25.6 ($CH_2$, C-11), 30.5 ($CH_2$, C-12), 30.2 ($CH_2$, C-13), 32.9 ($CH_2$, C-14), 23.7 ($CH_2$, C-15), 14.4 ($CH_3$, C-16), 52.3 ($CH_3$, C-1′).

*Cytosporone B (**7**):* colorless oil; $^1$H NMR ($CD_3OD$, 500 MHz): $\delta_H$ 3.57 (2H, s, H-2), 6.20 (1H, d, $J$ = 2.2 Hz, H-4), 6.27 (1H, d, $J$ = 2.2 Hz, H-6), 2.90 (2H, t, $J$ = 7.6 Hz, H-10), 1.60 (2H, m, H-11), 1.31 (2H, overlapped, H-12), 1.31 (2H, overlapped, H-13), 1.31 (2H, overlapped, H-14), 1.31(2H, overlapped, H-15), 0.89 (3H, t, $J$ = 7.1 Hz, H-16), 4.11 (2H, q, $J$ = 7.1 Hz, H-1′), 1.23 (3H, t, $J$ = 7.1 Hz, H-2′). $^{13}$C NMR ($CD_3OD$, 125 MHz) $\delta_C$: 173.5 (qC, C-1), 40.5 ($CH_2$, C-2), 136.9 (qC, C-3), 111.7 (CH, C-4), 161.3 (qC, C-5), 102.7 (CH, C-6), 159.8 (qC, C-7), 121.2 (qC, C-8), 209.0 (qC, C-9), 45.2 ($CH_2$, C-10), 25.5 ($CH_2$, C-11), 30.4 ($CH_2$, C-12), 30.2 ($CH_2$, C-13), 32.9 ($CH_2$, C-14), 23.6 ($CH_2$, C-15), 14.4 ($CH_3$, C-16), 61.8 ($CH_2$, C-1′), 14.5 ($CH_3$, C-2′).

*Cytosporone A(**8**):* colorless oil; $^1$H NMR ($CD_3OD$, 500 MHz): $\delta_H$ 3.57 (2H, s, H-2), 6.21 (1H, d, $J$ = 2.2 Hz, H-4), 6.26 (1H, d, $J$ = 2.2 Hz, H-6), 2.91 (2H, t, $J$ = 7.6 Hz, H-10), 1.62 (2H, m, H-11), 1.31 (2H, overlapped, H-12), 1.31 (2H, overlapped, H-13), 1.31 (2H, overlapped, H-14), 1.31(2H, overlapped, H-15), 0.89 (3H, t, $J$ = 7.1 Hz, H-16). $^{13}$C NMR ($CD_3OD$, 125 MHz) $\delta_C$: 175.4 (qC, C-1), 40.5 ($CH_2$, C-2), 137.2 (qC, C-3), 111.6 (CH, C-4), 161.3 (qC, C-5), 102.6 (CH, C-6), 159.8 (qC, C-7), 121.3 (qC, C-8), 209.6 (qC, C-9), 45.2 ($CH_2$, C-10), 25.5 ($CH_2$, C-11), 30.4 ($CH_2$, C-12), 30.2 ($CH_2$, C-13), 32.8 ($CH_2$, C-14), 23.6 ($CH_2$, C-15), 14.4 ($CH_3$, C-16).

*Dothiorelone I (**9**):* colorless oil; $^1$H NMR ($CD_3OD$, 700 MHz): $\delta_H$ 3.60 (2H, s, H-2), 6.21 (1H, d, $J$ = 2.2 Hz, H-4), 6.27 (1H, d, $J$ = 2.2 Hz, H-6), 2.93 (2H, t, $J$ = 7.6 Hz, H-10), 1.65 (2H, m, H-11), 1.33 (2H, m, H-12), 1.59 (2H, m, H-13), 2.50 (2H, t, $J$ = 7.1 Hz, H-14), 2.14 (3H, s, H-16), 4.14 (2H, q, $J$ = 7.1 Hz, H-1′), 1.26 (3H, t, $J$ = 7.1 Hz, H-2′). $^{13}$C NMR ($CD_3OD$, 175 MHz) $\delta_C$: 173.6 (qC, C-1), 40.5 ($CH_2$, C-2), 137.1 (qC, C-3), 111.8 (CH, C-4), 161.6 (qC, C-5), 102.8 (CH, C-6), 159.9 (qC, C-7), 121.1 (qC, C-8), 208.8 (qC, C-9), 44.9 ($CH_2$, C-10), 25.2 ($CH_2$, C-11), 29.9 ($CH_2$, C-12), 24.7 ($CH_2$, C-13), 44.1 ($CH_2$, C-14), 212.2 (qC, C-15), 29.8 ($CH_3$, C-16), 61.8 ($CH_2$, C-1′), 14.5 ($CH_3$, C-2′). LRESIMS $m/z$ 359.2 $[M+Na]^+$, 695.1 $[2M+Na]^+$, 335.1 $[M–H]^–$, 371.1 $[M+Cl]^–$,

671.1 $[2M–H]^-$.

*(15R)-dothiorelone A (**10**):* colorless oil; $^1$H NMR ($CD_3OD$, 700 MHz): $\delta_H$ 3.59 (2H, s, H-2), 6.21 (1H, d, *J* = 2.2 Hz, H-4), 6.27 (1H, d, *J* = 2.2 Hz, H-6), 2.92 (2H, t, *J* = 7.6 Hz, H-10), 1.63 (2H, m, H-11), 1.37 (2H, overlapped, H-12), 1.37 (2H, overlapped, H-13), 1.64 (2H, overlapped, H-14), 3.97 (1H, m, H-15), 1.19 (3H, d, *J* = 7.1 Hz, H-16), 4.13 (2H, q, *J* = 7.1 Hz, H-1′), 1.27 (3H, t, *J* = 7.1 Hz, H-2′). $^{13}$C NMR ($CD_3OD$, 175MHz) $\delta_C$: 173.6 (qC, C-1), 40.6 ($CH_2$, C-2), 137.1 (qC, C-3), 111.9 (CH, C-4), 161.6 (qC, C-5), 102.8 (CH, C-6), 160.0 (qC, C-7), 121.1 (qC, C-8), 209.0 (qC, C-9), 45.2 ($CH_2$, C-10), 25.4 ($CH_2$, C-11), 30.4 ($CH_2$, C-12), 26.8 ($CH_2$, C-13), 33.6 ($CH_2$, C-14), 67.2 (CH, C-15), 23.5 ($CH_3$, C-16), 61.8 ($CH_2$, C-1′), 14.5 ($CH_3$, C-2′). LRESIMS *m/z* 361.2 $[M+Na]^+$, 699.2 $[2M+Na]^+$, 337.1 $[M–H]^-$, 373.1 $[M+Cl]^-$, 675.2 $[2M–H]^-$, 711.1 $[2M+Cl]^-$.

*Methyl (R)-2-(2-(7-acetoxyoctanoyl)-3,5-dihydroxyphenyl)acetate (**11**):* colorless oil; $^1$H NMR ($CD_3OD$, 700 MHz): $\delta_H$ 3.60 (2H, s, H-2), 6.19 (1H, d, *J* = 2.2 Hz, H-4), 6.26 (1H, d, *J* = 2.2 Hz, H-6), 2.83 (2H, t, *J* = 7.6 Hz, H-10), 1.62 (2H, m, H-11), 1.34 (2H, overlapped, H-12), 1.34 (2H, overlapped, H-13), 1.52 (2H, m, H-14), 4.86 (1H, m, H-15), 1.22 (3H, d, *J* =7.1 Hz, H-16), 3.67 (3H, s, H-1′), 2.02 (3H, s, H-2″). $^{13}$C NMR ($CD_3OD$, 175MHz) $\delta_C$: 174.1 (qC, C-1), 40.5 ($CH_2$, C-2), 137.1 (qC, C-3), 112.3 (CH, C-4), 162.3 (qC, C-5), 103.0 (CH, C-6), 160.3 (qC, C-7), 120.6 (qC, C-8), 208.8 (qC, C-9), 43.3 ($CH_2$, C-10), 25.4 ($CH_2$, C-11), 30.2 ($CH_2$, C-12), 26.3 ($CH_2$, C-13), 36.8 ($CH_2$, C-14), 72.4 (CH, C-15), 20.2 ($CH_3$, C-16), 52.3 ($CH_3$, C-1′), 172.8 (qC, C-1″), 21.2 ($CH_3$, C-2″). LRESIMS *m/z* 389.2 $[M+Na]^+$, 755.2 $[2M+Na]^+$, 365.1 $[M–H]^-$.

*Secocurvularin (**12**):* colorless oil; $^1$H NMR ($CD_3OD$, 700 MHz): $\delta_H$ 3.57 (2H, s, H-2), 6.20 (1H, d, *J* = 2.2 Hz, H-4), 6.26 (1H, d, *J* = 2.2 Hz, H-6), 2.90 (2H, t, *J* = 7.6 Hz, H-10), 1.61 (2H, m, H-11), 1.32 (2H, overlapped, H-12), 1.32 (2H, overlapped, H-13), 0.91 (3H, t, *J* = 7.1 Hz, H-14), 4.11 (2H, q, *J* = 7.1 Hz, H-1′), 1.24 (3H, t, *J* = 7.1 Hz, H-2′). $^{13}$C NMR ($CD_3OD$, 175 MHz) $\delta_C$: 173.6 (qC, C-1), 40.5 ($CH_2$, C-2), 136.9 (qC, C-3), 111.7 (CH, C-4), 161.3 (qC, C-5), 102.8 (CH, C-6), 159.8 (qC, C-7), 121.3 (qC, C-8), 209.0 (qC, C-9), 45.2 ($CH_2$, C-10), 25.2 ($CH_2$, C-11), 32.8 ($CH_2$, C-12), 23.6 ($CH_2$, C-13), 14.3 ($CH_3$, C-14), 61.9 ($CH_2$, C-1′), 14.5 ($CH_3$, C-2′).

*Pestalotiopsone F(**14**):* colorless oil; $^1$H NMR ($CD_3OD$, 700 MHz): $\delta_H$ 4.04 (2H, s, H-2), 6.59 (1H, d, *J* = 2.2 Hz, H-4), 6.64 (1H, d, *J* = 2.2 Hz, H-6), 5.94 (1H, s, H-10), 2.59 (2H, t, *J* = 7.1 Hz, H-12), 1.72 (2H, m, H-13), 1.38 (2H, overlapped, H-14), 1.38 (2H, overlapped, H-15), 0.93 (3H, t, *J* =7.1 Hz, H-16), 3.66 (3H, s, H-1′). $^{13}$C NMR ($CD_3OD$, 175 MHz) $\delta_C$: 174.2 (qC, C-1), 41.9 ($CH_2$,

C-2), 138.0 (qC, C-3), 121.6 (CH, C-4), 167.7 (qC, C-5), 103.5 (CH, C-6), 161.8 (qC, C-7), 113.9 (qC, C-8), 181.4 (qC, C-9), 110.1 (CH, C-10),169.9 (qC, C-11), 34.5 ($CH_2$, C-12), 27.6 ($CH_2$, C-13), 32.2 ($CH_2$, C-14), 23.4 ($CH_2$, C-15), 14.3 ($CH_3$, C-16), 52.2 ($CH_3$, C-1′).

*Pestalotiopsone B(**15**):* colorless oil; $^1$H NMR ($CD_3OD$, 700 MHz): $\delta_H$ 4.02 (2H, s, H-2), 6.57 (1H, d, *J* = 2.2 Hz, H-4), 6.62 (1H, d, *J* = 2.2 Hz, H-6), 5.93 (1H, s, H-10), 2.59 (2H, t, *J* = 7.1 Hz, H-12), 1.72 (2H, m, H-13), 1.39 (2H, overlapped, H-14), 1.39 (2H, overlapped, H-15), 1.31 (2H, overlapped, H-16), 1.31 (2H, overlapped, H-17), 0.90 (3H, t, *J* = 7.1 Hz, H-18), 4.13 (2H, q, *J* =7.1 Hz, H-1′), 1.24 (3H, t, *J* = 7.1 Hz, H-2′). $^{13}$C NMR ($CD_3OD$, 175 MHz) $\delta_C$: 173.8 (qC, C-1), 42.3 ($CH_2$, C-2), 138.0 (qC, C-3), 121.8 (CH, C-4), 168.1 (qC, C-5), 103.5 (CH, C-6), 161.9 (qC, C-7), 113.8 (qC, C-8), 181.3 (qC, C-9), 110.0 (CH, C-10),169.8 (qC, C-11), 34.6 ($CH_2$, C-12), 27.9 ($CH_2$, C-13), 30.1 ($CH_2$, C-14), 30.0 ($CH_2$, C-15), 32.9 ($CH_2$, C-16), 23.7 ($CH_2$, C-17), 14.5 ($CH_3$, C-18), 61.6 ($CH_2$, C-1′), 14.4 ($CH_3$, C-2′).

*Pestalotiopsone A(**16**):* colorless oil; $^1$H NMR ($CD_3OD$, 700 MHz): $\delta_H$ 4.07 (2H, s, H-2), 6.66 (1H, d, *J* = 2.2 Hz, H-4), 6.74 (1H, d, *J* = 2.2 Hz, H-6), 5.98 (1H, s, H-10), 2.61 (2H, t, *J* = 7.1 Hz, H-12), 1.73 (2H, m, H-13), 1.39 (2H, overlapped, H-14), 1.39 (2H, overlapped, H-15), 1.31 (2H, overlapped, H-16), 1.31 (2H, overlapped, H-17), 0.91 (3H, t, *J* = 7.1 Hz, H-18), 3.67 (3H, s, H-1′). $^{13}$C NMR ($CD_3OD$, 175 MHz) $\delta_C$: 174.0 (qC, C-1), 41.8 ($CH_2$, C-2), 138.5 (qC, C-3), 120.2 (CH, C-4), 164.6 (qC, C-5), 103.1 (CH, C-6), 161.4 (qC, C-7), 115.2 (qC, C-8), 181.4 (qC, C-9), 110.4 (CH, C-10), 170.4 (qC, C-11), 34.6 ($CH_2$, C-12), 27.9 ($CH_2$, C-13), 30.0 ($CH_2$, C-14), 30.0 ($CH_2$, C-15), 32.8 ($CH_2$, C-16), 23.6 ($CH_2$, C-17), 14.4 ($CH_3$, C-18), 52.2 ($CH_3$, C-1′).

5-hydroxy-7-methoxy-4,6-dimethylphthalide (**19**): white crystals; $^1$H NMR($CD_3OD$ , 500 MHz) $\delta_H$: 5.18(2H, s, H-3), 3.92(3H, s, 7-$OCH_3$), 2.15(3H, s, 6-$CH_3$), 2.11(3H, s, 4-$CH_3$);$^{13}$C NMR($CD_3OD$ , 500 MHz) $\delta_C$:172.1(qC, C-1), 69.8($CH_2$, C-3), 114.5(qC, C-4), 162.0 (qC, C-5), 119.7(qC, C-6), 157.4(qC, C-7), 148.2(qC, C-3a), 108.7 (qC, C-7a), 62.4($CH_3$, 7-$OCH_3$), 11.3($CH_3$, 4-$CH_3$), 9.1($CH_3$, 6-$CH_3$).

*Dihydrovermistatin (**20**):* white amorphous powder; $^1$H NMR (DMSO-$d_6$, 700 MHz): $\delta_H$ 6.93 (1H, d, *J* = 2.0 Hz, H-3), 6.87 (1H, d, *J* = 2.1 Hz, H-5), 6.27 (1H, s, H-8), 6.15 (1H, s, H-11), 8.24 (1H, s, H-14), 2.52 (2H, t, *J* = 7.0 Hz, H-15), 1.60 (2H, qt, *J* = 7.0, 7.7 Hz, H-16), 0.90 (3H, t, *J* = 7.0 Hz, H-17), 3.86 (3H, s, H-4-OMe), 3.76 (3H, s, H-6-OMe); $^{13}$C NMR (DMSO-$d_6$,175MHz) $\delta_C$: 169.7 (qC, C-1), 128.9 (qC, C-2), 98.9 (CH, C-3), 162.3(qC, C-4), 104.9 (CH, C-5), 154.7 (qC, C-6),

127.6 (qC, C-7), 74.5 (CH, C-8), 121.9 (qC, C-9),176.1 (qC, C-10), 113.9 (CH, C-11), 169.4 (qC, C-12), 156.4 (CH, C-14), 34.2 ($CH_2$, C-15), 19.5 ($CH_2$, C-16), 13.2 ($CH_3$, C-17), 56.1 ($CH_3$, OMe-4), 55.9 ($CH_3$, OMe-6).

*Sclerotinin A (**22**):* colorless oil; $^1$H NMR (DMSO-$d_6$, 500 MHz): $\delta_H$ 11.67 (1H, br s, 8-OH), 9.37 (1H, br s, 6-OH), 7.27 (1H, br s, 3-OH), 3.11 (1H, d, $J$ = 7.0 Hz, H-4), 1.59 (3H, s, Me-11), 1.05 (3H, d, $J$ = 7.0 Hz, Me-4), 2.03 (3H, s, Me-7), 2.02 (3H, s, Me-5); $^{13}$C NMR (DMSO-$d_6$, 125MHz): $\delta_C$ 169.7 (qC, C-1), 108.4 (qC, C-3), 38.3 (CH, C-4), 113.2 (qC, C-5), 159.1 (qC, C-6), 104.6 (qC, C-7), 160.3 (qC, C-8), 98.7 (qC, C-8a), 141.3 (qC, C-4a), 25.1 ($CH_3$, Me-3), 17.1 ($CH_3$, Me-4), 10.6 ($CH_3$, Me-5), 8.4 ($CH_3$, Me-7).

*3,5-dimethyl-8-hydroxy-3,4-dihydroisocoumarin (**23**):* colourless needles; $^1$H NMR (DMSO-$d_6$, 500 MHz): $\delta_H$ 10.94 (1H, br s, 8-OH), 7.37 (1H, d, $J$ = 8.5 Hz, H-6), 6.78 (1H, d, $J$ = 8.5 Hz, H-7), 4.72 (1H, m, H-3), 2.70 (1H, dd, $J$ = 16.8, 11.9 Hz, H-4a), 3.04 (1H, dd, $J$ = 16.8, 2.8 Hz, H-4b), 2.15 (3H, s, 5-$CH_3$), 1.44 (3H, d, $J$ = 7.2 Hz, 3-$CH_3$). $^{13}$C NMR (DMSO-$d_6$,125MHz) $\delta_C$: 169.8 (qC, C-1), 75.4 (CH, C-3), 31.0 ($CH_2$, C-4), 138.1 (qC, C-4a), 125.1 (qC, C-5), 137.7 (CH, C-6), 114.8 (CH, C-7), 159.4 (qC, C-8), 108.0 (qC, C-8a), 20.4 ($CH_3$, Me-3), 17.6 ($CH_3$, Me-5).

*3,5-dimethyl-8-methoxy-3,4-dihydroisocoumarin (**24**):* colourless needles; $^1$H NMR ($CD_3OD$, 700 MHz): $\delta_H$ 7.42 (1H, d, $J$ = 8.4 Hz, H-6), 6.97 (1H, d, $J$ = 8.4 Hz, H-7), 4.53 (1H, m, H-3), 3.86 (3H, s, 8-$OCH_3$), 2.70 (1H, dd, $J$ =16.8, 11.9 Hz, H-4a), 3.03 (1H, dd, $J$ = 16.8, 2.8 Hz, H-4b), 2.24 (3H, s, 5-$CH_3$), 1.48 (3H, d, $J$ = 7.2 Hz, 3-$CH_3$). $^{13}$C NMR ($CD_3OD$,175MHz) $\delta_C$: 166.0 (qC, C-1), 75.5 (CH, C-3), 33.7($CH_2$, C-4), 141.9 (qC, C-4a), 127.9 (qC, C-5), 137.5 (CH, C-6), 111.7 (CH, C-7), 160.8 (qC, C-8), 114.1 (qC, C-8a), 20.8 ($CH_3$, Me-3), 18.4 ($CH_3$, Me-5), 56.3 ($CH_3$, OMe-8).

*Methyl 8-hydroxy-6-methyl-9-oxo-9H-xanthene-1-carboxylate (**25**):* yellow, amorphous powder; $^1$H NMR ($CD_3OD$, 700 MHz): $\delta_H$ 7.74 (1H, d, $J$ = 7.7 Hz, H-2), 7.79 (1H, t, $J$ = 7.7 Hz, H-3), 7.44 (1H, d, $J$ = 7.7 Hz, H-4), 6.88 (1H, s, H-5), 6.66 (1H, s, H-7), 2.40 (3H, s, H-10), 3.90 (3H, s, H-12); $^{13}$C NMR ($CD_3OD$,175MHz) $\delta_C$: 133.0 (qC, C-1), 119.5 (CH, C-2), 135.8 (CH, C-3), 122.7(CH, C-4), 155.4 (qC, C-4a), 155.4 (qC, C-5a), 106.9 (CH, C-5), 149.4 (qC, C-6), 111.7 (CH, C-7), 161.2 (qC, C-8), 106.6 (qC, C-8a),179.7 (qC, C-9),116.8 (qC, C-9a), 22.1 ($CH_3$, C-10), 168.7 (qC, C-11), 52.7 ($CH_3$, C-12).

*3,8-dihydroxy-6-methyl-9-oxo-9H-xanthene-1-carboxylate (**26**):* yellow, amorphous powder; ; $^1$H NMR (DMSO-$d_6$, 700 MHz): $\delta_H$ 6.68 (1H, d, $J$ = 2.0 Hz, H-2), 6.74 (1H, overlapped, H-4), 6.74

(1H, overlapped, H-5), 6.56 (1H, s, H-7), 2.40 (3H, s, H-10), 3.94 (3H, s, H-12); $^{13}$C NMR (DMSO-$d_6$,175MHz) $\delta_C$: 136.4 (qC, C-1), 115.3 (CH, C-2), 169.0 (qC, C-3), 104.7(CH, C-4), 159.9 (qC, C-4a), 157.2 (qC, C-5a), 108.2 (CH, C-5), 149.6 (qC, C-6), 112.0 (CH, C-7), 162.5 (qC, C-8), 107.3 (qC, C-8a),180.6 (qC, C-9),109.6 (qC, C-9a), 22.4 ($CH_3$, C-10), 171.8 (qC, C-11), 53.4 ($CH_3$, C-12). LRESIMS *m/z* 301.1 $[M+H]^+$, 323.1 $[M+Na]^+$,623.3 $[2M+Na]^+$, 299.1 $[M–H]^–$, 599.3 $[2M–H]^–$.

*Pinselin (**27**):* reddish brown amorphous powder; $^1$H NMR (DMSO-$d_6$, 700 MHz) $\delta_H$ 7.40 (1H, d, $J$ = 8.2 Hz, H-3), 7.54(1H, d, $J$ = 8.2 Hz, H-4), 6.88 (1H, s, H-5), 6.63 (1H, s, H-7), 2.39 (3H, s, H-10), 3.82 (3H, s, H-12). $^{13}$C NMR(DMSO-$d_6$, 175 MHz) $\delta_C$ 116.7 (qC, C-1), 117.3 (qC, C-9a), 148.9 (qC, C-2), 126.2 (CH, C-3), 119.8 (CH, C-4),147.8 (qC, C-4a), 107.3 (CH, C-5), 155.4 (qC, C-5a), 147.7 (qC, C-6), 110.5 (CH, C-7), 160.4 (qC, C-8), 105.9 (qC,C-8a), 180.3 (qC, C-9), 22.0 ($CH_3$, C-10), 167.4 (qC, C-11), 52.0 ($CH_3$, C-12). LRESIMS *m/z* 301.2 $[M+H]^+$, 323.1 $[M+Na]^+$,623.1 $[2M+Na]^+$, 299.0 $[M–H]^–$, 335.0 $[M+Cl]^–$, 635.0 $[2M+Cl]^–$.

*7-hydroxy-2,5-dimethylchromone (**28**):* white amorphous powder; ; $^1$H NMR (DMSO-$d_6$, 500 MHz): $\delta_H$ 6.59 (1H, overlapped, H-6), 6.58 (1H, overlapped, H-8), 5.95 (1H, s, H-3), 2.26 (3H, s, 2-Me), 2.63 (3H, s, 5-Me); $^{13}$C NMR (DMSO-$d_6$, 125MHz) $\delta_C$: 178.2 (qC, C-4), 163.6 (qC, C-2), 161.7 (qC, C-7), 159.2 (qC, C-8a),141.2 (qC, C-5),116.8 (CH, C-3), 113.8 (qC, C-4a), 110.6 (CH, C-6), 100.5 (CH, C-8),22.4 ($CH_3$, Me-5), 19.3 ($CH_3$, Me-2).

## References

Luo, X., Lin, X., Tao, H., Wang, J., Li, J., Yang, B., et al. (2018). Isochromophilones A–F, cytotoxic chloroazaphilones from the marine mangrove endophyticfungus *Diaporthe* sp. SCSIO 41011. *J. Nat. Prod.* 81, 934-941. doi: 10.1021/acs.jnatprod.7b01053
